# Vaccination for the prevention of equine herpesvirus‐1 disease in domesticated horses: A systematic review and meta‐analysis

**DOI:** 10.1111/jvim.16895

**Published:** 2023-11-06

**Authors:** Klaus Osterrieder, David C. Dorman, Brandy A. Burgess, Lutz S. Goehring, Peggy Gross, Claire Neinast, Nicola Pusterla, Gisela Soboll Hussey, David P. Lunn

**Affiliations:** ^1^ Institut für Virologie Freie Universität Berlin Berlin Germany; ^2^ College of Veterinary Medicine North Carolina State University Raleigh North Carolina USA; ^3^ College of Veterinary Medicine University of Georgia Athens Georgia USA; ^4^ College of Agriculture, Food and Environment University of Kentucky, Maxwell H. Gluck Equine Research Center Lexington Kentucky USA; ^5^ School of Veterinary Medicine, University of California Davis California USA; ^6^ College of Veterinary Medicine, Michigan State University, Veterinary Medical Center East Lansing Michigan USA; ^7^ School of Veterinary Science, University of Liverpool Liverpool UK

**Keywords:** equine, equine herpesvirus myeloencephalopathy (EHM), equine herpesvirus‐1 (EHV‐1), vaccination

## Abstract

**Background:**

Equine herpes virus type 1 (EHV‐1) infection in horses is associated with respiratory and neurologic disease, abortion, and neonatal death.

**Hypothesis:**

Vaccines decrease the occurrence of clinical disease in EHV‐1‐infected horses.

**Methods:**

A systematic review was performed searching multiple databases to identify relevant studies. Selection criteria were original peer‐reviewed research reports that investigated the in vivo use of vaccines for the prevention of disease caused by EHV‐1 in domesticated horses. Main outcomes of interest included pyrexia, abortion, neurologic disease, viremia, and nasal shedding. We evaluated risk of bias, conducted exploratory meta‐analyses of incidence data for the main outcomes, and performed a GRADE evaluation of the quality of evidence for each vaccine subtype.

**Results:**

A total of 1018 unique studies were identified, of which 35 met the inclusion criteria. Experimental studies accounted for 31/35 studies, with the remainder being observational studies. Eight vaccine subclasses were identified including commercial (modified‐live, inactivated, mixed) and experimental (modified‐live, inactivated, deletion mutant, DNA, recombinant). Risk of bias was generally moderate, often because of underreporting of research methods, and sample sizes were small leading to imprecision in the estimate of the effect size. Several studies reported either no benefit or minimal vaccine efficacy for the primary outcomes of interest. Meta‐analyses revealed significant heterogeneity was present, and our confidence in the quality of evidence for most outcomes was low to moderate.

**Conclusions and Clinical Importance:**

Our review indicates that commercial and experimental vaccines minimally reduce the incidence of clinical disease associated with EHV‐1 infection.

**Abbreviations**
dpi: days post‐infection
EHV‐1: equine herpesvirus‐1
EHM: equine herpesvirus‐1 myeloencephalopathy
gD, gE: glycoprotein D, E, etc
GRADE: Grading of Recommendations, Assessment, Development, and Evaluation
ORF: open reading frame
PICO: Population, Intervention, Comparator, and Outcome
RCTs: randomized clinical trials
PFU: plaque‐forming units
PRISMA: Preferred Reporting Items for Systematic reviews and Meta‐Analyses

## 1 INTRODUCTION

Equine herpesvirus‐1 (EHV‐1) is a highly prevalent pathogen that infects horses worldwide.^1^ The virus is transmitted horse‐to‐horse through oronasal secretions as well as from contact with aborted fetuses, placenta, and fomites.^2^, ^3^ Following infection, EHV‐1 initially replicates in the epithelia of the upper respiratory tract.^4^, ^5^ This results in epithelial damage, serous nasal discharge as well as fever and nasal shedding of virus which peaks from 1 to 4 days postinfection (dpi). EHV‐1 is transferred to mononuclear immune cells in the retropharyngeal lymphatic tissues resulting in cell‐associated viremia in peripheral blood mononuclear cells (PBMC) and delivery of EHV‐1 to tissues including the spinal cord and uterus. Infection with EHV‐1 typically results in the establishment of a lifelong latent infection within the first months of life,^6^ with subsequent viral reactivation that can cause clinical disease and virus shedding.

In foals and yearlings, EHV‐1 infection causes upper respiratory tract disease with limited morbidity. The much more impactful disease outcomes are epidemic abortion in the last trimester of pregnancy and, in sporadic cases, equine herpesvirus myeloencephalopathy (EHM), a neurological disease characterized by ataxia, urinary incontinence, and paresis, which is more pronounced in the hindlimbs. Outbreaks of EHM have extensive impacts on the equine industry.^7^, ^8^, ^9^

Control of EHV‐1 in horses relies on a combination of vaccination, infection control, and management practices.^3^ Despite routine vaccination with commercially available killed and modified‐live vaccines (MLV) in equine veterinary practice, outbreaks of EHV‐1 disease continue to be reported. The goal of this study was to complete a systematic review of the scientific literature to assess the efficacy of vaccination for control of EHV‐1 infection in domesticated horses.

## 2 MATERIALS AND METHODS

### 2.1 Problem formulation and protocol development

A systematic review study protocol was developed using guidelines provided by the Cochrane Collaboration.^10^ The protocol detailed the research question, outcome of interest, outlined a search strategy and the process of data extraction, and provided criteria for rating the quality of evidence (Supporting Information Item 1). The specific review question and PICO (Population, Intervention, Comparator, and Outcome) statement for the systematic review are as follows:

- Review question: Does vaccination protect against EHV‐1 infection and disease?
- Disease definition: Clinical outcomes that result from EHV‐1 infection include one or more of the following:
  - Rhinopneumonitis: pyrexia with respiratory signs, including oculo‐nasal discharge, elevated respiratory rate, cough, lethargy.
  - Abortion in the third trimester.
  - Equine Herpesvirus Myeloencephalopathy (EHM).
  - Neonatal infection.
  - Ocular disease.
  - Male reproductive tract infection—orchitis.
- Population: Domesticated equids without sex, age, or breed restrictions.
- Intervention: EHV‐1 vaccination without restriction of vaccine type (eg, modified live virus) or vaccination protocol.
- Comparator: Equids experimentally infected or naturally exposed to EHV‐1 infection, after receiving placebo, or other vaccines, or unvaccinated animals.
- Outcome: All clinical outcomes that reflect symptomatic EHV‐1 infection or viral infection. Presence and degree of viral infection. Endpoints related to vaccine efficacy (relative reduction in EHV‐1 risk after vaccination) and effectiveness (reduction in odds of EHV‐1) associated with vaccination in an observational study, are relevant outcomes.

### 2.2 Study selection

Studies included in the systematic review were not restricted by either publication date, language, or quality. Only peer‐reviewed articles were considered for inclusion. Studies included in the review were randomized clinical trials (RCTs), nonrandomized intervention trials, and observational studies. Studies were included in the review only if they included a control or comparator group (either placebo‐treated or untreated controls). The following inclusion and exclusion criteria were used to select studies:

- Inclusion:
  - Domesticated equids without sex, age, breed, or immunological status restriction.
  - Vaccination trials that evaluated the efficacy of vaccines against EHV‐1 following experimental challenge or natural infection.
  - Studies that used a placebo or other vaccine or unvaccinated animals.
  - Studies that included clinical outcomes that reflect symptomatic EHV‐1 infection.
  - Endpoints related to vaccine efficacy: relative reduction in EHV‐1 disease risk; reduction in odds of EHV‐1 infection.
- Exclusion:
  - Absence of an EHV‐1 infection by experimental or natural infection.
  - Absence of the selected clinical or virological outcomes.
  - Wrong virus species.
  - Lack of a concurrent control or comparator.
  - Wrong animal species (not *Equus caballus*).
  - Purely descriptive observational studies.
  - No original data.

### 2.3 Search methods for identification of studies

Searches for relevant existing systematic reviews were performed initially to avoid duplicating any recent work or work in progress. PubMed and the systematic review protocol registries PROSPERO and CAMARADES were searched for systematic reviews. No previous relevant systematic reviews were found.

This systematic review followed the PRISMA (Preferred Reporting Items for Systematic Reviews and Meta‐Analyses) statement guidelines.^11^ The PubMed search was adapted for the following databases: Web of Science, Cab Abstracts, WHO Global Health Index Medicus Regional Databases, AGRICOLA (AGRICultural OnLine Access), and Cochrane (see Supporting Information Item 2). In conducting our search, we used a combination of controlled vocabulary and keywords for the following concepts: (1) EHV‐1, (2) horses, and (3) vaccination. We did not seek to identify research abstracts from meeting proceedings or unpublished studies because these are not commonly subjected to exhaustive peer review. We did not limit to language or publication date. All citations were imported into Covidence systematic review software (Veritas Health Innovation, Melbourne, Australia) for peer review by the research team. Titles and abstracts relevant to our study were retrieved and searched for full text. References from included studies were hand‐searched to identify any additional relevant studies for analysis. The literature search was initially conducted on December 18, 2019, and updated on August 12, 2020, and February 17, 2021. Literature searches were performed by a medical librarian and coauthor (Peggy Gross) with experience in the conduct of systematic reviews.

Retrieved references were independently screened at the title and abstract level and at the full‐text level for adherence to the PICO statement by two people (David C. Dorman and David P. Lunn) using Covidence software. At the title and abstract screening level, if there was disagreement between the reviewers or an abstract was not available, the reference was passed on to the full‐text screening level for further review. At the full‐text level, disagreements about whether to include a reference were discussed by the two reviewers (David C. Dorman and Lutz S. Goehring) to reach agreement; if consensus was not reached, then a third team member (Claire Neinast) resolved the differences. Coauthors of studies were excluded from evaluating their publications for inclusion or exclusion.

### 2.4 Data extraction

Two authors (David C. Dorman and Claire Neinast) performed data extraction using a customized data‐extraction form and working with two other individuals (Irene Nazario [see Supporting Information] and Klaus Osterrieder) verified the records for accuracy and completeness. Data items extracted included study design, characteristics of trial participants (number and breed of horses examined), vaccine characteristics (dose, route of administration, and timing of administration), virus challenge (dose, route of administration, and timing of administration), the type of control group used, outcomes measured, and study results. Extraction of graphical data relied on DigitizeIt version 2.5.1.

### 2.5 Methods of review

Risk of bias in individual studies was assessed by two authors (David C. Dorman and Claire Neinast) working independently of each other using the Covidence systematic review software. Coauthors of studies were excluded from evaluating their publications for risk of bias. Each member evaluated each study according to prespecified criteria developed for animal experiments and with the application of signaling questions provided by the risk of bias tool.^12^ The eight risk‐of‐bias domains used in this study included: random sequence generation; groups similar at baseline; allocation sequence; blinding of participants and personnel; blinding of outcome assessment; incomplete outcome data; selective reporting; and other sources of bias. Available risk‐of‐bias ratings for each domain were: low risk of bias; unknown risk of bias; or high risk of bias. Information or study procedures that were not reported were assumed not to have been conducted, resulting in an assessment of “unknown” risk of bias. Study authors were not contacted for missing data.

### 2.6 Method of analysis and evidence synthesis

MedCalc version 20.011 was used for statistical analysis. Forest plot analysis was used to evaluate interstudy heterogeneity for clinical outcomes. Results were reported as risk ratios (RR) comparing the incidence of a clinical outcome in experimental groups to control groups. A value of 0.5 was added to all cells when zeros in an incidence table led to computational errors.^13^ A random effect, Mantel‐Haenszel model (95% CI) was used to determine effect sizes between studies. Some studies included multiple arms where controls were shared between experimental groups. In this case, results were pooled or the incidence rates in the controls were apportioned between the different arms.^10^ Statistical heterogeneity was assessed using *I* ^2^ statistics: statistically significant *I* ^2^ values of ≥75% represented considerable heterogeneity, *I* ^2^ values <40% were deemed unimportant while intermediate values represented moderate heterogeneity.^10^ Random‐effect models were preferred over fixed‐effect models due to the evidence of high heterogeneity between trials when a fixed‐effect model was used. All meta‐analyses considered published equine studies that evaluated the relative risk in vaccine compared with control groups. Missing studies in the forest plot had all events in both intervention and control groups (relative risk = 1). These studies provide no information about the relative probability of the event and were automatically omitted from the meta‐analysis.^14^

Assessment of the quality, quantity, and consistency of evidence across studies was independently performed by two authors (David C. Dorman and Claire Neinast) using the Grading of Recommendations, Assessment, Development, and Evaluation (GRADE) approach.^15^ The GRADE approach was applied to the three main vaccine subclasses (live vaccines, inactivated vaccines, and other experimental vaccine) that had exploratory meta‐analyses. One study^16^ had one experimental arm that involved the administration of both a commercial MLV vaccine and an inactivated vaccine. The GRADE approach was not applied to this vaccination protocol.

The methods used in the present study were adapted from the GRADE approaches developed for animal studies by the National Institute of Environmental Health Sciences (NIEHS) Office of Health Assessment and Translation (OHAT) and adopted by the National Academies of Sciences, Engineering, and Medicine.^17^, ^18^ In brief, studies on a particular outcome were initially grouped by key study design features, and each grouping of studies was given an initial confidence rating based on those features. The initial confidence rating ranges from 0 to 4 with one point given for each of the following features: (a) controlled exposure; (b) exposure before outcome; (c) individual outcome data; and (d) comparison group used. Several factors were then considered to determine whether the initial rating should be either downgraded or upgraded. Factors that could downgrade the rating included quality, indirectness (use of surrogate outcomes), inconsistency (heterogeneity), and imprecision (wide confidence intervals around the effect). Factors that could upgrade the rating included large magnitude of effect, dose response, and accounting for plausible confounders. To obtain the final GRADE score for a given outcome, points were deducted from the initial GRADE score based on criteria related to the following four categories: quality, directness, consistency, and precision. Details regarding this step have been previously published.^10^ After a final confidence rating was determined, the rating was translated into a level of evidence using the following scheme: final score ≤1: very low; 2: low; 3: moderate; ≥4: high. Evidence profiles and summary‐of‐findings tables were created using a customized form.

## 3 RESULTS

### 3.1 Results of the search

**FIGURE 1:**
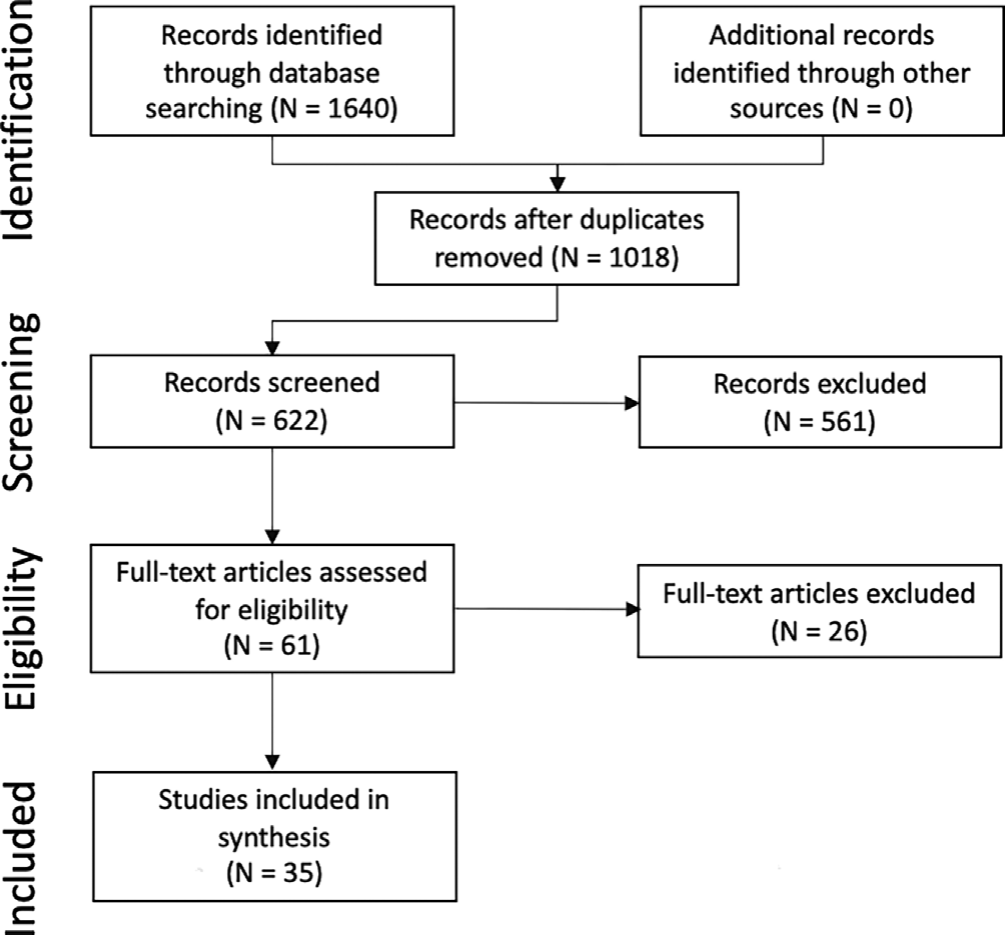
PRISMA diagram.

The search strategy identified 1640 citations, of which 622 were duplicate citations. Another 561 citations were excluded based on the title or abstract. Literature was entirely identified and retrieved from electronic bibliographic sources. No studies were identified from hand‐searching reference lists provided in the studies that met inclusion criteria. A total of 61 studies were assessed for inclusion using a review of the full text. A list of the 26 studies excluded at the full‐text review stage, with the reason for exclusion, is provided in Supporting Information Item 3. A total of 35 studies met the inclusion criteria for this review (Supporting Information Item 4). A flow diagram for inclusion of studies in the systematic review is provided in Figure 1.

### 3.2 Observational studies with natural infections

Four studies^9^, ^19^, ^20^, ^21^ were identified that involved natural infection with EHV‐1 (Table 1). Vaccines were unidentified in 2 of the studies,^9^, ^19^ and relative risks could not be calculated from the presented data. These studies were not incorporated into our subsequent synthesis of the results.

**TABLE 1:** Reviewed studies that evaluated use of commercially available vaccines for protection against EHV‐1 with a natural exposure model in domesticated horses.

Study	Study description	Vaccine	Main findings
Bannai et al^20^	Retrospective study evaluating immunologic responses and efficacy of two vaccines given to horses at an equine training facility in Japan.	Vaccine use was switched over a 1‐year period an inactivated EHV‐1 vaccine (Equine Rhinopneumonitis Inactivated Vaccine) to a MLV (Equi N Tect ERP). No information regarding vaccine administration was provided.	Total number of pyretic horses declines following the administration of the MLV vaccine.
Barrandeguy et al^19^	Case report describing vaccine efficacy during an outbreak of abortion attributed to EHV‐1 infection at two geographically separated breeding farms and an equine reproductive center in Argentina.	Three unidentified inactivated EHV‐1 vaccines were used during this outbreak. Vaccines were generally administered at 5, 7, and 9 months of gestation. One group of mares was vaccinated with vaccine 2 at 8 months of gestation.	Of a total of 173 pregnant mares present on the 3 affected premises, 50 (28%) lost their foals as a result of EHV‐1 infection. The abortion rate in mares, receiving the delayed vaccinations, was the highest (57%) versus 20% to 27% in the other groups.
Dutta and Shipley^21^	Case report describing vaccine efficacy in foals and pregnant mares on 3 farms (unknown location)	Animals were vaccinated with Rhinomune a MLV EHV‐1 vaccine according to manufacturer recommendations.	Foals were disease‐free for 6 months after vaccination. This was followed by an epizootic of respiratory tract disease. Abortions were seen at one farm only and abortions occurred in 4/15 mares. Another 4 foals died within 2 days of birth.
Traub‐Dargatz et al^9^	Case‐control study analyzing risk factors associated with an outbreak of EHM that occurred in the United States. May 2011 among horses that participated in a competitive event. EHM case survey data were compared with data from EHV‐1 cases with no neurologic signs and healthy controls.	The majority of EHM cases were reportedly vaccinated against EHV‐1 in the 12 months before the outbreak. Vaccine and administration were not provided.	EHV‐1 vaccination in the 5 weeks before the event was associated with an increased risk of EHM when compared with unaffected controls (OR = 3.36; CI: 1.20‐9.45; *P* = .02).

#### 3.2.1 Experimental virus challenge studies

Key study characteristics of the 31 experimental studies are provided in Table 2 with additional information provided in Table S1. One study^22^ is a partial follow‐up to a second study with additional analysis of cytotoxic T lymphocytes (CTL) in mares from the earlier study.^23^ Data from the later study^22^ was included in the meta‐analyses to prevent duplication of results. Experimental challenges used a variety of different EHV‐1 strains and included Ab4, Army 183, European strain 121412, and Findlay OH (OH03) among others. In several cases, the investigators did not identify the strain of virus used other than to state that it was isolated from an aborted fetus.^24^, ^25^ Doses of similar viruses between studies often varied by <2 orders of magnitude. For example, studies with the neuropathogenic strain Ab4 included studies using a range of challenge doses from 10^5^ to 10^6^ tissue‐culture infectious dose50 (TCID_50_) or plaque‐forming units (PFU). Most studies (n = 29) administered the challenge virus by intranasal instillation, 4 studies used a nebulizer to administer the challenge virus,^26^, ^27^, ^28^, ^29^ and 1 used a combination of intramuscular and intravenous injection.^24^ The time between the last vaccination and challenge also varied from 2 weeks to 1 year. Additional demographic information of all studies is provided in Table S1. The included studies generally involved small numbers of animals (<20 horses total) and often had wide (>5 years) age ranges. Breeds included Shetland ponies, Welsh Mountain pony, Thoroughbred, Standardbred, Quarter horse, Haflinger, and Iceland Ponies. Multiple studies failed to report the breeds used in their studies.

**TABLE 2:** Key characteristics of experimental studies.

Study	Vaccine(s)	Study design	Subjects	Controls	Challenge strain	Time from last vaccination (wk)	Comments
Bannai et al^41^	Equine Rhinopneumonitis Vaccine	Nonrandomized, controlled study	10	5	10‐I224	4	1‐ to 2‐year‐olds
Breathnach et al^16^	Army 183; Rhinomune; Pneumabort‐K; Rhinomune/Pneumabort‐K	Partially randomized, controlled study	16	2	Army 183	3‐10	Adults
Bürki et al^39^	Prevacinol; Pneumabort‐K	Nonrandomized, noncontrolled study	18	2	Piber 178/83	3‐16	Yearlings, adults, and pregnant mares
Burrows et al^26^	Pneumabort‐K	Nonrandomized, controlled study	35	20	3551/80	4‐8	1‐ to 2‐year‐olds and pregnant mares
Cornick et al^35^	TK‐EHV‐1 (H6b mutant)	Nonrandomized, placebo‐controlled study	8	4	Army 183	6.4	Foals
Dolby et al^47^	EHV‐1, strain V592	Nonrandomized, controlled study	5	3	V592	6	Yearlings
Goehring et al^32^	Rhinomune; Pneumabort‐K	Randomized, blinded, controlled study	16	8	Findlay OH03	3.4	Yearlings
Goodman et al^27^	Flu‐vac Innovator 6; Rhinomune	Randomized, blinded, controlled study	10	5	Findlay OH03	4.1	Adults
Hannant et al^28^	Immune stimulating complex vaccine	Randomized, controlled study	9	6	V592	3	Yearlings
Heldens et al^23^	Duvaxyn EHV1,4	Randomized, controlled study	15	9	121 412 or Ab4	2‐4	Foals and pregnant mares
Kydd et al^22^	Duvaxyn EHV‐1/4	Randomized, controlled study	5	9	AB4	4	Pregnant mares
Kydd et al^34^	EHV‐1 strain RacHΔgM	Nonrandomized, blinded, controlled study	6	6	Ab4	4	Foals
Matsumura (1996) ‐ Supporting Information, Item 4	EHV1 strain KyA	Nonrandomized, controlled study	4	2	89c25	4	Foals
Minke et al^30^	ALVAC‐EHV with or without carbopol; ALVAC‐EIV with carbopol	Randomized, placebo‐controlled	15	5	Ab4	3	1‐ to 2‐year‐olds
Minke et al^30^	gB/gC/gD plasmids with or without three different adjuvants	Randomized, placebo‐controlled	20	5	Ab4	2	1‐ to 2‐year‐olds
Mitchell et al^24^	Rhinomune	Nonrandomized, controlled study	4	2	Unknown	2	Unknown
Mohd‐Azmi et al^40^	AB4p L particles	Nonrandomized, controlled study	3	2	Ab4	4	Foals
Mumford et al^42^	Pneumabort‐K	Nonrandomized, controlled study	30	13	R500	12	Foals
Paillot et al^36^	vP1014 and carbomer‐PD adjuvant	Nonrandomized, placebo‐controlled study	3	1	Ab4	3‐12	1‐ to 2‐year‐olds
Patel et al^43^	EHV‐1 strain M8, C147	Nonrandomized, controlled study	11	6	Ab4	17‐26	Pregnant mares
Patel et al^48^	EHV‐1, Strain C147	Nonrandomized, controlled study	8	8	Ab4	6	1‐ to 2‐year‐olds
Patel et al^46^	EHV‐1 Strain C147	Nonrandomized, controlled study	8	6	Ab4	8	Foals
Perkins et al^29^	EHV‐1 Strain Ab4 or Ab4ΔORF1/71	Randomized, blinded, controlled study	10	5	Ab4	26	2‐year‐olds
Purdy et al^25^	Rhinoquin	Nonrandomized, controlled study	34	12	Unknown	5.7‐52	Adults and pregnant mares
Purdy et al^45^	Rhinoquin	Nonrandomized, controlled study	64	20	Unknown	11‐52	Foals
Schnabel et al^33^	EHV1 strain Ab4 or Ab4ΔORF2	Randomized, blinded, controlled study	16	8	Ab4	39	Adults
Soboll et al (2006) ‐ Supporting Information, Item 4	gB/gC/gD or IE/UL5 plasmids	Nonrandomized, controlled study	10	5	Army 183	4	Yearlings
Soboll et al^44^	rMVA‐IE	Nonrandomized, controlled study	21	5	Army 183	8	Adults with different genotypes
Thomson et al^37^	Rac‐H and various adjuvants	Nonrandomized, placebo‐controlled study	27	17	RAC‐H	6	Foals to 2‐year‐olds
Tsujimura et al^31^	ΔgE EHV‐1 strain	Nonrandomized, placebo‐controlled study	6	3	89C25p	4	Foals
Van de Walle (2009) ‐ see Supplementary, Item 4	rNY03ΔIR6/1gp2S	Nonrandomized, placebo‐controlled study	8	4	Findlay OH03	5	Adults
Wagner et al^49^	Streptavidin‐conjugated gC/IL‐4 fusion protein (Sav‐gC/IL‐4) ± IgE	Nonrandomized, controlled study	10	5	NY03	30	Newborns

*Note*: Total number of subjects and controls are provided.

Abbreviations: ALVAC‐EHV, recombinant canarypox virus (vCP132) expressing the gB, gC and gD glycoproteins of the Kentucky strain of EHV‐ 1; ALVAC‐EIV, recombinant canarypox virus (vCP1502) expressing the hemagglutinin of influenza A/eq/Prague/56 (H7N7); IE/UL5, immediate early (IE) and early proteins (UL5) of EHV‐1; rMVA‐IE, recombinant modified vaccinia Ankara vector expressing the IE gene; TK, thymidine kinase; vP1014, vaccinia‐based construct (NYVAC) that codes for an immediate early gene (gene 64) of EHV‐1.

Only 1 study^27^ was randomized, blinded, and placebo‐controlled. Two other studies^30^, ^31^ were unblinded, randomized, and placebo‐controlled. Three studies^29^, ^32^, ^33^ were randomized, blinded, and controlled (in controlled studies the controls were unvaccinated). Four studies were partially^16^ or fully randomized and controlled.^22^, ^23^, ^28^ One study^34^ was nonrandomized, blinded, and controlled. Five studies^31^, ^35^, ^36^, ^37^, ^38^ were nonrandomized and placebo‐controlled. One study^39^ was nonrandomized and lacked a control that underwent a challenge exposure. This study was excluded from all subsequent analyses. The remaining studies (n = 16) were categorized as nonrandomized, controlled studies. Eight classes of vaccines were used including commercially available MLVs (n = 8 studies), inactivated vaccines (n = 8 studies), and one study^16^ that used a combination of commercially available MLV and an inactivated vaccine. Experimental vaccines included MLVs (n = 5 studies), deletion mutants (n = 6 studies), DNA vaccines (n = 2 studies), inactivated vaccines (n = 5), and recombinant vaccines (n = 3). Many of the studies were underpowered (Tables S4‐S6). Two studies^36^, ^40^ relied on single animals for trials with a vaccine candidate. With few exceptions, studies generally used fewer than 5 horses per treatment group and often reported statistically insignificant findings. Abortion and neurologic signs consistent with EHM were rare events even in unvaccinated controls exposed to neuropathogenic or abortigenic strains of EHV‐1. Only 1 study^31^ provided dose‐response data that was limited to 2 different doses (10^5^ or 10^6^ PFU) of a candidate deletion mutant vaccine. Two studies^35^, ^41^ compared the efficacy of a vaccine candidate using different routes of administration (e.g., intranasal vs intramuscular). Three studies^25^, ^36^, ^42^ compared the efficacy of prime‐boost administration of candidate vaccines. Several studies^22^, ^23^, ^25^, ^39^, ^43^ had 1 or more experimental arms evaluating the efficacy of candidate vaccines in pregnant mares). With the exception of one study^43^ that used an experimental MLV, all other studies that used pregnant mares evaluated commercially available vaccines.

Most studies mentioned that rectal temperature was measured; however, 3 studies^24^, ^39^, ^44^ did not report rectal temperature measurements. A total of 24 studies reported incidence data for pyrexia that was used to calculate relative risk and perform a meta‐analysis.

Clinical signs related to respiratory tract involvement (e.g., nasal discharge, cough, changes in respiratory rate) were not evaluated in 4 studies.^36^, ^38^, ^40^, ^45^ Four other studies did not report data concerning this outcome.^22^, ^24^, ^35^, ^44^ The remaining 23 studies provided data regarding this outcome; however, in many cases, analysis of clinical scores or duration of clinical signs were used rather than a reporting of incidence data. In addition, the types of signs (e.g., nasal or ocular discharge, conjunctivitis, cough, respiratory rate) varied between studies, which precluded pooling of the limited incidence data for a meta‐analysis. Statistically significant results for this outcome were largely lacking with only 8/23 studies reporting significant findings. Decreased incidence or severity of ocular and/or nasal discharge was seen in horses vaccinated with commercially available MLV or killed vaccines and challenged with either Army183^16^ or Findlay OH03.^32^ One study reported decreased respiratory signs in foals following vaccination with a commercial MLV and challenge with an unidentified EHV‐1 isolated from an aborted foal.^45^ Vaccination with a gE deletion mutant (EHV‐1ΔgE) decreased the severity of respiratory signs following challenge with EHV‐1 strain 89C25p.^31^ Several studies reported decreased incidence or severity of ocular and/or nasal discharge in horses vaccinated with different experimental MLV or EHV‐1 deletion mutants as vaccine candidates followed by challenge with AB4 or AB4/8.^29^, ^33^, ^34^, ^46^

The presence of lymph node swelling went unevaluated or unreported in 12 studies.^22^, ^24^, ^25^, ^28^, ^32^, ^35^, ^36^, ^37^, ^38^, ^44^, ^45^, ^47^ Nine studies using either a commercial or experimental MLV, an experimental deletion mutant vaccine, or a commercial killed vaccine reported a significant decrease in either the incidence, severity, or duration of lymphadenopathy.^23^, ^29^, ^33^, ^34^, ^41^, ^42^, ^43^, ^46^, ^48^ The effect of vaccination on white blood cell counts was evaluated in 3 studies all of which reported a vaccine benefit.^25^, ^35^, ^45^ No studies were found regarding vaccination and orchitis or other male reproductive effects.

Abortion or neonatal loss was evaluated in 6 studies^22^, ^24^, ^25^, ^26^, ^39^, ^43^ and data from 5 studies were included in the meta‐analysis. Neurologic effects, including changes in gait and mental status, were evaluated in 9 studies^22^, ^24^, ^27^, ^29^, ^32^, ^33^, ^38^, ^44^, ^49^ and data from 8 studies were included in the meta‐analysis. Viremia was not evaluated in 3 studies.^24^, ^37^, ^42^ Nasal shedding of EHV‐1 was not reported in 1 study.^24^ Viremia and nasal shedding were evaluated in all other vaccine trials. A total of 24 studies reported incidence data for viremia and nasal shedding that were used to calculate relative risks and perform separate meta‐analyses.

### 3.3 Risk of bias in individual studies

Summary risk‐of‐bias assessments for the included studies are presented in Supporting Information Item 5 and Figure 1. Critical risk of bias domains included groups being similar at baseline, blinding for certain clinical outcomes (e.g., neurologic evaluations, evaluation of lymphadenopathy, scoring of clinical signs), incomplete outcome data, selective reporting, and other sources of bias including concerns about statistical analyses. Incomplete reporting of methods frequently led to an unknown risk of bias for several domains including concealment of animals to experimental groups, random housing of animals, blinding of investigators and outcome assessors, and other problems—most commonly an incomplete description of the possible role of funders or a lack of statistical analyses. Most studies assessed all animals in the study for all relevant outcomes; thus, incomplete or selective outcome reporting was not identified as a concern in nearly all studies. Methods used to evaluate viremia and nasal shedding included plaque assays and qPCR. These methods have different sensitivities that could bias results. High risk of bias was noted for one or more individual domains in 12 studies. Details concerning high risk of bias items are provided in Supporting Information Item 5.

### 3.4 Summary of findings

Extracted study results are presented in Supplemental Data Table S3.

#### 3.4.1 Pyrexia

**FIGURE 2:**
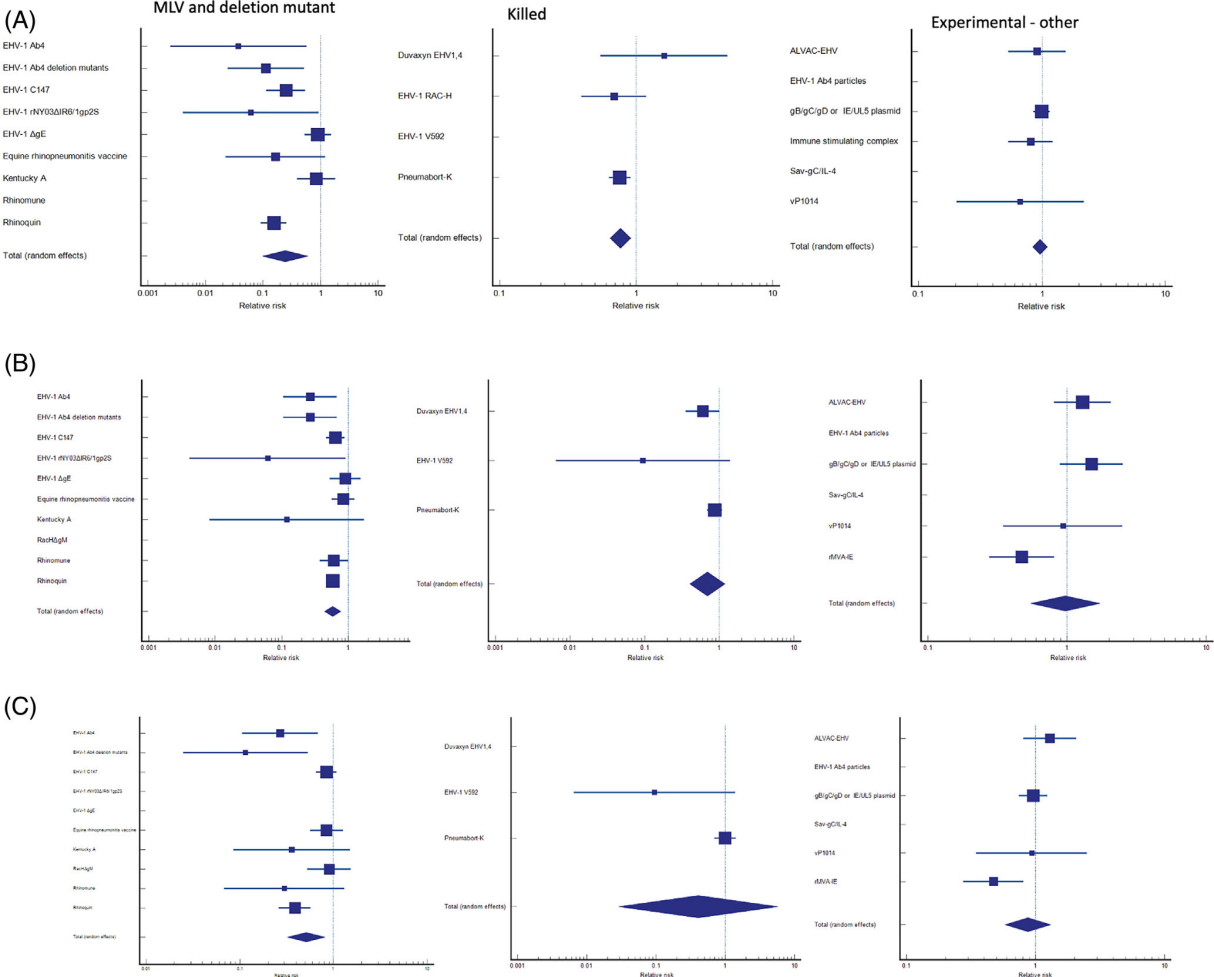
Forest plot analysis of the overall incidence of main outcomes of interest in EHV‐1 infected horses. (A) Early phase pyrexia; (B) cell‐associated viremia; and (C) nasal shedding.

Figure S2 shows the results of a global (all vaccines) meta‐analysis of the incidence of pyrexia in vaccinated and unvaccinated horses in the first 3 days after EHV‐1 challenge. Overall, 9/36 (25%) trials reported that vaccination was significantly associated with a reduced frequency of pyrexia in EHV‐1 infected horses. A pooled estimate of the relative risk of pyrexia following vaccination of 0.468 (95% confidence interval [CI]: 0.318‐0.688; *z* = −3.854; *P* < .001) was found. Both the Egger's test and the Begg's test revealed a significant (*P* < .0001) publication bias. The between‐trial heterogeneity was severe when using a random effects model (78.81%, 95% CI: 69.04‐85.49; *P* < .0001). The analysis was subsequently broken down by the different vaccine types used in the studies to potentially account for the biological heterogeneity present in the trials (Figure 2, Table 3). The following 3 broad categories were used: MLV vaccines (including deletion mutants); inactivated virus products, and other experimental vaccines. Heterogeneity remained severe for the MLV vaccines (Table 4). Thus, the beneficial response seen with the MLV vaccines needs to be interpreted with caution. Killed virus products also significantly decreased the incidence of viremia in EHV‐1‐infected horses. Publication bias was absent when vaccine type was evaluated in these subanalyses.

**TABLE 3:** Relative risk of main outcomes of interest following vaccination.

				Relative risk				*I* ^2^			Publication bias	
Outcomes	Type of vaccine	N	Relative risk	Significance	Lower limit	Upper limit	*I* ^2^ (%)	Significance	Lower limit	Upper limit	Egger's	Begg's
Pyrexia	MLV and deletion mutant	8	**0.24**	***P* = .002**	0.10	0.60	**84.9**	***P* < .0001**	72.2	91.9	No	No
	Killed	3	**0.77**	***P* = .003**	0.64	0.91	0	*P* = .37	0	96.6	No	No
	Experimental—other	4	0.96	*P* = .6	0.84	1.11	0	*P* = .59	0	79.8	No	No
Abortion	MLV and deletion mutant	3	0.33	*P* = .23	0.05	2.00	52.9	*P* = .12	0	86.5	No	No
	Killed	2	**0.40**	***P* = .01**	0.20	0.81	0	*P* = .46	0	0	**Yes**	No
Neurologic	MLV and deletion mutant	4	0.35	*P* = .16	0.09	1.22	0	*P* = .6	0	79.0	No	No
	Killed	2	0.88	*P* = .79	0.34	2.28	0	*P* = .39	0	0	**Yes**	No
	Experimental—other	2^a^	0.34	*P* = .59	0.01	16.3	NA	NA	NA	NA	NA	NA
Viremia	MLV and deletion mutant	9	**0.58**	***P* < .001**	0.44	0.77	**56.7**	***P* = .02**	8.9	79.4	No	No
	Killed	3	0.69	*P* = .18	0.40	1.18	59.4	*P* = .09	0	88.4	No	No
	Experimental—other	4	0.97	*P* = .92	0.55	1.71	**73.4**	***P* = .01**	25.1	90.5	No	No
Nasal shedding	MLV and deletion mutant	8	**0.51**	***P* = .004**	0.32	0.81	**78.5**	***P* < .001**	57.7	89.0	No	No
	Killed	2	0.41	*P* = .51	0.03	5.72	**75.3**	***P* = .04**	0	94.4	**Yes**	No
	Experimental—other	4	0.87	*P* = .51	0.58	1.31	**63.1**	***P* = .04**	0	87.6	No	No

*Note*: N represents total number of vaccine candidates in each subclass that contributed to the meta‐analysis.

^a^ Estimated relative risk based on two studies (Soboll et al^44^ and Wagner et al^49^).

**TABLE 4:** Summary of findings.

Outcome	Type of vaccine	Number of studies^a^	Relative effect (95% CI)	Quality of the evidence (GRADE)	Conclusion
Pyrexia	MLV and deletion mutant	16 (12)	RR 0.28 (0.15 to 0.55)	Low	Possible benefit; however additional research is needed.
	Killed	9 (4)	RR 0.78 (0.45 to 1.34)	Low	No benefit identified and additional research is needed.
	Experimental—other	6 (4)	RR 0.93 (0.78 to 1.11)	Low	No benefit identified and additional research is needed.
Abortion	MLV and deletion mutant	3 (2)	RR 0.33 (0.05 to 2.00)	Very low	No benefit identified. We are uncertain about the estimate.
	Killed	2 (2)	RR 0.40 (0.20 to 0.81)	Low	Possible benefit; however additional research is needed
Neurologic signs	MLV and deletion mutant	6 (4)	RR 0.35 (0.09 to 1.22)	Very low	No benefit identified. We are uncertain about the estimate.
	Killed	2 (2)	RR 0.88 (0.34 to 2.28)	Low	No benefit identified and additional research is needed.
	Experimental—other	2 (0)	RR ~0.34 (~0.01 to 16.3)	Low	No benefit identified and additional research is needed.
Viremia	MLV and deletion mutant	16 (13)	RR 0.60 (0.46 to 0.79)	Low	Possible benefit; however additional research is needed
	Killed	7 (5)	RR 0.47 (0.19 to 1.20)	Low	No benefit identified and additional research is needed.
	Experimental—other	7 (4)	RR 0.91 (0.49 to 1.68)	Low	No benefit identified and additional research is needed.
Nasal shedding	MLV and deletion mutant	16 (10)	RR 0.51 (0.31 to 0.84)	Low	Possible benefit; however additional research is needed.
	Killed	9 (3)	RR 0.90 (0.67 to 1.23)	Low	No benefit identified and additional research is needed.
	Experimental—other	7 (3)	RR 0.87 (0.72 to 1.04)	Low	No benefit identified and additional research is needed.

*Note*: Relative effect estimates based on exploratory meta‐analyses performed on pooled results from individual studies.

^a^ Values in parentheses represent the number of studies that contributed to the exploratory meta‐analyses and quantitative estimation of the relative risk.

#### 3.4.2 Abortion

**FIGURE 3:**
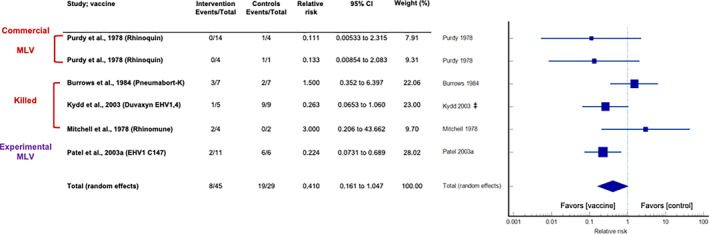
Forest plot analysis of the overall incidence of abortion in EHV‐1 infected horses.

Figure 3 shows the results of a global (all vaccines) meta‐analysis of the incidence of abortion in vaccinated and unvaccinated horses following EHV‐1 challenge. Overall, only 1/6 (17%) vaccine trials reported that vaccination was significantly associated with a reduced frequency of abortion in EHV‐1‐infected horses. A pooled estimate of the relative risk of abortion following vaccination of 0.410 (95% CI: 0.161 to 1.047; *z* = −1.865; *P* = .06) was found, which suggested that vaccination offered no benefit in the prevention of abortion. Neither test of publication bias was significant. The between‐trial heterogeneity was insignificant when using a random effects model (37.8%, 95% CI: 0‐75.3; *P* = .15). Table 3 provides the results of the exploratory meta‐analyses performed on the vaccine subclasses.

#### 3.4.3 Neurologic signs

**FIGURE 4:**
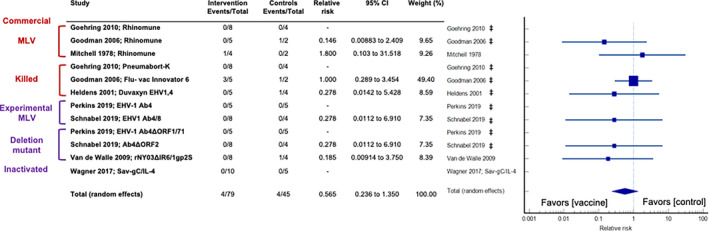
Forest plot analysis of the overall incidence of neurologic signs in EHV‐1 infected horses.

Figure 4 shows the results of a global (all vaccines) meta‐analysis of the incidence of neurologic signs in vaccinated and unvaccinated horses following EHV‐1 challenge. Overall, none of the vaccine trials (0/10) reported that vaccination was significantly associated with a reduced frequency of neurologic signs in EHV‐1‐infected horses. A pooled estimate of the relative risk of ataxia and other neurologic signs following vaccination of 0.964 (95% CI: 0.841‐1.105; *z* = −1.285; *P* = .2) was found, which indicated that vaccines had no effect on the incidence of neurologic signs. Neither the Egger's test nor the Begg's test revealed a significant publication bias. The between‐trial heterogeneity was insignificant when using a random effects model (0%, 95% CI: 0‐54.1; *P* = .71). Table 3 provides the results of the exploratory meta‐analyses performed on the vaccine subclasses.

#### 3.4.4 Viremia

Figure S3 shows the results of a global (all vaccines) meta‐analysis of the incidence of viremia in vaccinated and unvaccinated horses following EHV‐1 challenge. Overall, 4/38 (10.5%) trials reported that vaccination was significantly associated with a reduced frequency of cell‐associated viremia in EHV‐1‐infected horses. A pooled estimate of the relative risk of viremia following vaccination of 0.590 (95% CI: 0.444‐0.783; *z* = −3.652; *P* < .001) was found. Both the Egger's test (*P* < .0001) and the Begg's test (*P* = .001) revealed a significant publication bias. The between‐trial heterogeneity was severe when using a random effects model (67.65%, 95% CI: 52.12‐78.15; *P* < .0001). The analysis was subsequently broken down into different vaccine types to potentially account for the biologic heterogeneity of the preparations used in the trials (Figure 2, Table 3). Vaccine subtypes were broken down into the following 3 broad categories: MLV vaccines (including deletion mutants); killed virus products, and other experimental vaccines. Heterogeneity remained severe for both the MLV vaccines and other experimental vaccines (Table 3). Thus, the beneficial response seen with the MLV vaccines needs to be interpreted with caution. Neither killed virus products nor other types of experimental vaccines significantly reduced the incidence or magnitude of viremia in EHV‐1‐infected horses. Publication bias was insignificant when vaccine type was evaluated in the sub‐analyses.

#### 3.4.5 Nasal shedding

Figure S4 shows the results of a global (all vaccines) meta‐analysis of the incidence of nasal virus shedding in vaccinated and unvaccinated horses following EHV‐1 challenge infection. Overall, 2/32 (6.3%) trials reported that vaccination was significantly associated with a reduced frequency of nasal shedding in EHV‐1 infected horses. A pooled estimate of the relative risk of nasal shedding following vaccination of 0.661 (95% CI: 0.512 to 0.855; *z* = −3.158; *P* = .002) was found. Both the Egger's test (*P* < .0001) and the Begg's test (*P* = .002) revealed a significant publication bias. The between‐trial heterogeneity was severe when using a random effects model (67.29%, 95% CI: 49.49‐78.82; *P* < .0001). To try and address biologic heterogeneity present in the trials the analysis was subsequently broken down into the different vaccine types indicated above MLV (includes deletion mutants); killed virus products, and other experimental vaccines. Heterogeneity remained severe for all three vaccine subtypes (Figure 2, Table 3). Our results suggest that MLV vaccines may decrease the incidence of nasal shedding in horses; however, the high degree of heterogeneity in these studies indicates that these results should be interpreted with caution. Neither killed virus products nor other types of experimental vaccines significantly altered the incidence of nasal shedding in EHV‐1‐infected horses. Caution in interpreting the results of these subanalyses by vaccine type remains. Publication bias was insignificant when vaccine type was evaluated in the subanalyses.

### 3.5 Rating the overall quality of evidence

Overall evidence from the experimental studies was evaluated using GRADE. One study^39^ used sentinel animals as a comparator (vs. unvaccinated or placebo controls). All other experimental studies met each of the individual rating factors and received an initial GRADE score of 4. The level of evidence for experimental studies was subsequently downgraded because of a lack of consistency due to considerable heterogeneity and because of imprecision due to underpowered studies. Additional downgrades for the lack of blinding or other quality concerns also occurred with some outcomes (abortion, neurologic effects). Publication bias did not result in downgrading of the evidence (Table 3). Overall, low to very low quality of evidence existed for evaluating whether vaccination reduces the risk of pyrexia, abortion, neurologic effects, viremia, or nasal shedding. The GRADE results are presented in Tables S4 to S6. The summary of findings for the main outcomes is provided in Table 4.

## 4 DISCUSSION

This systematic review provides the result of our search of electronic and print resources of peer‐reviewed publications in any language and without restriction to publication date. We did not include conference proceedings, technical reports, and other gray literature in this review and this potential publication bias needs to be considered when evaluating our study. We found that reporting of the methodological features of studies was often incomplete, making evaluation of risk of bias within and across studies difficult. Incomplete reporting of methodological details in experimental animal studies and animal‐centric systematic reviews have been noted by others.^50^, ^51^, ^52^ Increased adherence of study authors to reporting guidelines^53^ remains urgent in the veterinary literature.

Our systematic review found a diverse range of experiments that varied by age of animals, reproductive status, breed of horses, vaccine dosages, strain of EHV‐1 used in challenges, time from vaccination to challenge, and outcomes of interest, among other factors. This heterogeneity in study designs and outcomes is reflected in our meta‐analyses performed on all vaccines (global analysis of all vaccines), and the subsequent meta‐analyses that evaluated broad classes of vaccines. When considered collectively, MLV vaccines were shown to reduce the incidence of pyrexia (RR = 0.243 [95% CI: 0.099‐0.595]), viremia (RR = 0.584 [95% CI: 0.442‐0.773]), and nasal shedding (RR = 0.511 [95% CI: 0.323‐0.808]). Severe heterogeneity was consistently present in these sub‐analyses with MLV vaccines. Inactivated viral vaccines reduced the incidence of pyrexia (RR = 0.766 [95% CI: 0.644‐0.911]) but had a limited effect on viremia or nasal shedding in EHV‐1‐infected horses. This finding represents data drawn from only 2 to 3 vaccines or vaccine candidates. Other experimental vaccines were ineffective with respect to reducing the incidence of pyrexia, viremia, or nasal shedding. However, this conclusion is based on a small sample of 4 vaccine candidates. The paucity of high‐quality studies prevented further attempts to evaluate the efficacy of any individual vaccine for our main outcomes of interest. Moreover, the database was inadequate to evaluate whether vaccine efficacy varied with EHV‐1 challenge strain, timing of challenge infection, or other factors that could be of clinical importance.

Underpowered studies were prevalent for all clinical outcomes of interest. Those studies evaluating vaccine efficacy for either abortion or EHM were consistently underpowered leading to null findings in nearly all studies. The incidence of abortion or ataxia and other clinical signs associated with EHM in unvaccinated controls in these studies was often 0% and when present was generally <10% to 20%. Meta‐analysis of these underpowered studies failed to show that vaccination would prevent or reduce the incidence of either abortion or EHM in horses. Underpowered studies led to a consistent concern about precision and downgrading in the quality of the evidence for all vaccine subtypes. Our systematic review clearly identifies the need for rigorous randomized and blinded studies to evaluate vaccine efficacy with EHV‐1.

None of the previous systematic review frameworks (e.g., GRADE and the Cochrane Collaboration) address approaches for considering animal studies. For this reason, we adopted the OHAT GRADE framework that uses different criteria for determining a body of literature as the starting point in the GRADE process. Most literature available for individual outcomes was then downgraded by 2 to 3 levels, resulting in final confidence ratings of low to very low. This observation is consistent with some other reviews of the human medical literature.^54^, ^55^ Factors that contribute to these lower‐quality studies included risk of bias and imprecision. Similar downgrades were applied in the present study for concerns regarding precision, especially as a consequence of underpowered studies for detecting rare events including abortion and neurologic effects. Effect estimates for other outcomes including pyrexia, viremia, and nasal shedding often had broad confidence intervals indicating that the effect estimates were imprecise.

Since the conduct of the literature searches underlying our study, one notable publication was published, which was a systematic review of the efficacy of vaccination against EHV‐1 infection.^56^ The review was restricted to RCTs involving experimental challenge infections, and selected and analyzed 8 studies, all of which were among the 35 studies described here. Primary outcomes included in this publication are similar to ours and include respiratory signs, abortion, or neurological sequelae seen after challenge infection of vaccinated compared with unvaccinated horses. The previously conducted systematic review also considered secondary outcomes including the extent and duration of virus shedding and viremia, and the results and conclusions were similar to those presented here. For example, our review as well as that performed earlier by Marenzoni et al^54^ revealed poor reporting quality of the selected studies. Their meta‐analysis using a random effects model failed to demonstrate that vaccines reduced the number of vaccinated horses with at least one clinical sign following virus challenge infection (pooled RR 0.97, 95% CI 0.86‐1.10, *P* = .62) with lower heterogeneity (*I* ^2^ = 19%), which contrasts with the results reported here. The observed differences may be a result of the different models used for analysis, the differences and numbers of studies included, and/or the assessment criteria that we used in our study.^54^

In conclusion, our meta‐analysis overall provided a sobering view of many experimental studies testing EHV‐1 vaccines in horses over more than 6 decades. We argue that there is a pressing need for randomized clinical trials and higher‐powered studies to inform decisions regarding the use of EHV‐1 vaccination for the prevention of nasal shedding, viremia, abortion or equine herpesvirus myeloencephalopathy in domesticated horses.

## CONFLICT OF INTEREST DECLARATION

Authors declare no conflict of interest.

## OFF‐LABEL ANTIMICROBIAL DECLARATION

Authors declare no off‐label use of antimicrobials.

## INSTITUTIONAL ANIMAL CARE AND USE COMMITTEE (IACUC) OR OTHER APPROVAL DECLARATION

Authors declare no IACUC or other approval was needed.

## HUMAN ETHICS APPROVAL DECLARATION

Authors declare human ethics approval was not needed for this study.

## Supporting information


**Data S1.** Supporting Information.
