# Supplementary material for: Vaccination for the prevention of equine herpesvirus‐1 disease in domesticated horses: A systematic review and meta‐analysis
Source: J Vet Intern Med. 2023 Nov 6;38(3):1858–71. doi: 10.1111/jvim.16895 (PMC11099739; doi:10.1111/jvim.16895)
Supplement: Supplementary file 1 — Data S1. Supporting Information. [file JVIM-38-1858-s001.pdf]

Supplementary Materials

Item 1: Study Protocol

Title: Vaccinations for the Prevention of Equine Herpesvirus-1 (EHV-1) Disease in Domesticated Horses: A Systematic Review

Research question: Does vaccination protect against EHV-1 infection and disease?

Problem formulation: The review question was developed and refined through a series of problem formulation steps including preliminary literature searches.

Searches: The review team will consider using existing systematic reviews to address or help to address its research question. English-language systematic reviews conducted within the last 5 years will be sought using searches in PubMed, PROSPERO (CRD), and CAMARADES. A search for bibliographic references will be performed with the assistance of an experienced librarian through MEDLINE (via PubMed), LILACS (via Virtual Health Library), Cochrane Library (via Virtual Health Library) and EMBASE to locate randomized controlled trials and other studies. The search will be limited to domesticated horses and performed without sex, age, breed, or language restrictions. Only peer-reviewed publications will be considered. The search strategies will include the search for descriptors or words in the text related to the disease and the type of intervention. The search will be developed with input from a librarian with expertise in the conduct of systematic reviews.

Types of studies to be included: Randomized controlled trials and observational studies.

Condition or domain being studied: Equine herpesvirus-1 (EHV-1) is a highly prevalent Alphaherpesviridae virus that infects horses worldwide. This respiratory virus is transmitted via direct horse-to-horse contact with contaminated nasal secretion as well as indirectly from contact with contaminated aborted fetuses, placenta, and fomites. This virus is associated with equine herpesvirus-1 myeloencephalopathy (EHM), respiratory disease, abortion, and neonatal death among other outcomes. Treatment is primarily supportive, and usually includes anti-inflammatories and fluids to maintain hydration. This review considers vaccinations for the prevention of EHV-1 disease in horses.

Disease definition: clinical outcomes that result from EHV-1 infection include one or more of the following:

- Rhinopneumonitis: pyrexia with respiratory signs, including oculo-nasal discharge, elevated respiratory rate, cough, lethargy
- Abortion in the third trimester
- Equine Herpes Myeloencephalopathy
- Neonatal infection
- Ocular disease
- Male Reproductive tract infection - orchitis

PICO Statement

- **Population**: Domesticated equids without sex, age, or breed restrictions
- **Intervention:** EHV-1 vaccination without restriction of vaccine type (e.g., modified live) or vaccination protocol.
- **Comparator:** Equids experimentally infected or naturally exposed to EHV-1 infection, after receiving placebo, or other vaccines.
- **Outcome:** All clinical outcomes that reflect symptomatic EHV-1 infection or viral infection. Presence and degree of viral infection. Endpoints related to vaccine efficacy (relative reduction in EHV-1) risk after vaccination, effectiveness (reduction in odds of EHV-1) associated with vaccination in an observational study, are relevant outcomes.

Inclusion and Exclusion Criteria

Inclusion:

- Domesticated equids without sex, age, breed, or immunological status restriction
- Vaccination trials that evaluated the efficacy of vaccines against EHV-1, experimental challenge or natural infection
- Studies that used a placebo or other vaccine.
- Study included clinical outcomes that reflect symptomatic EHV-1 infection.

- Endpoints related to vaccine efficacy: relative reduction in EHV-1 disease risk; reduction in odds of EHV-1 infection
- Studies will not be excluded based on year, language, or quality

Exclusion:

- Absence of an EHV-1 challenge trial of vaccine efficacy
- Absence of the selected clinical or virological outcomes
- Wrong species of virus
- Lack of concurrent control or comparator
- Wrong species (not equid)
- Purely descriptive observational studies
- No original data

Reason for exclusion for all studies will be recorded.

Review team: The review team will include Peggy Gross (Librarian), David Dorman, Paul Lunn, and Lutz Goehring. If a member of the review team was a coauthor of a study under review, that member will recuse himself or herself from the evaluation of the quality of that study. The review team will be responsible for performing all aspects of the review, including conducting the literature searches; applying inclusion/exclusion criteria to screen studies; extracting data; assessing risk of bias for included studies; and analyzing and synthesizing data. The roles and responsibilities of the team members will be documented throughout the protocol. Throughout the course of its work, the review team will also engage others as needed. The involvement of those individuals will be documented.

Study selection

The evaluation of titles, abstracts, and the full text will be independently performed by two reviewers; disagreements will be resolved by a third reviewer. A database management system (Covidence) will be used to manage and document these decisions. Reason for exclusion for all studies will be recorded. The evaluation of titles, abstracts, and the full text

will be independently performed by two reviewers; disagreements will be resolved by a third reviewer. All study authors will be excluded from evaluation of their work.

Data extraction: Data will be collected and recorded (i.e., extracted) from included studies by one member of the review team (DCD) and checked by a second member for completeness and accuracy. Any discrepancies in data extraction will be resolved through discussion. The extracted data will be used to summarize study designs and findings and/or to conduct statistical analyses.

Outcomes of interest: Will relate to the efficacy of the herpes vaccine in preventing symptomatic herpes infection. A standardized form will be developed for data extraction with fields referring to the characteristics of identification of the studies (including study author, country, study design [e.g., randomized trial: case-control: cohort: other]; subject age range (years); subjects (sex, age, breed, and number); follow-up time) and the observed outcomes (see below) including when observations were made. Data regarding the seropositivity of the individuals at all phases of the study will also be extracted.

Specific study endpoints to be extracted include:

- Challenge protocol:
  - Virus challenge used
  - Dose & Route of administration
- Clinical signs (Yes/No, duration in days, severity):
  - Fever (rectal temperature >38.6C),
  - Clinical score – based on some combination of nasal discharge, cough, tachypnea/dyspnea, anorexia, lethargy
  - Neurologic disease, for example: ataxia; weakness; and urinary incontinence.
- Clinical Pathology
  - Leukogram changes or blood changes indicative of inflammatory disease
  - CSF color, cytology and protein concentration
- Pathology

    - CNS pathology
    - URT pathology
    - Reproductive tract pathology
    - Airway pathology
    - Ocular pathology
- Virology (Yes/No, duration in days, quantitation):
    - Viral culture from nasal/naso-pharyngeal swabs and methods used
    - Viral DNA in nasal swab samples and method used
    - Viral culture from blood and methods used
    - Viral DNA in blood and method used
    - Viral DNA in tissues

Risk of bias evaluation: The risk of bias domains and questions for are based on established guidance for animal studies. [1] The SYRCLE risk of bias tool includes a common set of questions that are answered based on the specific details of individual studies to develop risk of bias ratings (using the following three options: low risk of bias; unknown risk of bias; or high risk of bias). Information or study procedures that were not reported are assumed not to have been conducted, resulting in an assessment of "unknown" risk of bias. Studies will be independently assessed by two assessors (to be determined) who answer all applicable risk of bias questions with one of three options following prespecified criteria. Risk of bias will be assessed at the outcome level. After assessors have independently made risk of bias determinations for a study across all risk of bias questions, the two assessors will compare their results to identify discrepancies and attempt to resolve them. Any remaining discrepancies will be considered and resolved with the review team. The final risk of bias rating for each question will be recorded along with a statement of the basis for that rating. All risk of bias assessments will be recorded using Covidence. The following domains will be assessed:

- Random sequence generation

- Allocation sequence
- Blinding of participants and personnel
- Blinding of outcome assessment
- Incomplete outcome data
- Selective reporting
- Other bias

Strategy of data synthesis: A narrative synthesis (e.g., study design, year of publication, subject baseline demographics, sample size, country where study was conducted, interventions, and the results from each study) will be performed for each intervention. If we identify enough studies with adequate homogeneity, a meta-analysis will also be considered.
Confidence rating: Assessment of the body of evidence: The quality of evidence for each therapy will be evaluated using the GRADE system for rating the confidence in the body of evidence. [2]; [3] In brief, available studies on a particular outcome and vaccine will be initially grouped by key study-design features, and each grouping of studies is given an initial confidence rating by those features. The initial rating is downgraded for factors that decrease confidence in the results, including risk of bias, unexplained inconsistency, indirectness or lack of applicability, imprecision, or publication bias. The initial rating is upgraded for factors that increase confidence in the results, including large magnitude of effect, dose response, consistency across study designs/populations/animal models or species, consideration of residual confounding, other factors that increase confidence in the association or effect (e.g., particularly rare outcomes). Confidence ratings are independently assessed by members of the review team, and discrepancies will be resolved by consensus as needed. Confidence ratings will be summarized in evidence profile tables.

Protocol developed: 23 DEC 19

Protocol Amendments

Risk of bias:

Training manual developed: 19 JUN 2020

Review team:

The following individuals were added to the review team:

- Kelsie Dougherty 28 JUL 2020
    - Roles: Data extraction, risk of bias assessment
- Claire Neinast 4 AUG 2020
    - Roles: Data extraction, risk of bias assessment
- Kerry O'Donnell 10 MAY 2021
    - Roles: Data extraction
- Irene Nazarro 10 JUN 2021
    - Roles: Data extraction

Exclusion criteria:

Only peer-reviewed publications will be considered. Added by working group consensus: 20 AUG 2020

Item 2: Search Strategy

PubMed

Concept 1: EHV1

"Herpesvirus 1, Equid"[Mesh] OR “equine herpesvirus 1”[tw] OR “equine herpes virus 1”[tw] OR “Equine abortion Virus”[tw] OR “Equine abortion Viruses”[tw] OR “EHV 1”[tw] OR EHV1[tw] OR “equid herpesvirus 1”[tw] OR "equid herpesvirus type 1"[tw] OR "Equine herpesvirus myeloencephalopathy"[tw] OR “Equine herpes myeloencephalopathy”[tw] OR EHM[tw] OR "equine herpesvirus type 1"[tw] OR "equine herpes virus type 1"[tw] OR "alphaherpesvirus"[tw]

Concept 2: Horses

horses[mesh] OR horse[tw] OR horses[tw] OR equid*[tw] OR equine*[tw] OR equus[tw]

Concept 3: vaccines

"Vaccines"[Mesh] OR "Viral Vaccines"[Mesh] OR "Vaccination"[Mesh] OR "Vaccine"[tw] OR "Vaccines"[tw] OR "Vaccination"[tw] OR "Vaccinations"[tw] OR vaccinia[tw] OR "Immunization"[tw] OR "Immunize"[tw] OR "Immunized"[tw] OR "Immunization"[tw] OR "Immunizations"[tw] OR "Immunise"[tw] OR "Immunised"[tw] OR "Immunisation"[tw] OR "Immunisations"[tw] OR “modified live virus”[tw] OR inoculations[tw] OR inoculation[tw] OR “Immune response”[tw] OR Pneumabort-K[tw] OR "Pneumabort-K" [Supplementary Concept] OR Prodigy[tw] OR Rhinomune[tw] OR Calvenza[tw] OR "Adjuvants, Immunologic"[Mesh] OR Immunostimulants[tw] OR “Immunologic Adjuvant”[tw] OR “Immunological Adjuvant”[tw] OR “Immunoactivators”[tw] OR Immunoadjuvants[tw] OR “Immunologic Adjuvants”[tw] OR “Immunopotentiators”[tw] OR “Immunological Adjuvants”[tw]

Concept 3: article type

NOT comment[pt] OR editorial[pt] OR letter[pt] OR news[pt] OR “newspaper article”[pt] OR review[pt] OR “systematic review”[pt] OR “meta-analysis”[pt] OR “consensus development conference”[pt] OR guideline[pt] OR “practice guideline”[pt] OR bibliography[pt]

Web of Science

Concept 1:EHV1

TS=(“Equine abortion Viruses” OR “EHV 1” OR EHV1 OR “equid herpesvirus 1” OR "equid herpesvirus" OR "Equine herpesvirus myeloencephalopathy" OR “Equine herpes myeloencephalopathy” OR EHM OR "equine herpesvirus type 1" OR "equine herpes virus type 1" OR "alphaherpesvirus")

Concept 2: Horses

TS=(horse OR horses OR equid* OR equine* OR equus)

Concept 3: vaccines

TS=("Vaccine" OR "Vaccines" OR "Vaccination" OR "Vaccinations" OR vaccinia OR "Immunization" OR "Immunize" OR "Immunized" OR "Immunization" OR "Immunizations" OR "Immunise" OR "Immunised" OR "Immunisation" OR "Immunisations" OR “modified live virus” OR inoculations OR inoculation OR “Immune response” OR "Pneumabort-K" OR Prodigy OR Rhinomune OR Calvenza OR Immunostimulants OR “Immunologic Adjuvant” OR “Immunological Adjuvant” OR “Immunoactivators” OR Immunoadjuvants OR “Immunologic Adjuvants” OR “Immunopotentiators” OR “Immunological Adjuvants”)

Concept 4: article type

NOT comment[pt] OR editorial[pt] OR letter[pt] OR news[pt] OR “newspaper article”[pt] Review OR “meta-analysis” OR “consensus development conference” OR guideline OR “practice guideline” OR bibliography

Item 3: List of studies that were excluded based on a review of the full text and reason for exclusion.

**Study**	**Reason for exclusion**
Alkhleif O, Pellerin JL, Larrat M, Roux C, Maindron E, Richard L, Fiéni F, Bruyas JF. Systematic and regular vaccination against rhinopneumonitis of all horses in a breeding stud can decrease the risk of excretion of EHV1 in semen of healthy stallions. J Equine Vet Sci 2012;32(8):475-518.	Wrong outcomes
Anonymous. Tierarztl Prax Ausg G Grosstiere Nutztiere 2018; 46(1):65.	No original data
Bannai H, Mae N, Ode H, Nemoto M, Tsujimura K, Yamanaka T, Kondo T, Matsumura T. Successful control of winter pyrexias caused by equine herpesvirus type 1 in Japanese training centers by achieving high vaccination coverage. Clin Vaccine Immunol. 2014;21(8):1070-6.	Wrong study design
Becker W. [Preventative vaccination against EHV (equine herpesvirus) abortion]. Tierarztl Prax 1988;16(1):61-3.	Wrong study design
Bresgen C, Lämmer M, Wagner B, Osterrieder N, Damiani AM. Serological responses and clinical outcome after vaccination of mares and foals with equine herpesvirus type 1 and 4 (EHV-1 and EHV-4) vaccines. Vet Microbiol. 2012;160(1-2):9-16.	Unknown exposure history
Bresgen C, Lämmer M, Wagner B, Osterrieder N, Damiani AM. Serological responses and clinical outcome after vaccination of mares and foals with equine herpesvirus type 1 and 4 (EHV-1 and EHV-4) vaccines. Vet Microbiol. 2012;160(1-2):9-16.	Duplicate

Bryans JT, Allen GP. Application of a chemically inactivated, adjuvanted vaccine to control abortigenic infection of mares by equine herpesvirus I. Dev Biol Stand 1982; 52:493-8.	Wrong study design
Bryans JT. Serologic responses of pregnant thoroughbred mares to vaccination with an inactivated equine herpesvirus 1 vaccine. Am J Vet Res 1980;41(11):1743-6.	No challenge trial
Buchner HHF, Mostl K.Outbreak of an equine herpesvirus infection (EHV-1) in an university clinic Wiener Tierarztliche Monatsschrift 1998;85(3):87-93.	Wrong study design
Burrows R, Goodridge D, Denyer MS. Trials of an inactivated equid herpesvirus I vaccine: challenge with a subtype 1 virus. Vet Record 1984;114(15):369-374.	Duplicate
Codazza D, Socci A. [Field trials of a vaccine (Prevaccinol) against equine rhinopneumonitis.]. / Osservazioni sulla sperimentazione in campo di un vaccino contro la rinopneumonite equina. Folia Veterinaria Latina 1971; 1(3/4):829-836	Wrong study design
Damiani AM, de Vries M, Reimers G, Winkler S, Osterrieder N. A severe equine herpesvirus type 1 (EHV-1) abortion outbreak caused by a neuropathogenic strain at a breeding farm in northern Germany. Vet Microbiol. 2014;172(3-4):555-62.	Wrong study design
Dolby C, Hannant D, Mumford J. Response of ponies to adjuvanted EHV-1 whole virus vaccine and challenge with virus of the homologous strain. Br Vet J. 1995 Jan-Feb; 151(1):27-37.	Duplicate
Foote CE, Love DN, Gilkerson JR, Wellington JE, Whalley JM. EHV-1 and EHV-4 infection in vaccinated mares and their foals. Vet Immunol Immunopathol. 2006;111(1-2):41-6.	No challenge trial

Foote CE, Love DN, Gilkerson JR, Whalley JM. Detection of EHV-1 and EHV-4 DNA in unweaned Thoroughbred foals from vaccinated mares on a large stud farm. Equine Vet J 2004; 36(4):341-345.	Wrong study design
Kydd JH, Hannant D, Robinson RS, Bryant N, Osterrieder N. Vaccination of foals with a modified live, equid herpesvirus-1 gM deletion mutant (RacHΔgM) confers partial protection against infection. Vaccine. 2020;38(2):388-398.	Duplicate
Minke JM, Fischer L, Baudu P, Guigal PM, Sindle T, Mumford JA, Audonnet JC. Use of DNA and recombinant canarypox viral (ALVAC) vectors for equine herpes virus vaccination. Vet Immunol Immunopathol. 2006;111(1-2):47-57.	Duplicate
Moore BO, Koonse HJ. Inactivated equine herpesvirus I vaccine--Pneumabort-K. Proceedings of the Annual convention of the American Association of Equine Practitioners 1979;(pub. 1979):75-79.	Wrong comparator
Mumford EL, Traub-Dargatz JL, Carman J, Callan RJ, Collins JK, Goltz KL, Romm SR, Tarr SF, Salman MD. Occurrence of infectious upper respiratory tract disease and response to vaccination in horses on six sentinel premises in northern Colorado. Equine Vet J. 2003;35(1):72-7.	No challenge trial
Patel JR, Bateman H, Williams J, Didlick S. Derivation and characterisation of a live equid herpes virus-1 (EHV-1) vaccine to protect against abortion and respiratory disease due to EHV-1. Vet Microbiol. 2003;91(1):23-39.	Duplicate
Salco R, Bowers J, Hernandez V, Barnum S, Pusterla N. Prevention of respiratory infections with alpha- and gamma-herpesviruses in weanling foals by using a modified live intra-nasal equine influenza vaccine. Can Vet J. 2020;61(5):517-520.	No challenge trial

Soboll G, Breathnach CC, Kydd JH, Hussey SB, Mealey RM, Lunn DP. Vaccination of ponies with the IE gene of EHV-1 in a recombinant modified live vaccinia vector protects against clinical and virological disease. Vet Immunol Immunopathol. 2010;135(1-2):108-117. [electronic resource]	Duplicate
Soboll G, Hussey SB, Whalley JM, Allen GP, Koen MT, Santucci N, Fraser DG, Macklin MD, Swain WF, Lunn DP. Antibody and cellular immune responses following DNA vaccination and EHV-1 infection of ponies. Vet Immunol Immunopathol. 2006;111(1-2):81-95. [electronic resource]	Duplicate
Theunissen GT, van Essen GJ, van Maanen C, Schrijver RS. [Field trial with a subunit rhinopneumovaccine] Tijdschr Diergeneeskd 1995; 120(3):72-4.	No challenge trial
Traub-Dargatz JL, Pelzel-McCluskey AM, Creekmore LH, Geiser-Novotny S, Kasari TR, Wiedenheft AM, Bush EJ, Bjork KE. Case-control study of a multistate equine herpesvirus myeloencephalopathy outbreak. J Vet Intern Med. 2013;27(2):339-46. [electronic resource].	Duplicate
Waldman M. Effects of equine rhinopneumonitis vaccination on an epizootic of race track cough (tracheopharyngitis). Vet Med Small Anim Clin 1977; 72(4):594-6.	Wrong study design

Item 4: List of included studies

1. Bannai H, Nemoto M, Tsujimura K, Yamanaka T, Kokado H, Kondo T, Matsumura T. Comparison of protective efficacies between intranasal and intramuscular vaccination of horses with a modified live equine herpesvirus type-1 vaccine. Vet Microbiology. 2018; 222:18-24.
2. Bannai H, Tsujimura K, Nemoto M, Ohta M, Yamanaka T, Kokado H, Matsumura T. Epizootiological investigation of equine herpesvirus type 1 infection among Japanese racehorses before and after the replacement of an inactivated vaccine with a modified live vaccine. BMC Vet Res. 2019; 15(1):280.
3. Barrandeguy M, Lascombes F, Llorente J, Houssay H, Fernandez F. High case-rate equine herpesvirus-1 abortion outbreak in vaccinated polo mares in Argentina. Equine Vet Educ. 2002; 14(3):132-135.
4. Breathnach C, Yeargan M, Sheoran A, Allen G. The mucosal humoral immune response of the horse to infectice challenge and vaccination with equine herpesvirus-1 antigens. Equine Vet J. 2001; 33(7):651-657.
5. Burki F, Rossmanith W, Nowotny N, Pallan C, Mostl K, Lussy H. Viraemia and abortions are not prevented by two commercial equine herpesvirus-1 vaccines after experimental challenge of horses. Vet Q. 1990; 12(2):80-86.
6. Burrows R, Goodridge D, Denyer M. Trials of an inactivated equid herpesvirus 1 vaccine: challenge with a subtype 1 virus. Vet Rec 1984; 114(15):369-374.
7. Cornick J, Martens J, Martens R, Crandell R, McConnell S, Kit S. Safety and efficacy of a thymidine kinase negative equine herpesvirus-1 vaccine in young horses. Can J Vet Res. 1990; 54(2):260-266.
8. Dolby C, Hannant D, Mumford J. Response of ponies to adjuvanted EHV-1 whole virus vaccine and challenge with virus of the homologous strain. Br Vet J. 1995; 151(1):27-37.

9. Dutta SK, Shipley WD. Immunity and the level of neutralization antibodies in foals and mares vaccinated with a modified live-virus rhinopneumonitis vaccine. Am J Vet Res. 1975; 36(4 Pt.1):445-448.
10. Goehring L, Waner B, Bigbie R, Hussey S, Rao S, Morley P, Lunn D. Control of EHV-1 viremia and nasal shedding by commercial vaccines. Vaccine. 2010; 28(32):5203-5211.
11. Goodman L, Wagner B, Flaminio M, Sussman K, Metzger S, Holland R, Osterrieder N. Comparison of the efficacy of inactivated combination and modified-live virus vaccines against challenge infection with neuropathogenic equine herpesvirus type 1 (EHV-1). Vaccine. 2006; 24(17):3636-45.
12. Hannant D, Jesset D, O'Neill T, Dolby C, Cook R, Mumford J. Responses of ponies to equid herpesvirus-1 ISCOM vaccination and challenge with virus of the homologous strain. Res Vet Sci. 1993;54(3):299-305.
13. Heldens J, Hannant D, Cullinane A, Prendergast M, Mumford J, Nelly M, Kydd J, Weststrate M, Hoven R. Clinical and virological evaluation of the efficacy of an inactivated EHV1 and EHV4 whole virus vaccine (Duvaxyn EHV1,4) Vaccination/challenge experiments in foals and pregnant mares. Vaccine. 2001; 19(30):4307-4317.
14. Kydd J, Hannant D, Robinson R, Bryant N, Osterrieder N. Vaccination of foals with a modified live, equid herpesvirus-1 gM deletion mutant (RacHDeltagM) confers partial protection against infection. Vaccine. 2019; 38:388-398.
15. Kydd J, Wattrang E, Hannant D. Pre-infection frequencies of equine herpesvirus-1 specific, cytotoxic T lymphocytes correlate with protection against abortion following experimental infection of pregnant mares. Vet Immunol Immunopathol. 2003; 96(3-4):207-217.
16. Matsumura T, Ocallaghan D, Kondo T, Kamada M. Lack of virulence of the murine fibroblast adapted strain, Kentucky A (KyA), of equine herpesvirus type 1 (EHV-1) in young horses. Vet Microbiol. 1996; 48(34):353-365.

17. Minke J, Sindle T, Mumford J, Audonnet J, Fischer L, Baudu P, Guigal, P. Use of DNA and recombinant canarypox viral (ALVAC) vectors for equine herpes virus vaccination. Vet Immunol Immunopathol. 2006; 111(1-2):47-57.
18. Mitchell D, Girard A, Papp-Vid G. A challenge study on pregnant pony mares following vaccination with a modified live equine herpesvirus 1 (EHV-1) vaccine. J Equine Med Surg. 1978; 1(Suppl.): 69-73.
19. Mohd-Azmi M, Gibson J, Rixon F, McLauchlan J, Field H. Protection of specific-pathogen-free (spf) foals from severe equine herpesvirus type-1 (EHV-1) infection following immunization with non-infectious L-particles. J Microbiol. 2002; 40(3):183-192.
20. Mumford J, Bates J. Trials of an inactivated equid herpesvirus 1 vaccine: challenge with a subtype 2 virus. Vet Rec. 1984; 114(15):375-381.
21. Paillot R, Ellis SA, Daly JM, Audonnet JC, Minke JM, Davis-Poynter N, Hannant D, Kydd JH. Characterisation of CTL and IFN-gamma synthesis in ponies following vaccination with a NYVAC-based construct coding for EHV-1 immediate early gene, followed by challenge infection. Vaccine. 2006; 24(10):1490-1500.
22. Patel JR, Didlick S, Bateman H. Efficacy of a live equine herpesvirus-1 (EHV-1) strain C147 vaccine in foals with maternally-derived antibody: protection against EHV-1 infection. Equine Vet J 2004; 36(5):447-451.
23. Patel JR, Bateman H, Williams J, Didlick S. Derivation and characterisation of a live equid herpes virus-1 (EHV-1) vaccine to protect against abortion and respiratory disease due to EHV-1. Vet Microbiol. 2003;91(1):23-39.
24. Patel JR, Földi J, Bateman H, Williams J, Didlick S, Stark R. Equid herpesvirus (EHV-1) live vaccine strain C147: efficacy against respiratory diseases following EHV types 1 and 4 challenges. Vet Microbiol. 2003;92(1/2):1-17
25. Perkins G, Babasyan S, Stout AE, Freer H, Rollins A, Wimer CL, Wagner B. Intranasal IgG4/7 antibody responses protect horses against equid herpesvirus-1

(EHV-1) infection including nasal virus shedding and cell-associated viremia. Virology. 2019;531():219-232.

26. Purdy CW, Ford SJ, Porter RC. Equine rhinopneumonitis vaccine: immunogenicity and safety in adult horses, including pregnant mares. Am J Vet Res. 1978a; 39(3):377-83.
27. Purdy CW, Porter RC, Ford SJ. Equine rhinopneumonitis vaccine: immunogenicity and safety in foals. Am J Vet Res. 1978b;39(5):745-752.
28. Schnabel CL, Babasyan S, Rollins A, Freer H, Wimer CL, Perkins GA, Raza F, Osterrieder N, Wagner B. An equine herpesvirus type 1 (EHV-1) Ab4 open reading frame 2 deletion mutant provides immunity and protection from EHV-1 infection and disease. J Virol. 2019; 93(22):e01011-19.
29. Soboll G, Breathnach CC, Kydd JH, Hussey SB, Mealey RM, Lunn DP. Vaccination of ponies with the IE gene of EHV-1 in a recombinant modified live vaccinia vector protects against clinical and virological disease. Vet Immunol Immunopathol. 2010; 135(1-2):108-117.
30. Soboll G, Hussey SB, Whalley JM, Allen GP, Koen MT, Santucci N, Fraser DG, Macklin MD, Swain WF, Lunn DP. Antibody and cellular immune responses following DNA vaccination and EHV-1 infection of ponies. Vet Immunol Immunopathol. 2006; 111(1-2):81-95.
31. Thomson GR. Mumford JA, Smith IM. Experimental immunization against respiratory disease due to equid herpesvirus 1 infection (rhinopneumonitis) using formalin-inactivated virus with various adjuvants. Vet Microbiol 1979; 4(3):209-222.
32. Traub-Dargatz JL, Pelzel-McCluskey AM, Creekmore LH, Geiser-Novotny S, Kasari TR, Wiedenheft AM, Bush EJ, Bjork KE. Case-control study of a multistate equine herpesvirus myeloencephalopathy outbreak. J Vet Intern Med. 2013; 27(2):339-346.
33. Tsujimura K, Shiose T, Yamanaka T, Nemoto M, Kondo T, Matsumura T. Equine herpesvirus type 1 mutant defective in glycoprotein E gene as candidate vaccine strain. J Vet Med Sci. 2009; 71(11):1439-1448.

34. Van de Walle GR, May MA, Peters ST, Metzger SM, Rosas CT, Osterrieder N. A vectored equine herpesvirus type 1 (EHV-1) vaccine elicits protective immune responses against EHV-1 and H3N8 equine influenza virus. Vaccine. 2010; 28(4):1048-1055.

35. Wagner B, Perkins G, Babasyan S, Freer H, Keggan A, Goodman LB, Glaser A, Torsteinsdóttir S, Svansson V, Björnsdóttir S. Neonatal immunization with a single IL-4/antigen dose induces increased antibody responses after challenge infection with equine herpesvirus type 1 (EHV-1) at weanling age. Plos One 2017; 12(1):e0169072-e0169072.

Item 5: Risk of bias assessment of individual studies

Figure 1. Risk of bias (RoB) heatmaps of included studies. L: low risk of bias; U: unknown risk of bias; H: high risk of bias.

Domain	Bannai 2018	Bannai 2019	Barrandeguy 2002	Breathnach 2001	Burki 1990	Burrows 1984	Cornick 1990	Dolby 1995	Dutta 1975
Generation of allocation sequence	U	H	H	U	U	U	U	U	U
Groups similar at baseline	L	U	U	L	H	H	L	L	U
Allocation to groups was concealed	U	H	H	U	U	U	U	U	U
Animals randomly housed	U	U	U	U	U	U	L	U	U
Investigators blinded: intervention	U	H	U	U	U	U	U	U	U
Random selection of animals for outcome assessment	L	L	L	L	L	L	L	L	L
Blinding outcome assessor	U	U	U	U	U	U	U	U	U
Incomplete outcome data	L	U	L	L	L	L	L	L	L
Selective outcome reporting	L	L	L	L	L	L	L	L	L
Other RoB problems	L	U	H	L	H	U	U	U	U

Notes describing reasons for High RoB for individual studies. Bannai et al., (2019): Observational study in which the allocation was based on year at training center; year, and by availability of vaccine. Since the vaccines were given during different years the intervention were known to the investigators. Intermingling of mares among the four experimental groups occurred. Delays in vaccine administration occurred in one group. Burki et al., (1990): experimental study in which horses had different pre-study EHV-1 status, wide range (~ 20 yr) of ages were used, some demographic data was unreported. No virus challenges controls. Variable vaccination schedules were used. No statistical analysis was provided by the study authors. Burrows et al., 1994: experimental study in which In-foal mares had a large age range with pre-study EHV-1 exposure histories.

Domain	Goehring 2010	Goodman 2006	Hannant 1993	Heldens 2001	Kydd 2003	Kydd 2019	Matsumura 1996	Minke 2006	Mitchell 1978
Generation of allocation sequence	L	L	L	U	U	H	U	L	U
Groups similar at baseline	L	L	U	L	H	L	U	U	H
Allocation to groups was concealed	L	L	U	U	U	U	U	U	U
Animals randomly housed	L	U	U	H	U	U	U	U	U
Investigators blinded: intervention	L	L	U	U	U	U	U	U	U
Random selection of animals for outcome assessment	L	L	L	L	L	L	L	L	L
Blinding outcome assessor	L	L	U	U	U	L	U	U	U
Incomplete outcome data	L	L	L	L	L	L	L	L	U
Selective outcome reporting	L	L	L	L	L	L	L	L	U
Other RoB problems	U	L	L	L	U	L	U	U	U

Notes describing reasons for High RoB for individual studies. Heldens et al., (2001): experimental study in which controls were housed separately from vaccinated foals. Kydd et al., (2003): experimental study in which pregnant mares had a wide range of ages (> 10 yr range). All the mares in each group were infected together, on three separate occasions, with a virulent strain of EHV-1 when they were between 32 and 42 weeks of gestation. While the number of previous experimental infections experienced by all the mares was known, their field infection history and latency status was not. Kydd et al., (2019): experimental study in which allocation was made by a nonrandom method (birth date of the foals). Mitchell et al., (1978): experimental study in which SN titers prior to challenge were dissimilar and ages of ponies was undefined.

Domain	Mohd-Azmi 2002	Mumford 1984	Paillot 2006	Patel 2003a	Patel 2003b	Patel 2004	Perkins 2019	Purdy 1978b	Purdy 1978a
Generation of allocation sequence	U	U	U	U	U	H	L	U	U
Groups similar at baseline	L	U	L	U	L	L	L	H	H
Allocation to groups was concealed	U	U	U	U	U	U	U	U	U
Animals randomly housed	U	U	U	U	U	U	U	U	U
Investigators blinded: intervention	U	U	U	U	U	U	U	U	U
Random selection of animals for outcome assessment	L	L	L	L	L	L	L	U	U
Blinding outcome assessor	U	U	U	U	U	U	L	U	U
Incomplete outcome data	L	L	L	U	L	U	L	U	L
Selective outcome reporting	L	L	L	L	L	L	L	L	L
Other RoB problems	U	U	L	U	U	U	L	H	H

Notes describing reasons for High RoB for individual studies. Patel et al., (2004): experimental study in which allocation to groups used a nonrandom approach based on age and VN titers. Purdy et al., (1978a; b): experimental studies in which pre-study vaccination status, ages, breeds, and exposure status were not described. Statistical methods were not provided.

Domain	Schnabel 2019	Soboll 2006	Soboll 2010	Thomson 1979	Traub-Dargatz 2013	Tsujimura 2009	Van de Walle 2010	Wagner 2017
Generation of allocation sequence	L	U	H	U	L	U	U	L
Groups similar at baseline	L	L	U	U	U	L	H	L
Allocation to groups was concealed	U	U	U	U	U	U	U	U
Animals randomly housed	L	U	U	U	U	U	U	U
Investigators blinded: intervention	U	U	U	U	U	U	U	U
Random selection of animals for outcome assessment	L	L	L	L	U	L	L	L
Blinding outcome assessor	L	U	U	U	U	U	L	L
Incomplete outcome data	L	L	L	L	L	L	L	L
Selective outcome reporting	L	L	L	L	L	L	L	L
Other RoB problems	L	L	L	L	H	L	U	U

Notes describing reasons for High RoB for individual studies. Soboll et al., (2010): experimental study in which allocation to groups used a nonrandom approach based on their MCH-1 haplotype. Traub-Dargatzet al., (2013): observational study in which inherent non-response/self-selection bias with this type of study. Possible researcher selection bias. Van de Walle et al., (2010)): experimental study in which a wide range of ages were used (2 to 18 yrs).

Supplemental Table 1. Key characteristics of experimental studies.

Study	Vaccine	Type	Route	Subjects	Controls	Dose	Vaccine doses	Vaccine Interval/ timing	Study design	Comparator	Challenge Strain	Challenge Exposure	Challenge Route	Challenge post last vaccination	Comments	Funding
Bannai et al., 2018	Equine Rhinopneumonitis Vaccine	Commercial MLV	IM	5	5	NR	2	q 4 wk	Non-randomized, controlled study	Vaccine vs. unvaccinated	10-I224	4 × 10^6 PFU	IN	4 wk		Japan Racing Association
			IN	5												
Breathnach et al., 2001	Army 183	Experimental MLV	IN	4	2	7.05 x 10^7 PFU	2	q 3 wk	Partially randomized, controlled study	Vaccine vs. unvaccinated	Army 183	7.05 x 10^7 PFU	IN	10 wk		University of Kentucky Equine Research Foundation and the Geoffrey C. Hughes Foundation
	Rhinomune	Commercial MLV		4										3 wk		
	Pneumabort-K	Commercial Killed	IM	4		Mfr										
	Rhinomune/ Pneumabort-K	Mixed Commercial	IN/IM	4		7.05 x 10^7 PFU/Mfr										
Bürki et al., 1990	Prevacinol	Commercial MLV	IM	4	0	Mfr	3-6	Variable @ 3-4 and 7-9 MOG	Non-randomized, noncontrolled study	Vaccine vs. unvaccinated	Piber 178/83	$10^{7.0}$ $TCID_{50}$	IN	3-15 wk	PM	NR
	Pneumabort-K	Commercial Killed	IM	6			4-7	Variable @ 5, 7 and 9 MOG								
	Prevacinol	Commercial MLV	IM	5	2		2-5	Variable						3-16 wk	Adult and 1 yo	
	Pneumabort-K	Commercial Killed	IM	3			3-4							3 wk		
Burrows et al., 1984	Pneumabort-K	Commercial Killed	NR	6	4	NR	2	q 5 mo	Non-randomized, controlled study	Vaccine vs. unvaccinated	3551/80	Nebulized 60 X $10^{5.5}$ PFU	INH (20 min)	1 mo	1 yo	Horserace Betting Levy Board
				12	7		1							2 mo	2 yo	
				17	9		3	@ 5, 7, and 9 MOG						1 mo	PM	
Cornick et al., 1990	TK-EHV-1 (H6b mutant)	Deletion mutant	IM	6	4	7 X 10^8 PFU/ml	1		Non-randomized, placebo controlled study	Vaccine vs. placebo	Army 183	3 X 10^6 $TCID_{50}$	IN	45 d		USDA and Kleberg Foundation
			IV	2												
Dolby et al., 1995	EHV-1, strain V592	Inactivated experimental	IM	5	3	25 µg viral protein	2	q 6 wk	Non-randomized, controlled study	Vaccine vs. unvaccinated	V592	$10^{6.9}$ $TCID_{50}$	IN	6 wk	Freund's complete adjuvant (dose 1), Freund's Incomplete adjuvant (dose 2)	Horserace Betting Levy Board, Thoroughbred Breeders' Association and Pitman Moore Europe
Goehring et al., 2010	Rhinomune	Commercial MLV	IM	8	8	Mfr	3	day 0, 27, and 97	Randomized, blinded, controlled study	Vaccine vs. unvaccinated	Findlay OH03	5 × 10^7 PFU	IN	24 d		Fort Dodge Animal Health
	Pneumabort-K	Commercial Killed		8												

Goodman et al., 2006	Flu- vac Innovator 6	Commercial Killed	IM	5	5	Mfr	2	q 30 d	Randomized, blinded, placebo controlled	Vaccine vs. placebo	Findlay OH03	5×10^6 PFU	INH (nebulized 12 min) and IN	29 d		Harry M. Zweig Memorial Fund for Equine Research and Pfizer Animal Health
	Rhinomune	Commercial MLV		5												
Hannant et al., 1993	immune stimulating complex vaccine	Inactivated experimental	IM	9	6	~ 25 µg viral protein	3	@ 0, 5, 19 wk	Randomized, controlled study	Vaccine vs. unvaccinated	V592	$10^{6.6}$ $TCID_{50}$	INH (nebulized 12 min)	3 wk		Horserace Betting Levy Board, Thoroughbred Breeders' Association, Pitman Moore Europe, and Iscotec
Heldens et al., 2001	Duvaxyn EHV1,4	Commercial Killed	IM	10	5	Mfr	2	q 4 wk	Randomized, controlled study	Vaccine vs. unvaccinated	121412	$10^{5.0}$ $TCID_{50}$	IN	2 wk	Foals	NR
			IM	5	4		3	@ 5, 7, and 9 MOG	Non-randomized, controlled study		AB4	$10^{6.0}$ $TCID_{50}$	IN	4 wk	PM	
Kydd et al., 2003	Duvaxyn EHV-1/4	Commercial Killed	IM	5	9	NR	3	@ 5, 7, and 9 MOG	Randomized, controlled study	Vaccine vs. unvaccinated	AB4/8	10^5 $TCID_{50}$	IN	4 wk	PM	Horserace Betting Levy Board, Equine Virology Research Foundation, Fort Dodge Animal Health and the Animal Health Trust
Kydd et al., 2020	EHV-1 strain RacHΔgM	Deletion mutant	IN and IM	6	6	NR	2	q 6 wk	Non-randomized, blinded, controlled study	Vaccine vs. unvaccinated	Ab4/8	2×10^6 $TCID_{50}$	IN	4 wk		Animal Health Trust, and German Research Foundation
Matsumura et al., 1996	EHV1 strain KyA	Experimental MLV	IN	4	2	10^7 $TCID_{50}$	1		Non-randomized, controlled study	Vaccine vs. unvaccinated	89c25	10^6 $TCID_{50}$	IN	4 wk		NIH, USDA
Minke et al., 2006	ALVAC-EHV	Recombinant	IM	5	5	10^8 $TCID_{50}$	2	q 5 wk	Randomized, placebo controlled	Vaccine vs. unvaccinated given adjuvant	Ab4	10^5 $TCID_{50}$	IN	3 wk		Animal Health Trust and Merial R&D
	ALVAC-EHV with Carbopol			5												
	ALVAC-EIV with Carbopol			5												
	gB/gC/gD plasmids	DNA	IM	5	5	500 µg	2	q 5 wk	Randomized, placebo controlled	Vaccine vs. unvaccinated				2 wk		
	gB/gC/gD plasmids and $Al(PO)_4$			5												
	Plasmids gB, gC, and gD and Carbopol			5												
	gB/gC/gD plasmids and DMRIE-DOPE			5												

Mitchell et al., 1978	Rhinomune	Commercial MLV	IM	4	2	Mfr	2	q 4 wk	Non-randomized, controlled study	Vaccine vs. unvaccinated	EHV1 strain NR (isolated from aborted fetus)	5 X $10^{5.3}$ $TCID_{50}$	50% IV, 50% IM	14 d		NR
Mohd-Azmi et al., 2002	EHV-1 strain AB4p L particles	Inactivated experimental	IN	1	2	10^9 L-particles	2	q 4 wk	Non-randomized, controlled study	Vaccine vs. unvaccinated	Ab4	10^7 PFU	IN	4 wk		NR
	EHV-1 strain AB4p L particles			1												
	EHV-1 strain AB4p UV inactivated			1		10^6 PFU										
Mumford and Bates, 1984	Pneumabort-K	Commercial Killed	IM	15	13	Mfr	2	q 4 wk	Non-randomized, controlled study	Vaccine vs. unvaccinated	R500	10^7 $TCID_{50}$	IN	12 wk		Horserace Betting Levy Board
				15			3	@ 0, 4, 10 wk								
Paillot et al., 2006	vP1014 and carbomer-PD adjuvant	Recombinant	IM	1	1	10^7 pfu	1		Non-randomized, placebo controlled study	Vaccine vs. placebo	Ab4/8	2 X 10^5 $TCID_{50}$	IN	12 wk		Horserace Betting Levy Board
				1			3	q 3 wk			Ab4/8	2 X 10^5 $TCID_{50}$	IN	3 wk		
				1			4	q 3 wk			Ab4/8	2 X 10^5 $TCID_{50}$	IN	3 wk		
Patel et al., 2003a	EHV-1 strain M8, C147	Experimental MLV	IN	5	6	5.2 $TCID_{50}$	1		Nonrandomized, controlled study	Vaccine vs. unvaccinated	AB4	$10^{5.7}$ $TCID_{50}$	IN	4 mo	PM	NR
				6										5-6 mo		
Patel et al., 2003b	EHV-1, Strain C147	Experimental MLV	IN	8	8	5.2 $TCID_{50}$	1		Non-randomized, controlled study	Vaccine vs. unvaccinated	AB4	10^6 $TCID_{50}$	IN	6 wk		NR
Patel et al., 2004	EHV-1 Strain C147	Experimental MLV	IN	8	6	5.0 $TCID_{50}$	1		Non-randomized, controlled study	Vaccine vs. unvaccinated	AB4	10^6 $TCID_{50}$	IN	8 wk		NR
Perkins et al., 2019	EHV-1 Strain Ab4	Experimental MLV	IN	5	5	1 × 10^7 PFU	1		Randomized, blinded, controlled study	Vaccine vs. unvaccinated	Ab4	10^7 PFU	INH (mucosal atomizer)	6 mo		Harry M. Zweig Memorial Fund for Equine Research and USDA
	EHV-1 Strain Ab4ΔORF1/71	MLV	IN	5												
Purdy et al., 1978a	Rhinoquin	Commercial MLV	IM	10	8 (PM = 3)	$10^{6.5}$ $TCID_{50}$	1		Non-randomized, controlled study	Vaccine vs. unvaccinated	NR from aborted fetus	$10^{4.2}$ $TCID_{50}$	IN	40 d	PM = 8	NR
				10			2	21 d						50 d	PM = 9	
				5	2 (PM = 0)		2	21 d						1 y	PM = 4	
				9	2		1								Adults	
Purdy et al., 1978b	Rhinoquin	Commercial MLV	IM	5	3	$10^{6.5}$ $TCID_{50}$	1		Non-randomized, controlled study	Vaccine vs. unvaccinated	NR from aborted fetus	$10^{4.2}$ $TCID_{50}$	IN	77 – 127 d	Vaccinated at 14 d of age. Experiment #3 (Rx	NR

															= G; PBO = I)	
				7										108-172 d	Vaccinated at 1 d of age. Experiment #4 (Rx = J)	
				10	5									84-144 d	Vaccinated at 7-12 d of age. Experiment #4 (Rx = K)	
				14										91-128	Vaccinated at 100 d of age. Experiment #4 (Rx = L)	
				12	4									1 yr	Vaccinated at 1-12 d of age. Experiment #5 (Rx = N)	
				9	4									1 yr	Vaccinated at 7-42 d of age. Experiment #5 (Rx = O)	
				7	4									1 yr	Vaccinated at 122 d of age. Experiment #5 (Rx = S)	
Schnabel et al., 2019	EHV1 strain Ab4/8	Experimental MLV	IN	8	8	1 x 10^7 PFU	1		Randomized, blinded, controlled study	Vaccine vs. unvaccinated	AB4/8	10^7 PFU	IN	9 mo		USDA and Harry M. Zweig Memorial Fund for Equine Research
	EHV1 strain Ab4ΔORF2	Deletion mutant	IN	8												
Soboll et al., 2006	gB/gC/gD plasmids	DNA	ID	5	5	25 µg	4	@ 0, 41, 86, and 126 d.	Non-randomized, controlled study	Vaccine vs. unvaccinated	Army 183	2 X 10^7 PFU	IN	4 wk	Vaccinated at 32 skin and 32 mucosal sites	USDA and Grayson-Jockey Club Research Foundation
	IE/UL5 plasmids		ID	5												
Soboll et al., 2010	rMVA-IE	Recombinant	Dose #1 and 2: ID (50%), IM (50%) Dose #3: IV	10	5	Doses # 1 and 2: 6 x 10^8 PFU Dose 3: 6 x10^8 PFU	3	@ 0, 33, and 124 d	Non-randomized, controlled study	Vaccine vs. unvaccinated	Army 183	5 x 10^7 PFU	IN	8 wk	ELA-A3/B2 genotype (A3/B2)	USDA and Grayson-Jockey Club Research Foundation
				5											ELA-A3 genotype/ no B2 allele (A3-non-B2)	
				6											Haplotypes other than A3 (non- A3)	
	Rac-H and alhydrogel	Inactivated experimental	IM	4	8		2	q 6 wk	Non-randomized,	Vaccine vs. placebo	RAC-H	$10^{9.4}$ TCD_{50}	IN	6 wk	Foals	

Thomson et al., 1979	Rac-H and adjuvant 65			4		250 µg viral protein			placebo controlled study							Horserace Betting Levy Board
	Rac-H and levamisole			4												
	Rac-H and alhydrogel			4	9	900 µg viral protein									Yearlings	
	Rac-H and adjuvant 65			4												
	Rac-H and Freuds complete adjuvant			3												
	Rac-H and *C. parvum*			4												
Tsujimura et al., 2009	ΔgE EHV-1 strain	Deletion mutant	IM	3	3	Dose 1: 10^5 PFU<br>Dose 2: 10^6 PFU	2	q 3 wk	No-nrandomized, placebo controlled study	Vaccine vs. placebo	89C25p	10^6 PFU	IN	4 wk	39-80 d old foals	NR
				3											28-92 d old foals	
Van de Walle et al., 2009	rNY03ΔIR6/1gp2S	Deletion mutant	IM and SC (50:50)	8	4	5 × 10^6 PFU	2	q 25d	Nonrandomized,-placebo controlled study	Vaccine vs. placebo	Findlay OH03	2 x 10^7 PFU	IN	36d		Harry M. Zweig Memorial Fund for Equine Research and Pfizer, Inc
Wagner et al., 2017	Streptavidin-conjugated gC/IL-4 fusion protein (Sav-gC/IL-4)	Inactivated experimental	IM	5	5	1 mg IgE-bio and 500 µg viral protein	1	@ 2 days old	Non-randomized, controlled study	Vaccine vs. unvaccinated	NY03	10^7 PFU	IN	7 mo	IgE-bio given at birth	Harry M. Zweig Memorial Fund for Equine Research
				5		500 µg viral protein										

Abbreviations: ALVAC-EHV: recombinant canarypox virus (vCP132) expressing the gB, gC and gD glycoproteins of the Kentucky strain of EHV- 1; ALVAC-EIV: recombinant canarypox virus (vCP1502) expressing the haemagglutinin of influenza A/eq/Prague/56 (H7N7); DMRIE/DOPE: [N-(1-(2,3-di- myristyloxypropyl)-N,N-dimethyl-(2-hydroxy-ethyl) ammoniumbromide/dioleoyl) phosphatidylethanola- mine; ID: intradermal; Ig: immunoglobulin; IE/UL5: immediate early (IE) and early proteins (UL5) of EHV-1; IM: intramuscular; IN: intranasal; INH: inhalation; mfr: per manufacturer recommendations; MLV: modified-live virus; mo: month; MOG: months of gestation; NR: not reported; PBMC: peripheral blood mononuclear cells; PFU: plaque-forming units; PM: pregnant mare; q: every; rMVA-IE: recombinant modified vaccinia Ankara vector expressing the IE gene; TK: thymidine kinase; vP1014: vaccinia-based construct (NYVAC) that codes for an immediate early gene (gene 64) of EHV-1; wk: week

Supplemental Table 2. Selected demographic characteristics of study populations.

Study	Breed	Sex	Age	Pre-study EHV-1 status
Bannai et al., 2018	Thoroughbred	F	16 to 21 mo	Pre-existing antibodies suggesting natural exposure as determined by glycoprotein E1-ELISA
Bannai et al., 2019	NR	NR	3 to ≥ 8 y	Negative ELISA for EHV-1 or known positive vaccination status
Barrandeguy et al., 2002	NR	F	NR	Not reported for most horses). Known previous vaccination for remaining horses.
Breathnach et al., 2001	Mixed breed horse and pony	NR	5 to 7 mo	Residual maternal EHV-1 specific serum antibodies
Burki et al., 1990	Halfinger<br>Thoroughbred<br>Shetland Pony	F or NR	Vaccinates: 1 to 19 y<br>Controls: 2 to 4 y	Negative serological titers in sentinel horses during year before experiment. Haflinger and Thoroughbreds had a silent EHV-1 infection early in the study.
Burrows et al., 1984	Welsh Mountain ponies	F or NR	1 to ≥ 6 y or NR	Negative serum antibodies; known exposure to EHV-1; or NR
Cornick et al., 1990	NR	NR	5 to 7 mo	Negative neutralizing antibody titer
Dolby et al., 1995	Welsh-cross ponies	NR	1 y	Serum antibody consistent with previous natural EHV-1 or EHV-4 infections
Dutta, 1975	NR	F or NR	1-4 mo or NR	SN < 4 – 56 or NR
Goehring et al., 2010	Mixed-breed pony	F or NR	11 to 13 mo	Negative SN titers- Controls: 1.2-1.8; Vaccinates: 1.2-1.8
Goodman et al., 2006	Mixed-breed	F	3 to 10 y	Negative EHV-1 neutralization titers (SN < 1:24)
Hannant et al., 1993	Welsh Mountain Ponies	NR	1 y	No serological evidence of exposure to EHV-1 or EHV-4
Heldens et al., 2001	Irish breeds<br>Welsh Mountain Ponies	F or NR	5 mo to 3 y	Negative VN or CF antibodies for EHV-1 and EHV-4 or positive CF and VN antibodies to EHV-1 or EHV-4
Kydd et al., 2003	Pony	F	3 to 20 y	Controls: pre-infection CF titer < 1.5.<br>Vaccinates: pre-infection CF titer < 3
Kydd et al., 2020	Welsh Mountain Ponies	M and F	4 to 10 mo	Negative for CF antibodies for EHV-1. Declining colostrum-derived EHV-1 serum VN antibodies.
Matsumura et al., 1996	Half-bred and Thoroughbred	NR	< 1.5 mo to 11 mo	Negative for neutralizing antibodies (EHV-1)
Minke et al., 2006	Welsh Mountain Ponies	M and F	1-2 y	Negative CF antibody, EHV-1 and EHV-4. Positive detection of VN antibody, EHV-1 or EHV-4
Mitchell et al., 1978	Ponies	F	NR	NR

Mohd-Azmi et al., 2002	Welsh pony	NR	< 1 y	EHV-1 naïve herd (SPF)
Mumford, 1984	Welsh Mountain Ponies	NR	8 to 12 mo	NR
Paillot et al., 2006	Welsh mountain ponies	NR	1 to 2 y	Field infection with EHV-1 or EHV-4 prior to vaccination; detected by seroconversion. CTL < 13% immediately prior to vaccination.
Patel et al., 2003	Welsh mountain ponies	PF	NR	No or low virus neutralizing antibody to EHV-1 or EHV-4.
Patel et al., 2003	Hungarian half-breed	M and F	15 to24 mo	NR
Patel et al., 2004	Ponies	NR	44 to 119 d	Born to vaccinated mares. Moderately to highly positive for SN antibody to EHV-1 or EHV-4.
Perkins et al., 2019	Icelandic	NR	2.5 y	EHV-1 naïve herd
Purdy et al., 1978a	NR	M and F	NR	NR
Purdy et al., 1978b	NR	NR	13 to 46 d	NR
Schnabel et al., 2019	Icelandic	M and F	3 to 5 y	No infection or prior Ab4ΔORF2 or Ab4 infection
Soboll et al., 2006	Ponies	NR	1 y	NR
Soboll et al., 2010	Ponies	NR	2 to 7 y	Previously exposed > 12 mo ago
Thomson et al., 1979	Welsh mountain ponies	NR	4 mo to 2 y	Some animals had serological evidence of prior infection
Traub-Dargatz et al., 2013	NR	M and F	<5 to ≥ 10 y	Previous vaccination before exposure
Tsujimura et al., 2009	Thoroughbred	NR	25 to 92 d	NR
Van de Walle et al., 2010	NR	NR	2 to 18 y	SN titer < 1:24
Wagner et al., 2017	Icelandic	M and F	1 to 2 d	EHV-1 naïve herd

Abbreviations: CF: complement fixing; CTL: cytotoxic T lymphocyte; ELISA: enzyme-linked immunosorbent assay; F: female; GM: geometric mean; M: male; mo: month; NR: not reported; PF: pregnant female; PCR: polymerase chain reaction; SN: serum neutralization; SPF: specific pathogen free; y: year; VN: virus neutralizing

Supplemental Table 3. Study results. Unless otherwise noted data presented is mean ± SD. Shaded cells denote data extracted from observational studies.

Study	Vaccines	Comparators	Results following challenge	Comments
Bannai et al., 2018	Equine Rhinopneumonitis Vaccine	IM Vaccine vs. unvaccinated	Incidence of pyrexia @ 1-4 dpi: NSD (Vx = 1/5; CON = 3/5). Incidence of pyrexia @ 5-10 dpi: NSD (Vx = 2/5; CON = 3/5). Mean RT: ↓ @ 1 and 8 dpi (RX ~ 38 °C; CON ~ 38.5°C). Duration of pyrexia: NSD. Mean nasal discharge score: NSD (Vx = 2.8; CON = 5.0). Mean lymph node score: ↓ (Vx = 1; CON = 5.0; p < 0.05). Incidence of nasal shedding: NSD (Vx = 3/5; CON = 5/5). Maximum viral shedding (mean): NSD (Vx = 2.4, CON = 5.0 log10 PFU/ml). SN titer: ↑ @ 14, 21, and 35 dpi. Incidence of viremia: NSD (Vx = 3/5; CON = 5/5). Duration of viremia: NSD (Rx= 3.6 ± 3.4 d; CON = 3.0 ± 1.0 d)	Experimental challenge. Small Rx and CON group sizes (n = 5/group). No adverse clinical reactions. Scored nasal discharge and lymphadenopathy and cumulative scores calculated. Pyrexia = RT ≥ 38.5 °C. p < 0.05. Post hoc analysis of some incidence data performed (p < 0.05). Viremia and nasal shedding evaluated using plaque assay (RK13 cells).
		IN Vaccine vs. unvaccinated	Incidence of pyrexia @ 1-4 dpi: ↓ (Vx = 0/5; CON = 3/5). Incidence of pyrexia @ 5-10 dpi: NSD (Vx = 3/5; CON = 3/5). Mean RT: ↓ @ 1 and 2 dpi. Duration of pyrexia: NSD. Mean nasal discharge score: NSD (Vx = 2.4; CON = 5.0). Mean lymph node score: ↓ (Vx = 1; CON = 5.0; p < 0.05). Incidence of nasal shedding: NSD (Vx = 5/5; CON = 5/5). Maximum viral shedding (mean): NSD (Vx = 3.7, CON = 5.0 log10 PFU/ml). SN titer: ↑ @ 14, 21, and 35 dpi. Incidence of viremia: NSD (Vx = 5/5; CON = 5/5). Duration of viremia: NSD (Vx = 2.3 ± 1.0 d; CON = 3.0 ± 1.0 d)	
Bannai et al., 2019	Equine Rhinopneumonitis Vaccine	None	Serum VN titer: GM approximate 2-fold ↑ after 1st dose. Response rate = 42%. EHV1 infection rate = 52-66%. Mean number of pyretic horses = 126 ± 10;	Data for 3-year-old horses only. Natural infection. Experiment performed sequentially over several different years. Approximately 1000 horses were exchanged between the training centers and the farms every month. Pyrexia = RT ≥ 38.5 °C. p < 0.05.
	Equi N Tect ERP	None	Serum VN titer: GM approximate 5-fold ↑ after 1st dose. Response rate: 76-90%. EHV1 infection rate = 12-24%. Mean number of pyretic horses = 105 ± 5.	
		Equi N Tect ERP vs. Equine Rhinopneumonitis Vaccine	Serum VN titer (GM) and response rate: MLV > inactivated. EHV1 infection rate and mean number of pyretic horses: Inactivated > MLV.	
Barrandeguy et al., 2002	Vaccine 1	None	Abortion rate = 17/69 (25%). No signs of respiratory or neurologic disease. Number of foal deaths = 3.	Natural infection. Vaccine sources were NR. Intermixing of different Rx groups. Serum VN Ab titers ranged from 1:32 to 1:512. Pyrexia = RT ≥ 38.8 °C. Statistical comparisons were not performed.
	Vaccine 2 (Groups B and D)	None	Abortion rate = 5/24 (20%) to 12/21 (57%) in Groups B and D respectively. No signs of respiratory or neurologic disease. No foal deaths.	
	Vaccine 3 (Group C)	None	Abortion rate = 16/59 (27%). No signs of respiratory or neurologic disease. Number of foal deaths = 2.	
Breathnach et al., 2001	Army 183	Vaccine vs. unvaccinated	Mean days with pyrexia: ↓ (Vx = 0.2 d, CON = 14 d). Mean days with nasal discharge ≥ grade 1 ↓ (Vx = 0 d, CON = 13.5 d). Incidence of viremia: NSD (Rx= 0/4; CON = 2/2). Mean days shedding virus: ↓ (Vx = 0.7 d, CON = 9 d); days with viremia: ↓ (Vx = 0; CON = 7.5).	Experimental challenge. Small Rx group sizes (n = 4 per group); n = 2 (CON). Ab levels reported as μg Ab/mg IgA (mean ± SE). Pyrexia = RT ≥ 38.8°C. Antipyretics were administered to horses with a RT ≥ 39.4°C. p < 0.05. Post hoc analysis of incidence data performed (p < 0.05). Viremia and nasal shedding evaluated using plaque assay (KyED cells) and PCR.
	Rhinomune		Mean days with pyrexia: NSD (Vx = 4.3 d, CON = 14 d). Mean days with nasal discharge ≥ grade 1 ↓ (Vx = 5.5 d, CON = 13.5) d. Incidence of viremia: NSD (Rx= 0/4; CON = 2/2). Mean days shedding virus: ↓ (Vx = 3 d, CON = 9 d); days with viremia: ↓ (Vx = 0 d; CON = 7.5 d). Serum titer: NSD. Peak total Ab = 2.1 ± 0.5 μg/mg IgA @ 2 wk pi. Peak (@ 2 wk pi) IgA = 2.5 ± 1.0; IgGa = 0.6 ± 0.1; IgGb = 0.04 ± 0.1; IgGt = nd.	
	Pneumabort K		Mean days with pyrexia: NSD (Vx = 5.5 d, CON = 14 d), days with nasal discharge ≥ grade 1: NSD (Vx = 8 d, CON = 13.5 d). Incidence of viremia: NSD (Rx= 0/4; CON = 2/2). Mean days with viremia: ↓ (Vx = 0 d; CON = 7.5 d). Mean days shedding virus: NSD (Vx = 4.3 d, CON = 9 d). Serum titer: ↑. Peak total Ab= 13.4 ± 5.6 μg/mg IgA @ 1 wk pi. Peak mean IgA = 9.9 ± 3.8 @ 3 wk pi; IgGa = 3.6 ± 1.5 @ 1 wk pi; IgGb = 3.0 ± 1.7 @ 3 wk pi; IgGt = 1.6 ± 0.8 @ 3 wk pi.	

	Rhinomune and Pneumabort K		Mean days with pyrexia: NSD (Vx = 6.7 d, CON = 14 d). Mean days with nasal discharge of ≥ grade 1: NSD (Vx = 11.7 d, CON = 13.5 d). Incidence of viremia: NSD (Rx= 0/4; CON = 2/2). Mean days with viremia: ↓ (Vx = 0 d; CON = 7.5 d). Mean days shedding virus: NSD (Vx = 3.3 d, CON = 9 d). Serum titer: NSD. Peak total Ab = 8.9 ± 2.4 μg/mg IgA @ 2 wk pi. Peak IgA= 4.4 ± 1.0 @ 3 wk pi; IgGa = 3.9 ± 1.5 @ 1 wk pi; IgGb = 0.4 ± 0.2 @ 3 wk pi; IgGt = 0.3 ± 0.2 @ 3 wk pi.	
Bürki et al., 1990	Prevacinol	None	Incidence of abortion (2/4), fever (PM = 3/4; Others = 5/5); nasal and ocular discharge (PM 4/4; Others = 4/5); spontaneous cough (PM = 2/4; Others = 1/5), nasal shedding @ 2 dpi (PM = 4/4; Others = 3/5), and viremia on ≥ 1 dpi (PM = 3/4; Others = 5/5). Pre-challenge SN titer: GM = 17 (range 4-45).	Experimental challenge. Small Rx group sizes (n = 4 per group) with variable vaccination schedules. Two sentinels. Some Rx horses were EHV-1 exposed during the trial. Pyrexia = RT ≥ 36.6°C. Statistical comparisons were not performed. Methods to evaluate viremia and nasal shedding were not described.
	Pneumabort-K		Incidence of abortion (3/6), fever (PM = 4/6; Others = 1/3); nasal and ocular discharge (PF 6/6; Others = 3/3); spontaneous cough (PM = 0/6; Others = 0/3), nasal shedding @ 2 dpi (PM = 4/6; Others = 3/3), and viremia on ≥ 1 dpi (PM = 6/6; Others = 2/3). Pre-challenge SN titer: GM = 47 (range 32-91).	
Burrows et al., 1984	Pneumabort-K	1yo: Age matched Vaccine vs. unvaccinated	Incidence of viremia and fever: NSD (Vx = 6/6, CON = 4/4). Incidence of lymphadenopathy: NSD (Vx = 0/6, CON = 0/4; p = 0.09). Clinical score (fever and other signs): NSD (Vx = 14.8; CON = 22.3). Nasal shedding (GM) = 2.90 (CON = 5.21). Viremia (GM) = 1.35 (CON = 1.30). ≥ 4 ↑ SN titer (CF) after 1st dose = 8/12; after 2nd dose = 10/12.	Experimental challenge with nebulized virus – insufficient details provided to reconstruct exposure dose. Yearlings receiving 3 doses of vaccine were not challenged and not included. Two PF and 2 CON not in foal. Clinical score considered RT (pyrexia = RT > 38.5°C), duration of nasal discharge, conjunctivitis, lymphadenopathy, and cough. Statistical analyses not provided. Post hoc statistical analysis performed on select data. No adverse effects reported. Viremia and nasal shedding evaluated using plaque assay (RK13 cells).
		2 yo: Age matched Vaccine vs. unvaccinated	Clinical score (fever and other signs): NSD (Vx = 11.2 and 16.2; CON = 21.3). Incidence of viremia and fever: NSD (Vx = 12/12, CON = 7/7). Incidence of lymphadenopathy: NSD (Vx = 2/12, CON = 4/7; p = 0.09). Nasal shedding (GM) = 3.48 and 3.77 (CON = 4.85). Viremia (GM) = 1.01 and 1.24 (CON = 1.41). ≥ 4 ↑ SN titer (CF) after 1st dose = 6/12; after 2nd dose = 12/12.	
		PF: Age matched P Vaccine vs. unvaccinated	Incidence of fever: NSD (Vx = 14/17; CON = 8/9). Clinical score: NSD (Rx$_{2doses}$ = 1.1; Rx$_{3\ doses}$ = 4.2; CON = 5.4). Incidence of fetal infection: NSD (Rx$_{2doses}$ = 3/8; Rx$_{3\ doses}$ = 4/8; CON = 3/8). Incidence of abortion: NSD (Rx$_{2doses}$ = 3/7; Rx$_{3\ doses}$ = 2/8; CON = 2/7). Incidence of infected foals: NSD (Rx$_{2doses}$ = 0/7; Rx$_{3\ doses}$ = 1/8; CON = 0/7). Incidence of viremia: NSD (Vx = 15/17, CON = 5/7). ≥ 4 ↑ SN titer (CF) after 1st dose: PM = 15/17; after 2nd dose: PM = 14/17. VN titer (GM) Rx$_{2doses}$ = 1.45; Rx (3 doses) = 1.34; CON = < 0.8. Nasal titer (GM): Rx$_{2doses}$ = 2.86; Rx$_{3\ doses}$ = 2.95; CON = 3.12; NSD. PBMC titer (GM): Rx$_{2doses}$ = 1.30; Rx$_{3\ doses}$ = 1.56; CON = 1.16.	
Cornick et al., 1990	TK-EHV-1 (H6b mutant)	Vaccine vs. placebo	Clinical signs: NSD. Mean RT: ↓ @ 4 dpi (Vx = 38.8; CON = 39.5°C) and 6 dpi (Vx = 38.5; CON = 39.2°C). Duration of pyrexia: NSD. Mean PBL number: ↑ @ 7 (7 dpi: Vx = 10100; CON = 7360 leukocytes/μl) to 10 dpi (10 dpi: Vx = 12500; CON = 9990 neutrophils/μl). Mean neutrophil count: ↑ @ 7-8 dpi (7dpi: Vx = 4730; CON = 2400 neutrophils/μl). Incidence of nasal shedding: NSD (Vx = 8/8; CON = 4/4). Duration of nasal shedding ↓ (Vx = 2.0 ± 0.6 d; CON = 4.8 ± 0.8 d). SN titer: ↑ @ 14, 21, and 35 dpi.	Experimental challenge. Small group sizes; Rx n =8; CON n = 4. Contact controls (n = 2) not included in data analysis. Data from all vaccinated horses was pooled. Pyrexia = RT > 39°C. No adverse effects reported. $p < 0.05$. Nasal shedding evaluated using plaque assay (Vero and BK cells).
Dolby et al., 1995	EHV-1, strain V592	Vaccine vs. unvaccinated	Incidence of clinical signs (nasal or ocular discharge): NSD (Vx = 0/5, CON = 0/3), pyrexia: NSD (Vx = 1/5, CON = 0/3), nasal shedding: Vx = 2/5; CON = 2/3, and viremia: ↓ (Vx = 0/5; CON = 3/3).	Experimental challenge. Small group sizes; Rx n =5; CON n = 3. No adverse effects reported. Freund's complete adjuvant (dose 1), Freund's Incomplete adjuvant (dose 2). Previous natural exposure(s) to EHV-1. Pyrexia = RT > 38.9°C. Statistical methods not reported. Post hoc analysis of incidence data performed ($p < 0.05$). Viremia and nasal shedding evaluated using plaque assay (RK13 cells).
Dutta, 1975	Rhinomune	None	Clinical (respiratory) signs on Farm 1 foals 3/25. Farm 1 abortion rate = 4/15; sick foals 4/15. SN titer ≥ 1:16 PF: 26/26. SN titer foals: no change.	Natural infection on Farm 1 – signs began 6 mo after 2nd vaccination. No statistical analysis.
Goehring et al., 2010	Rhinomune	Vaccine vs. unvaccinated	Incidence of pyrexia: NSD (Vx = 8/8; CON = 8/8). Average RT @ 1 dpi: NSD (Vx = 40.7; CON = 40.9°C); ↓@ 2 to 5 dpi (2 dpi: Vx = 39.3 ± 0.2; CON = 40.7 ± 0.1°C); and ↓ @ 7 dpi (Vx = 38.1 ± 0.4; CON = 39.6 ± 0.3°C). Clinical score ↓ ($p < 0.001$; e.g., 2 dpi: Vx = 2.2 ± 0.2; CON = 4.0 ± 0.3; 8 dpi: Vx = 0.2 ± 0.1; CON = 3.5 ± 0.6). Incidence of neurologic	Experimental challenge. Small group sizes (n =8/group). Adverse reactions reported: MLV = 1/8; Inactivated = 7/8 (after 2nd vaccination). Clinical score considered: cough, fever (RT > 38.6°C), nasal

			signs: NSD (Vx = 0/8; CON = 0/8). Incidence of viremia: NSD (Vx = 4/8; CON = 6/8). Viremia over time: NSD (p = 0.07). Amount and duration of nasal shedding ↓ (p < 0.001; e.g., @ 2 dpi Vx = 4.7; CON = 5.9 gB copies Log10; @ 8 dpi Vx = 0; CON = 3.2 gB copies Log10). SN titer ↑ (p < 0.001).	discharge, respiratory distress, anorexia and depression. Horses were also examined for neurologic signs (e.g., ataxia and weakness), but these were not detected in any horses. Ab subtype not analyzed statistically. Viremia and nasal shedding evaluated using qPCR
	Pneumabort-K	Vaccine vs. unvaccinated	Incidence of pyrexia: NSD (Vx = 8/8; CON = 8/8). Average RT: NSD. Clinical score ↓ (p < 0.001; e.g., 2 dpi: Vx = 2.0 ± 0.3; CON = 4.0 ± 0.3; 8 dpi: Vx = 1.7 ± 0.4; CON = 3.5 ± 0.6). Incidence of neurologic signs: NSD (Vx = 0/8; CON = 0/8). Incidence of viremia: NSD (Vx = 2/8; CON = 6/8). Viremia over time: ↓ (CON vs Rx likelihood of being viremic on any day; RR = 4.3, 95% CI = 1.2–14.3, p = 0.02). Amount and duration of nasal shedding ↓ (p < 0.001; e.g., @ 2 dpi Vx = 3.8; CON = 5.9 gB copies Log10; @ 12 dpi Vx = 0; CON = 1.7 gB copies Log10). SN titer ↑ (p < 0.001).	
		Rhinomune vs Pneumabort-K	Incidence of pyrexia: NSD (Vx = 8/8; CON = 8/8). Average RT ↓ Rhinomune (p < 0.001); odds of being febrile ↓ Rhinomune (OR = 3.0, 95%CI = 2.0–4.6; p < 0.001). Clinical score ↓ Rhinomune (p < 0.001). Nasal shedding NSD. Viremia NSD. SN titer ↓ Rhinomune (p < 0.0004).	
Goodman et al., 2006	Flu- vac Innovator 6	Vaccine vs. placebo	Incidence of minor swelling at injection site (Vx = 3/5). Average RT @ 2 dpi: ↓ (Vx = 39.0 ± 0.3°C, CON = 39.9 ± 0.5°C). Duration of pyrexia ↓ (Vx = 2.25 ± 0.5 d, CON = 3.4 ± 1.52 d; p < 0.01). Incidence of: clinical signs: NSD. Incidence of neurologic signs: NSD (Vx = 3/5, CON = 3/5). Incidence of viremia: NSD (Vx = 5/5; CON = 5/5). Incidence of nasal shedding: NSD (Vx = 5/5; CON = 5/5). Amount of nasal shedding @ 3 dpi: ↓ (Vx = 420; CON = 20500 viral titer). SN titer ↑ (p < 0.05). Serum IgG(T): NSD.	Experimental challenge. Small group sizes (n =5/group). Horses sedated prior to challenge. Post hoc analysis of incidence data. Pyrexia = RT ≥ 38.5°C. Viremia and nasal shedding evaluated using qPCR
	Rhinomune	Vaccine vs. placebo	Duration of pyrexia ↓ (Vx = 1.2 ± 0.6 d; CON = 3.4 ± 1.5 d; p < 0.01). Average RT @ 2 dpi: ↓ (Vx = 37.9 ± 0.4°C, CON = 39.9 ± 0.5°C); @ 3 dpi (Vx = 37.7 ± 0.3°C, CON = 38.7 ± 0.7°C); @ 7 dpi (Vx = 37.3 ± 0.3°C, CON = 37.7 ± 0.2°C). Incidence of clinical signs: NSD. Incidence of neurologic signs NSD (Vx = 0/5; CON = 3/5; p = 0.16). Incidence of viremia: NSD. (Vx = 5/5; CON = 5/5). Incidence of nasal shedding : NSD (Vx = 1/5; CON = 5/5; p =0.07). Amount of nasal shedding @ 3-5 dpi: ↓ (3 dpi: Vx = 0; CON = 20500 viral titer). SN titer ↑ (p < 0.05). Serum IgG(T): NSD.	
		Flu- vac vs Rhinomune	Average RT Rhinomune @ 7 dpi ↓(p < 0.05); clinical signs: NSD; SN titer Flu- vac ↑ after 1st vaccination (p < 0.05). Nasal shedding Rhinomune ↓ @ 3-5 dpi. Serum IgG(T): NSD. Viremia: NSD. Incidence of neurologic signs Rhinomune ↓ (p = 0.04).	
Hannant et al., 1993	Immune stimulating complex vaccine	Vaccine vs. unvaccinated	VN titer ↑after 2nd and 3rd vaccination (p < 0.05). Incidence of pyrexia @ 1 dpi: NSD (Vx = 7/9; CON = 6/6). Duration of pyrexia ↓ (1.6 ± 0.5 vs. 2.6 ± 0-8 d; p = 0.05). Incidence of clinical signs (conjunctivitis) @ 3-5 dpi: NSD (Vx = 5/9; CON = 4/6). Viremia: duration ↓ (Vx = 6.0 d; CON = 11.5 d; p < 0.02) and amount @ 10 and 12 dpi ↓ (Vx = 1.6, CON = 48.5 $TCID_{50}$; @ 10 dpi; p < 0.01). Incidence of nasal shedding @ 1 dpi: NSD (Vx = 9/9; CON = 6/6). Duration of nasal shedding: ↓ (Vx = 4 d; CON = 7.8 d; p < 0.01). Nasal viral amount ↓ (Vx = 13.6, CON = 28.6 $TCID_{50}$; p < 0.01).	Experimental challenge used nebulized virus – insufficient details provided to reconstruct exposure dose. Small group sizes (Rx n =9; CON = 6). Pyrexia = RT ≥ 39°C. Methods used to evaluate viremia and nasal shedding were not described.
Heldens et al., 2001	Duvaxyn EHV1,4	Foals Vaccine vs. unvaccinated	Number of days with pyrexia: NSD (Vx = 6.3; CON = 7.2 d). Mean RT @ 2 dpi ↓ (Rx =39.1; CON = 40.4°C, p < 0.005) and 6 dpi ↓ (Rx =38.9; CON = 39.5°C, p: p = ≤ 0.011). Duration of pyrexia: NSD (Vx = 6.3 d; CON =7.2). Mean total clinical score: NSD (Vx = 51.4 ± 17.8, CON = 68.9 ± 16.3). Incidence of viremia: NSD (Vx = 3/10, CON = 4/5; p = 0.06). Duration of viremia ↓ (Vx = 0.3 ± 0.5 d, CON = 2.2 ± 1.5 d; p = 0.03). Incidence of nasal shedding: NSD (Vx = 10/10; CON = 5/5). Mean nasal shedding @2-14 dpi: ↓ (Vx = 0.70 ± 0.92; CON = 1.83 ± 0.84 virus log_{10} titer/100 μl; p=0.014). Nasal shedding duration: ↓ (Vx = 5.2 ± 1.6, CON = 10 ± 3.2 d; p = 0.02). CF titer > 5 after 1st vaccination = 4/20; after 2nd = 19/20.	Experimental challenge. Small group sizes (Foals Rx n =10; CON = 5; PF: Rx n =5; CON = 4). Pyrexia = RT ≥ 38.8°C. Clinical score considered demeanor, appetite, nasal and ocular discharge, cough, and lymph node swelling Study also included animals challenged with EHV-4 (data not included). Viremia and nasal shedding evaluated using plaque assay (RK13 cells).
		PF Vaccine vs. unvaccinated	Incidence of pyrexia: NSD (Vx = 4/5, CON = 2/4). Duration pyrexia: NSD (Vx = 1.2 d; CON = 1.5). Incidence of nasal discharge: NSD (Vx = 4/5, CON = 2/4). Duration of nasal discharge: ↓ (Vx = 4.4; CON = 6.0 d). Incidence of lymphadenopathy: NSD (Vx = 4/5, CON = 3/4). Duration of lymphadenopathy: (Vx = 1.8; CON = 2.0 d). Incidence of ataxia:	

			NSD (Vx = 0/5, CON = 1/4). Incidence of abortion: NSD (Vx = 1/5, CON = 4/4; p = 0.048). Incidence of viremia: NSD (Vx = 5/5, CON = 4/4). Duration of viremia: NSD (Vx = 4.0, CON = 4.3 d). Incidence of nasal shedding: NSD (Vx = 5/5, CON = 4/4). Mean nasal titer @ 3 dpi: ↓ (Vx = 0.95, CON = 3.63 TCID$_{50}$/ml; p < 0.05). Duration of nasal shedding: NSD (Vx = 4.4; CON = 6.0 d). CF titer @ 2-20 wk after 1st vaccination ↑.	
Kydd et al., 2003	Duvaxyn EHV-1/4	Vaccine vs. unvaccinated	Duration of: pyrexia NSD (Vx = 1.2 ± 0.8 d; CON =1.8 ± 1.3). Clinical signs (ataxia): not reported. Incidence of abortion: ↓ (Vx = 1/5, CON = 9/9; p < 0.01). Duration of viremia: NSD (Vx = 4.0 ± 1.2 d; CON =4.3 ± 1.9 d). Duration of nasal shedding: ↓ (Vx = 2.2 ± 1.1 d; CON = 3.6 ± 1.1). Level of nasal shedding: ↓ (data not reported). Difference in cytotoxic T lymphocytes number pre- and post-infection: NSD. CF titer after 3rd vaccination ↑.	Experimental challenge. Small group sizes (PF Rx n =5; CON = 9). A third group (n =3) PF had multiple EHV-1 infections (data not included). Exact timing of challenge relative to vaccination is NR (mares challenged between 32 -42 weeks of gestation (last vaccination given at 36 wk of gestation). Companion study to Heldens et al., 2001. Post hoc analysis of incidence data performed (p < 0.05). Viremia and nasal shedding evaluated using plaque assay (RK13 cells).
Kydd et al., 2020	EHV-1 gM deletion mutant (RacHDgM)	Vaccine vs. unvaccinated	Incidence of pyrexia @ 2 dpi: NSD (Vx = 4/6; CON = 6/6). Mean RT @ 2-8 dpi ↓ (p < 0.001 – e.g., 1 dpi Vx = 38.8 ± 1.2; CON = 40.6 ± 0.4). Duration of pyrexia ↓ (Vx = 1.2 ± 1 d; CON = 4.5 ± 1 d; p = 0.004). Mean clinical scores ↓ (Vx = 8.3 ± 6.2, CON = 21.2 ± 7.8, p = 0.03). Incidence of viremia @ 5-7 dpi: NSD (Vx = 100%, CON = 100%). Duration of viremia ↓ (Vx = 2.5 ± 0.5 d, CON = 6.2 ± 1.0 d; p < 0.002). Last day of viremia: ↓ (Vx = day 8.3 ± 2.1 d, CON = day 15.3 ± 3.9; p=0.006). Mean nasal titer ↓ (e.g., @ 2 dpi VX = $10^{2.67}$/ml; CON = $10^{4.93}$/ml). Duration of nasal shedding ↓ (Vx = 2.8 ± 2.3 d, CON = 8.3 ± 1.6 d; p < 0.01). Nasal IgG and IgA: NSD. CTL activity: NSD. CF titer 2 wk after 2nd vaccination and 1 wk after challenge ↑ (p < 0.001). Mean VN antibody titers 2 wk after 2nd vaccination ↑ (p < 0.001); after challenge: NSD.	Experimental challenge. Small group sizes (PF Rx n =6; CON = 6). No adverse effects reported. Clinical score considered nasal or ocular discharge and lymphadenopathy. Pyrexia = RT ≥ 38.9°C. Viremia and nasal shedding evaluated using plaque assay (RK13 cells).
Matsumura et al., 1996	EHV1 strain KyA	Vaccine vs. unvaccinated	Incidence of pyrexia @ 2 dpi: NSD (Vx = 3/4, CON = 2/2). Mean RT @ 2 dpi: NSD (Vx = 39.4 ± 0.7; CON = 40.6 ± 0.2). Incidence of nasal discharge: NSD (Vx = 0/4, CON = 2/2). Incidence of lymphadenopathy: NSD (Vx = 0/4, CON = 2/2; p = 0.01). Incidence of viremia @ 1-25 dpi: NSD (Vx = 0/4, CON = 2/2). Duration of viremia: ↓ (Rx ≤ 6 d; CON ≤ 18-21 d). Incidence of nasal shedding @ 1-25 dpi: NSD (Vx = 1/4, CON = 2/2). Duration of nasal shedding: ↓ (Rx ≤ 3 d; CON ≤ 8 d). SN Ab titer ↑.	Experimental challenge. Small group sizes (Rx n =4; CON = 2). No statistical analysis performed. Post hoc analysis of incidence and RT data performed (p < 0.05). Viremia and nasal shedding evaluated using plaque assay (FHK and MDBK cells).
Minke et al., 2006	ALVAC-EHV	Vaccine vs. adjuvant	Incidence of pyrexia: NSD (Vx = 3/5, CON = 4/5). Duration of pyrexia: NSD (Vx = 0.6 d; CON = 1.4 d). Incidence of nasal discharge: NSD (Vx = 4/5, CON = 1/5). Duration of nasal discharge: NSD (Vx = 0.8 d; CON = 0.6 d). Incidence of viremia: NSD (Vx = 5/5, CON = 4/5). Duration of viremia: NSD. Incidence of nasal shedding: NSD (Vx = 5/5, CON = 5/5). AUC nasal shedding: NSD (Vx = 17.1; CON = 9.7). Duration of nasal shedding: NSD (Vx = 4.8 d; CON = 3.2 d). SN titer pre/post challenge: ↓ (pre; Vx = 1.9/3.2; CON = 2.5/3.0).	Experimental challenge. Small group sizes (Rx n =5; CON = 5). Pyrexia was undefined. Control for ALVAC trials was a killed, Carbopol adjuvanted whole virus vaccine containing EHV-1 and EHV-4. Control for plasmid trials was non-plasmid placebo. Viremia and nasal shedding evaluated using plaque assay (RK13 cells).
	ALVAC-EHV with Carbopol		Incidence of pyrexia: NSD (Vx = 5/5, CON = 4/5). Duration of pyrexia: NSD (Vx = 2.2 d; CON = 1.4 d). Incidence of nasal discharge: NSD (Vx = 1/5, CON = 1/5). Duration of nasal discharge: NSD (Vx = 0.2 d; CON = 0.6 d). Incidence of viremia: NSD (Vx = 5/5, CON = 4/5). Duration of viremia: NSD. Incidence of nasal shedding: NSD (Vx = 2/5, CON = 5/5). AUC nasal shedding: ↓ (Vx = 3.0; CON = 9.7). Duration of nasal shedding: ↓ (Vx = 1.0 d; CON = 3.2 d). SN titer pre/post challenge: NSD (pre; Vx = 2.6/3.1; CON = 2.5/3.0).	
	ALVAC-EIV with Carbopol		Incidence of pyrexia: NSD (Vx = 3/5, CON = 4/5). Duration of pyrexia: NSD (Vx = 0.8 d; CON = 1.4 d). Incidence of nasal discharge: NSD (Vx = 2/5, CON = 1/5). Duration of nasal discharge: NSD (Vx = 0.6 d; CON = 0.6 d). Incidence of viremia: NSD (Vx = 5/5, CON = 4/5). Duration of viremia: NSD. Incidence of nasal shedding: NSD (Vx = 5/5, CON = 5/5). AUC nasal shedding: NSD (Vx = 16.3; CON = 9.7). Duration of nasal shedding: NSD (Vx = 4.6 d; CON = 3.2 d). SN titer pre/post challenge: NSD (Vx = 1.6/3.1; CON = 2.5/3.0).	
	gB/gC/gD plasmids	Vaccine vs. unvaccinated	Incidence of pyrexia: NSD (Vx = 5/5, CON = 5/5). Duration of pyrexia: NSD (Vx = 3.2 d; CON = 3.0 d). Incidence of nasal discharge: NSD (Vx = 2/5, CON = 4/5). Duration of nasal discharge: NSD (Vx = 1.0 d; CON = 2.4 d). Incidence of viremia: NSD (Vx = 5/5, CON =	

			5/5). Duration of viremia: NSD. Incidence of nasal shedding: NSD (Vx = 4/5, CON = 5/5). AUC nasal shedding: NSD (Vx = 10.4; CON = 16.1). Duration of nasal shedding: NSD (Vx = 4.0 d; CON = 5.4 d). SN titer pre/post challenge: NSD (Vx = 0.9/2.6; CON = 0.9/2.6).	
	gB/gC/gD plasmids and $Al(PO)_4$		Incidence of pyrexia: NSD (Vx = 5/5, CON = 5/5). Duration of pyrexia: NSD (Vx = 2.8 d; CON = 3.0 d). Incidence of nasal discharge: NSD (Vx = 3/5, CON = 4/5). Duration of nasal discharge: NSD (Vx = 1.8 d; CON = 2.4 d). Incidence of viremia: NSD (Vx = 5/5, CON = 5/5). Duration of viremia: NSD. Incidence of nasal shedding: NSD (Vx = 4/5, CON = 5/5). AUC nasal shedding: NSD (Vx = 3.9; CON = 16.1). Duration of nasal shedding: NSD (Vx = 1.4 d; CON = 5.4 d). SN titer pre/post challenge: NSD (Vx = 1.1/2.6; CON = 0.9/2.6).	
	gB/gC/gD plasmids and Carbopol		Incidence of pyrexia: NSD (Vx = 5/5, CON = 5/5). Duration of pyrexia: NSD (Vx = 3.6 d; CON = 3.0 d). Incidence of nasal discharge: NSD (Vx = 3/5, CON = 4/5). Duration of nasal discharge: NSD (Vx = 1.4 d; CON = 2.4 d). Incidence of viremia: NSD (Vx = 5/5, CON = 5/5). Duration of viremia: NSD. Incidence of nasal shedding: NSD (Vx = 5/5, CON = 5/5). AUC nasal shedding: NSD (Vx = 8.6; CON = 16.1). Duration of nasal shedding: NSD (Vx = 3.2 d; CON = 5.4 d). SN titer pre/post challenge: NSD (Vx = 1.3/2.8; CON = 0.9/2.6).	
	Plasmids gB, gC, and gD and DMRIE-DOPE		Incidence of pyrexia: NSD (Vx = 5/5, CON = 5/5). Duration of pyrexia: NSD (Vx = 2.2 d; CON = 3.0 d). Incidence of nasal discharge: NSD (Vx = 3/5, CON = 4/5). Duration of nasal discharge: NSD (Vx = 0.6 d; CON = 2.4 d). Incidence of viremia: NSD (Vx = 4/5, CON = 5/5). Duration of viremia: NSD. Incidence of nasal shedding: NSD (Vx = 4/5, CON = 5/5). AUC nasal shedding: NSD (Vx = 7.4; CON = 16.1). Duration of nasal shedding: NSD (Vx = 3.0 d; CON = 5.4 d). SN titer pre/post challenge: ↓ (pre; Vx = 1.5/2.6; CON = 0.9/2.6).	
Mitchell et al., 1978	Rhinomune	Vaccine vs. unvaccinated	Incidence of clinical signs: NSD (Vx = 2/4, CON = 0/2). Incidence of neurologic signs: NSD (Vx = 1/4, CON = 0/2). Incidence of abortion: NSD (Vx = 2/4, CON = 0/2). Mean peak SN titer: NSD (Vx = 160 $\pm$ 113; CON = 100 $\pm$ 85).	Experimental challenge. Small group sizes (Rx n =5; CON = 5). Two CON PF aborted prior to challenge – cause was undetermined. EHV1 not isolated from aborted fetuses. No statistical analysis. Clinical signs were undefined. Post hoc analysis of incidence and SN titer data performed ($p < 0.05$).
Mohd-Azmi et al., 2002	EHV-1 strain AB4p L particles	Vaccine (IM or IN) vs. unvaccinated	Incidence of pyrexia: NSD (RxL_{im} = 1/1; RxL_{iN} = 1/1; CON = 2/2). Mean RT @ 2 dpi: NSD (RxL_{im} = 40.3°C; RxL_{iN} = 40.2°C; CON = 40.2-40.4°C). Incidence of lymphadenopathy: NSD (RxL_{im} = 1/1; RxL_{iN} = 1/1; CON = 0/2). Incidence of viremia: NSD (RxL_{im} = 1/1; RxL_{iN} = 1/1; CON = 2/2). Duration of viremia: NSD (RxL_{im} = 4 d; RxL_{iN} = 4 d; CON = 7 d). Incidence of nasal shedding: NSD (RxL_{im} = 1/1; RxL_{iN} = 1/1; CON = 2/2). Duration of nasal shedding: NSD (RxL_{im} = 6 d; RxL_{iN} = 7 d; CON = 12->18 d). Incidence of positive CFT titer: NSD (RxL_{im} = 1/1; RxL_{iN} = 1/1; CON = 0/2).	Experimental challenge. Small group sizes (Rx n =1/group; CON = 2). No statistical analyses. Post hoc analysis of data performed ($p < 0.05$). Viremia and nasal shedding evaluated using plaque assay (RK13 cells).
	EHV-1 strain AB4p UV inactivated	Vaccine vs. unvaccinated	Incidence of pyrexia: NSD (Vx = 1/1; CON = 2/2). Mean RT @ 2 dpi: NSD (Rx= 39.6°C; CON = 40.2-40.4°C); incidence of lymphadenopathy: NSD (Rx= 1/1; CON = 0/2). Incidence of viremia: NSD (Vx = 1/1; CON = 2/2). Duration of viremia: NSD (Vx = 2 d; CON = 7 d). Incidence of nasal shedding: NSD (Vx = 1/1; CON = 2/2). Duration of nasal shedding: NSD (Vx = 1 d; CON = 12->18 d). Incidence of positive CFT titer: NSD (Rx= 1/1; CON = 0/2).	
Mumford and Bates, 1984	Pneumabort-K	2 doses<br><br>Vaccine vs. unvaccinated	Incidence of pyrexia: NSD (Vx = 7/15; CON = 10/13). Mean RT: NSD (Vx = 39.1°C; CON = 39.1°C). Duration of pyrexia: NSD (Vx = 1.7 d; CON = 1.6 d). Incidence of nasal discharge: NSD (Vx = 3/15; CON = 4/13). Incidence of lymphadenopathy: ↓ (Vx = 6/15; CON = 11/13; p = 0.03). Incidence of nasal shedding: NSD (Vx = 13/15; CON = 10/13). Duration of nasal shedding: NSD (Rx= 2.0 d; CON = 2.6 d). Ab rise: NSD (Vx = 10/15; CON = 11/13).	Experimental challenge. Moderate group sizes (Rx n =15/group; CON = 13). Pyrexia = RT $\geq$ 38.8°C. Post hoc analysis of incidence data performed ($p < 0.05$). Nasal shedding evaluated using plaque assay (equine fetal kidney cells)
		3 doses<br><br>Vaccine vs. unvaccinated	Incidence of pyrexia: ↓ (VX = 3/15; CON = 10/13, p = 0.01). Mean RT: NSD (Vx = 39.0°C; CON = 39.1°C). Duration of pyrexia: NSD (Vx = 1.0 d; CON = 1.6 d). Incidence of nasal discharge: NSD (Vx = 3/15; CON = 4/13; p = 0.04). Incidence of lymphadenopathy: ↓ (Vx = 7/15; CON = 11/13; p = 0.05). Incidence of nasal shedding: NSD (Vx = 10/15; CON = 10/13). Duration of nasal shedding: NSD (Vx = 1.3 d; CON = 2.6 d; p < 0.002). Ab rise ↓ (Vx = 0/15; CON = 11/13; p = 0.02).	

Paillot et al., 2006	vP1014 and carbomer-PD adjuvant	Various doses<br><br>Vaccine vs. placebo	Incidence of pyrexia: NSD (RX_{1dose} = 0/1; RX_{3doses} = 1/1; RX_{4doses} = 1/2; CON = 1/1); duration of pyrexia: ND (RX_{1dose} = 0 d; RX_{3doses} = 3 d; RX_{4doses} = 0-4 d; CON = 1 d); incidence of viremia: NSD (RX_{1dose} = 1/1; RX_{3doses} = 1/1; RX_{4doses} = 1/2; CON = 1/1); incidence of nasal shedding: NSD (RX_{1dose} = 0/1; RX_{3doses} = 0/1; RX_{4doses} = 1/2; CON = 1/1); CTL activity @ 21dpi: ND (RX_{1dose} = low; RX_{3doses} = low; RX_{4doses} = high; CON = low).	Experimental challenge. Small group sizes (Rx n =1-2/group; CON = 1). Pyrexia = RT ≥ 38.9°C. No statistical analysis. Post hoc analysis of pooled incidence data performed ($p < 0.05$). Viremia and nasal shedding evaluated using qPCR.
Patel et al., 2003a	EHV1, strain C147	Vaccinated 5–6 mo pre-challenge (3.4-4.1 months of gestation)<br><br>Vaccine vs. unvaccinated	Incidence of pyrexia: NSD (Vx = 3/5, CON = 6/6). Mean RT: NSD (Vx = 39.0 ± 0.8°C; CON = 39.1 ± 0.2°C). Duration of pyrexia: NSD (Vx = 1.7 ± 1.2 d; CON = 2.8 ± 1.2 d). Incidence of nasal discharge: NSD (Vx = 4/5; CON = 6/6). Duration of nasal discharge: ↓ (Vx = 1.8 ± 1.0 d; CON = 12.5 ± 2.9 d; $p < 0.01$). Incidence of ocular discharge ↓ (Rx =1/5; CON = 6/6; $p < 0.01$). Duration of ocular discharge: ↓ (Vx = 3 ± 0 d; CON = 9.8 ± 5.2 d; $p < 0.01$). Incidence of lymphadenopathy: NSD (Vx = 2/5; CON = 5/6). Duration of lymphadenopathy: ↓ (Vx = 5.5 ± 5.0 d; CON = 17 ± 2.2 d; $p < 0.01$). Incidence of abortion: ↓ (Vx = 1/5; CON = 6/6, $p < 0.01$). Incidence of viremia: ↓ (Vx = 2/5; CON = 6/6; p = 0.02). Duration of viremia: NSD (Vx = 2 ± 1.4 d; CON = 2.3 ± 0.5 d). Incidence of nasal shedding: NSD (Vx = 5/5; CON = 6/6). Mean nasal shedding: ↓ (Vx = 2.1; CON = 3.6 log_{10} $TCID_{50}$). Duration of nasal shedding: ↓ (Vx = 2.2 ± 1.1 d; CON = 6.5 ± 0.8 d; $p < 0.01$).	Experimental challenge. Small group sizes 5-6 mares/group. No significant clinical signs observed after vaccination. One mare vaccinated at 5–6 mo pre-challenge was removed due to a *S. zooepidemicus* infection. Pyrexia = RT ≥ 38.5°C. Mean values provided for durations. Post hoc analysis of data performed ($p < 0.05$). Viremia and nasal shedding evaluated using plaque assay (ED cells)
		Vaccinated 4 mo pre-challenge (5.4-5.8 months of gestation)<br><br>Vaccine vs. unvaccinated	Incidence of pyrexia: ↓ (Vx = 2/6, CON = 6/6; p = 0.01). Mean RT: NSD (Vx = 38.9 ± 0.1°C; CON = 39.1 ± 0.2°C). Duration of pyrexia: NSD (Vx = 2.0 ± 1.4 d; CON = 2.8 ± 1.2 days). Incidence of nasal discharge: NSD (Vx = 5/6; CON = 6/6). Duration of nasal discharge: ↓ (Vx = 2.3 ± 1.9 d; CON = 12.5 ± 2.9 d; $p < 0.01$). Incidence of ocular discharge ↓ (Rx =0/6; CON = 6/6; $p < 0.01$). Duration of ocular discharge: ↓ (Vx = 0 ± 0 d; CON = 9.8 ± 5.2 d; $p < 0.01$). Incidence of lymphadenopathy: NSD (Vx = 2/6; CON = 5/6). Duration of lymphadenopathy: ↓ (Vx = 4.5 ± 3.5 d; CON = 17 ± 2.2 d; $p < 0.01$). Incidence of abortion: ↓ (Vx = 1/6; CON = 6/6, $p < 0.01$). Incidence of viremia: ↓ (Vx = 3/6; CON = 6/6; p = 0.05). Duration of viremia: ↓ (Vx = 1 ± 0 d; CON = 2.3 ± 0.5 d; $p < 0.01$). Incidence of nasal shedding: NSD (Vx = 4/6; CON = 6/6). Mean nasal shedding: ↓ (Vx = 2.6; CON = 3.6 log_{10} $TCID_{50}$). Duration of nasal shedding: ↓ (Vx = 1.5 ± 1.3 d; CON = 6.5 ± 0.8 d; $p < 0.01$). Incidence of increased VN Ab: NSD (Vx = 3/5; CON = 6/6).	
Patel et al., 2003b	EHV1, strain C147	Vaccine vs. unvaccinated	Incidence of pyrexia ↓ (Vx = 0/8, CON = 7/8; p = 0.05). Duration of pyrexia ↓ (Vx = 0 ± 0 d, CON = 2.9 ± 1.8 d). Incidence of ocular discharge NSD (Vx = 1/8, CON = 2/8). Incidence of serous/mucopurulent discharge: NSD (Vx = 1/8, CON = 6/8). Duration of serous/mucopurulent discharge: ↓ (Vx = 0.13 ± 0.35 d, CON = 2.6 ± 2.1 d; p = 0.006). Incidence of viremia: ↓ (Vx = 0/8, CON = 8/8; p = 0.04). Duration of viremia: ↓ (Vx = 0 ± 0 d, CON = 2.9 ± 1.0 d). Incidence of nasal shedding: NSD (Vx = 6/8, CON = 8/8). Peak nasal shedding: ↓ (Vx = 1.4 ± 0.8; CON = 2.6 ± 0.4 log_{10} $TCID_{50}$; $p < 0.01$). Duration of nasal shedding: ↓ (Vx = 1.5 ± 0.5 d CON = 4.5 ± 0.8 d; $p < 0.01$). VN antibody titer ↑ @ 6 wk after Rx. Mean peak nasal titer @ 1-14 dpi ↓ (Vx = 1.4 ± 0.3 $TCID_{50}$/2ml, CON = 2.6 ± 0.4 $TCID_{50}$/2ml; $p < 0.01$.	Experimental challenge. Small group sizes (Rx n =8; CON = 8). No adverse effects reported. Vaccination associated with nasal viral shedding and viremia (pre-challenge). Pyrexia = RT ≥ 38.5°C. Study also included animals challenged with EHV-4 (data not included). Statistical methods not provided. Post hoc analysis of data performed ($p < 0.05$). Viremia and nasal shedding evaluated using plaque assay (ED cells)
Patel et al., 2004	EHV1, strain C147	Vaccine vs. unvaccinated	Incidence of pyrexia: ↓ (Vx = 2/8, CON= 6/6). Duration of pyrexia: ↓ (Vx = 1.6 ± 0.5 days; CON = 5.0 ± 0.7 days). Incidence of nasal discharge: NSD (Rx= 8/8, CON = 6/6). Severity of nasal discharge: ↓ (Vx = 7.0 ± 3.4; 18.7 ± 5.0) Duration of nasal discharge: ↓ (Rx= 6.8 ± 3.3 d; CON= 11.0 ± 1.7 d, p = 0.013). Incidence of ocular discharge: NSD (Rx= 2/8, CON = 3/6). Duration of ocular discharge: NSD (Vx = 0.9 ± 2.1 d, CON = 2.2 ± 3.1 d). Incidence of viremia: ↓ (Vx = 4/8, CON = 6/6). Duration of viremia: ↓ (Vx = 0.63 ± 0.7 d; CON = 2.3 ± 1.5 d). Incidence of viral shedding @ 8 wk after challenge: NSD (Vx = 7/8, CON = 6/6). Duration of viral shedding: ↓ (Vx = 2.0 ± 2.5 d, CON = 7.7 ± 2.9 d). Mean nasal shedding: ↓ (Vx = 2.0 log_{10} $TCID_{50}$/2mL, CON = 2.8 log_{10} $TCID_{50}$/2mL, p = 0.008).<br><br>Severity of nasal discharge: ↓ (Rx= 7.0 ± 3.4, CON= 18.7 ± 5.0, p= 0.0024). Severity of ocular discharge: NSD (Rx= 1.8 ± 4.2, CON= 4.3 ± 6.2).	Experimental challenge. Small Rx group sizes (Vx = 8; CON = 6). Pyrexia = RT > 38.8°. No adverse effects reported. p ≤ 0.05. Viremia and nasal shedding evaluated using plaque assay (ED cells)

Perkins et al., 2019	EHV-1 Strain Ab4	Vaccine vs. unvaccinated	Incidence of pyrexia: NSD (Vx = 0/5; CON = 5/5; p = 0.08). Median RT @ 2, 2.5, and 5.5 dpi: ↓ (Vx = 37.9°C; CON = 40.0°C). Duration of pyrexia: ND (Vx = 0 d; CON > 3 d). Incidence of clinical signs: NSD (Vx = 0/5; CON = 5/5; p = 0.08). Median clinical score @ 3 dpi: ↓ (Vx = 2, CON = 5). Duration of clinical signs: ND (Vx = 0 d; CON ~ 3 d). Incidence of neurologic signs: NSD (Vx = 0/5; CON = 0/5). Incidence of viremia: NSD (Vx = 0/5; CON = 5/5). Median viremia (CT value) @ 5 dpi: ↓ (Vx = 40.0; CON = 32.1). Duration of viremia: ↓ (Vx = 0 d; CON > 3 d). Incidence of nasal shedding @ 3 dpi: NSD (Vx = 0/5; CON = 5/5; p = 0.08). Median nasal shedding @ 3 dpi: ↓ (Vx = 0; CON = 2 X 10^3). Duration of nasal shedding: ↓ (Vx = 0 d; CON > 3 d).	Experimental challenge. Small Rx group sizes (Vx = 5; CON = 5). Vaccination protocol incompletely described. Additional results including inflammatory markers (IFN-a, CCL2, sCD14) and associations between nasal shedding and total and subtype (gB, gC, gD) Ab with clinical signs not included here. Administration of EHV-1 strains Ab4 and Ab4ΔORF1/71 were associated with pyrexia, respiratory signs, viremia, and Ab production. Pyrexia = RT > 38.5°C. Clinical score assessed nasal and ocular discharge, lymph node enlargement, ataxia and neurological signs, depression, and appetite. Neurologic signs not observed in any horses. p < 0.05. Viremia and nasal shedding evaluated using qPCR.
	EHV-1 Strain Ab4ΔORF1/71	Vaccine vs. unvaccinated	Incidence of pyrexia: NSD (Vx = 0/5; CON = 5/5; p = 0.08). Mean RT @ 2-2.5 dpi: ↓ (Vx = 38.0°C; CON = 40.0°C, p < 0.05). Duration of pyrexia: ND (Vx = 0 d; CON > 3 d). Incidence of clinical signs: NSD (Vx = 0/5; CON = 5/5; p = 0.08). Median clinical score @ 3 dpi: ↓ (Vx = 1.5, CON = 5). Duration of clinical signs: ND (Vx = 0 d; CON ~ 3 d). Incidence of neurologic signs: NSD (Vx = 0/5; CON = 0/5). Incidence of viremia: NSD (Vx = 2/5; CON = 5/5). Median viremia (CT value) @ 5 dpi: ↓ (Vx = 40.0; CON = 32.1). Duration of viremia: ↓ (Rx < 2 d; CON > 3 d). Incidence of nasal shedding @ 3 dpi: NSD (Vx = 0/5; CON = 5/5). Median nasal shedding @ 3 dpi: ↓ (Vx = 0; CON = 2 X 10^3). Duration of nasal shedding: ↓ (Vx = 0 d; CON > 3 d).	
Purdy et al., 1978a (mares)s	Rhinoquin	Experiment 1 (combined data for 1- or 2-doses)<br><br>Vaccine vs. unvaccinated	Incidence of pyrexia: ↓ (Vx = 0/17; CON = 12/12). Incidence of respiratory signs ↓ (Vx = 0/17; CON = 12/12). Incidence of leukopenia: ↓ (Vx = 0/17; CON = 12/12). Incidence of abortion: NSD (Rx =0 /14; CON = 1/4 EHV-1 isolated from aborted foal; p = 0.156). Incidence of early foal loss: NSD (Rx =1/14 –EHV1 not isolated from foal; CON = 1/3; p =0.223). Incidence of viremia: ↓ (Vx = 1/17; CON = 12/12). Incidence of nasal shedding: ↓ (Vx = 0/17; CON = 12/12).	Experimental challenge; multiple sub-experiments performed that shared controls; small group sizes (experiment # 1: Rx_{1dose} = 10 [PM = 8]; Rx_{2dose} = 10 [PM = 9]; CON = 12 [PM = 3]; experiment # 2: Rx_{1dose} = 9 [PM = 0]; Rx_{2dose} = 5 [PM = 4]; CON = 3 [PM = 0]. Contact controls also used (data pooled with other controls). Challenge for experiments 1 and 2 were 40-50 d or 1 yr after vaccination; respectively. Some CON groups included foals (experiment 1 n = 2; experiment 2 n = 1). PF vaccinated at various times before parturition (experiment 1: 3-123 d; mean = 72 d). Statistical analyses were not reported. No adverse effects reported. Some Rx horses (n = 3) in experiment #1 had *S. equi* or *S. zooepidemicus* infections and were excluded from original analysis. Fever undefined. Post hoc analysis of incidence data performed (p < 0.05). Methods used to evaluate viremia and nasal shedding were not described.
		Experiment 2 (combined data for 1- or 2-doses)<br><br>Vaccine vs. unvaccinated	Incidence of pyrexia: ↓ (Vx = 0/14; CON = 3/3). Mean level of leukopenia: ↓ (decline Vx = 1200/mm^3, CON = 7900/mm^3). Incidence of abortion: ↓ (Vx = 0/4, CON = 1/1 – EHV1 isolated from CON aborted foal). Incidence of viremia: NSD (Vx = 14/14; CON = 3/3). Incidence of nasal shedding: ↓ (Vx = 1/14; CON = 3/3). SN titer: NSD.	
Purdy et al., 1978b (foals)	Rhinoquin	Vaccine vs. unvaccinated	Experiment 3. Incidence of pyrexia: Unknown (Vx = 0/5; CON = unknown). Mean RT @ 4 dpi: ↓ (Vx = 38.7°C; CON = 39.9°C). Incidence of respiratory signs: ↓ (Vx = 0/5; CON = 3/3). Mean WBC count @ 5 dpi: ↑ (Vx = 10400; CON = 7000 cells/mm^3). Incidence of viremia: ↓ (positive samples Vx = 28%; CON = 72%). Incidence of nasal shedding: ↓ (positive samples Vx = 23%; CON = 44%).	Experimental challenge. Single vaccination. Interval between vaccination and challenge varied. Experiments #3, #4, and #5 occurred 84 d, 84-172 d, or 1 year after vaccination, respectively. Pooled data across individual experiments – each had one or more treatment conditions (e.g., foal age; time to challenge). Statistical methods were not reported. No adverse effects reported. Data from some experiments (e.g., experiments 1, 2) are not included (lack vaccination and/or viral challenge). Some vaccinated horses (n = 3) in experiment #4 had *S. zooepidemicus* infections. Post hoc analysis of pooled
		Vaccine vs. unvaccinated	Experiment 4. Incidence of pyrexia: ↓ (Vx = 0/31; CON = 5/5). Mean RT: ↓ (Rx < 39; CON = 39.9°C). Incidence of respiratory signs: ↓ (Vx = 0/31; CON = 5/5). Mean WBC count @ 5dpi: ↑ (Vx = 11,000-12,000; CON < 8500 cells/mm^3). Percent positive viremia and nasal shedding: ↓ (unable to calculate incidence). SN titer (NSD).	

		Vaccine vs. unvaccinated	Experiment 5. Foals vaccinated at 1-12 days of age challenged at 1 year. Incidence of pyrexia and leukopenia: NSD (Vx = 12/12; CON = 4/4). Mean RT @ 4 dpi: NSD (Vx = 40.3°C; CON = 39.8°C). Mean WBC count @ 5 dpi: NSD (Vx = 8950; CON = 10100 cells/mm^3). Incidence of viremia and nasal shedding: NSD (Vx = 12/12; CON = 4/4).<br><br>Experiment 5. Foals vaccinated at 7-42 days of age challenged at 1 year. Incidence of pyrexia and leukopenia: Unknown. Mean RT @ 5 dpi: NSD (Vx = 41.1°C; CON = 38.6°C). Mean WBC count @ 5 dpi: NSD (Vx = 10300; CON = 7260 cells/mm^3). Incidence of viremia and nasal shedding: NSD (Vx = 6/7; CON = 4/4).<br><br>Experiment 5. Foals vaccinated at 122 days of age challenged at 1 year. Incidence of pyrexia, respiratory signs, and leukopenia: ↓ (Vx = 0/7; CON = 4/4). Incidence of viremia: ↓ (Vx = 0/7; CON = 4/4). Incidence of nasal shedding: NSD (Vx = 0/7; CON = 2/4).	incidence data performed ($p < 0.05$). Methods used to evaluate viremia and nasal shedding were not described.
Schnabel et al., 2019	EHV1 strain Ab4/8	Vaccine vs. unvaccinated	Incidence of pyrexia: ↓ (Vx = 0/8; CON = 8/8). Mean RT @1.5-3.5 dpi: ↓ (@ 2 dpi: Vx = 37.5; CON = 40.9°C). Duration of pyrexia: ↓ (Vx = < 1 d; CON = 3 d). Incidence of respiratory signs: ↓ (Vx = 0/8; CON = 8/8). Mean clinical score @ 2-6 dpi: ↓ (@ 4 dpi: Vx = 2.2 ± 0.4, CON = 5.6 ± 0.7). Incidence of ataxia: NSD (Vx = 0/8; CON = 1/8). Incidence of viremia: ↓ (Vx = 3/8; CON = 8/8). Mean maximum viremia: ↓ (Vx = 12.9 ± 17.9; CON = 32.2 ± 1.8 cycle threshold). Mean duration of viremia: ↓ (Vx = 2.6 ± 3.7 d; CON = 6.3 ± 1.4 d). Incidence of nasal shedding: ↓ (Vx = 3/8; CON = 8/8). Mean maximal nasal shedding: ↓ (Vx = 384 ± 1020; CON = 136000 ± 24000 PFU/ml). Duration of nasal shedding: ↓ (Rx ~ 2 d; CON ~ 6 d).	Experimental challenge. Small Rx group sizes (Vx = 8/group; CON = 8). Additional group (n = 8) infected with Pyrexia = RT >38.5°C. Clinical score considered nasal discharge, ocular discharge, lymph node enlargement, ataxia, depression, and reduced appetite. Vaccination with Ab4ΔORF2 was associated with initial reduced fever, mild respiratory disease, viremia, and nasal shedding. Additional Ab and functional assays performed (data not reported here). $p \leq 0.05$. Viremia and nasal shedding evaluated using qPCR.
	EHV1 strain Ab4ΔORF2	Vaccine vs. unvaccinated	Incidence of pyrexia: ↓ (Vx = 1/8; CON = 8/8). Mean RT @1.5-3.5 dpi: ↓ (@ 2 dpi: Vx = 37.5; CON = 40.9°C). Duration of pyrexia: ↓ (Vx = < 1 d; CON = 3 d). Incidence of respiratory signs: ↓ (Vx = 0/8; CON = 8/8). Mean clinical score @ 2-6 dpi: ↓ (@ 4 dpi: Vx = 2.9 ± 0.3, CON = 5.6 ± 0.7). Incidence of ataxia: NSD (Vx = 0/8; CON = 1/8). Incidence of viremia: ↓ (Vx = 1/8; CON = 8/8). Mean maximum viremia: ↓ (Vx = 4.6 ± 13.1; CON = 32.2 ± 1.8 cycle threshold). Mean duration of viremia: ↓ (Vx = 1.0 ± 2.8 d; CON = 6.3 ± 1.4 d). Incidence of nasal shedding: ↓ (Vx = 1/8; CON = 8/8). Mean maximal nasal shedding: ↓ (Vx = 1.3 ± 3.5; CON = 136000 ± 24000 PFU/ml). Duration of nasal shedding: ↓ (Rx ~ 0 d; CON ~ 6 d).	
Soboll et al., 2006	gB/gC/gD plasmids	Vaccine vs. unvaccinated	Incidence of pyrexia @ 1 dpi: NSD (Vx = 4/5; CON = 5/5). Mean RT @ 1 dpi: NSD (Vx = 39.4 ± 1.2; CON = 39.4 ± 0.7). Incidence of nasal discharge: NSD (Vx = 5/5; CON = 5/5). Incidence of severe nasal discharge: NSD (Vx = 1/5; CON = 0/5). Duration of nasal discharge (days observed): NSD (Vx = 7 d; CON = 7 d). Incidence of viremia: NSD (Vx = 4/5; CON = 1/5). Incidence of nasal shedding: NSD (Vx = 4/5; CON = 4/5). VN titer after challenge: NSD.	Experimental challenge. Small Rx group sizes (Vx = 5/group; CON = 5). No adverse effects reported. Post hoc analysis: pyrexia = RT >38.3°C. Additional Ab and functional assays performed (data not reported here). $p \leq 0.05$. Viremia and nasal shedding evaluated using plaque assay (ED cells).
	IE/UL5 plasmids	Vaccine vs. unvaccinated	Incidence of pyrexia @ 1 dpi: NSD (Vx = 5/5; CON = 5/5). Mean RT @ 1 dpi: NSD (Vx = 39.7 ± 0.3; CON = 39.4 ± 0.7). Incidence of nasal discharge: NSD (Vx = 5/5; CON = 5/5). Incidence of severe nasal discharge: NSD (Rx_I = 1/5; CON = 0/5). Duration of nasal discharge (days observed): NSD (Vx = 3 d; CON = 7 d). Incidence of viremia: NSD (Vx = 4/5; CON = 1/5). Incidence of nasal shedding: NSD (Vx = 4/5; CON = 4/5). VN titer after challenge: NSD.	
Soboll et al., 2010	rMVA-IE<br>(A3/B2 vaccinates)	CON	Clinical score: ↓ (p = 0.0003). Neurologic signs incidence: NSD (Vx = 0/10; CON = 0/5). Incidence of viremia: ↓ (Rx= 1/10; CON = 5/5; p = 0.031). Incidence of nasal shedding: NSD (Vx = 10/10; CON = 5/5). Duration of nasal shedding: NSD. VN titer after challenge: NSD.	Experimental challenge. Horses with either the MHC-I A3/B2 haplotype (A3/B2 vaccinates) or a different MHC I haplotype (non-A3 vaccinates or A3-non-B2 vaccinates) were used. Controls had a haplotype other than A3. Small group sizes ($Rx_{A3/B2}$ = 10; $Rx_{non\text{-}A3}$ = 6; $Rx_{A3non\text{-}A2}$ = 5; CON = 5). Pyrexia = RT >38.6°C. Clinical score considered presence of cough, fever ocular or nasal discharge and depression. Additional
	rMVA-IE<br>(non-A3 vaccinates)		Clinical score: NSD. Neurologic signs incidence: NSD (Vx = 0/6; CON = 0/5). Incidence of viremia: NSD (Vx = 3/6; CON = 5/5). Incidence of nasal shedding: NSD (Vx = 6/6; CON = 5/5). Duration of nasal shedding: NSD. VN titer after challenge: NSD.	

	rMVA-IE (A3-non-B2 vaccinates)		Clinical score: NSD. Neurologic signs incidence: NSD (Vx = 0/5; CON = 0/5). Incidence of viremia: NSD (Vx = 5/5; CON = 5/5). Incidence of nasal shedding: NSD (Vx = 5/5; CON = 5/5). Duration of nasal shedding: NSD. VN titer after challenge: NSD.	Ab and functional assays performed (data not reported in table). Viremia and nasal shedding evaluated using qPCR.
Thomson et al., 1979	Inactivated RAC-H and alhydrogel	Vaccine vs. placebo	Foals: Incidence of pyrexia: NSD (Vx = 2/4; CON = 6/8). Incidence of clinical signs (nasal discharge): NSD (Vx = 1/4; CON = 4/8). Incidence of nasal shedding: NSD (Vx = 4/4; CON = 7/8). Incidence of 4-fold ↑ VN titer: NSD (Vx = 2/4; CON = 8/8). Yearlings: Incidence of pyrexia: NSD (Vx = 1/4; CON = 5/9). Incidence of nasal shedding: NSD (Vx = 1/4; CON = 6/9). Incidence of 4-fold ↑ VN titer: NSD (Vx = 0/4; CON = 5/9).	Experimental challenge. Small group sizes (Rx n =3-4/group; CON = 4-5/group). Pyrexia = RT ≥ 38.5°C. Data from both controls (no vaccination, PBS as adjuvant control) pooled. All the yearlings suffered Mucopurulent nasal discharges seen in yearlings throughout the vaccination period. Post hoc analysis of incidence data performed ($p < 0.05$). Methods used to nasal shedding were not described.
	Inactivated RAC-H and adjuvant 65		Foals: Incidence of pyrexia: NSD (Vx = 2/4; CON = 6/8). Incidence of nasal shedding: NSD (Vx = 4/4; CON = 7/8). Incidence of 4-fold ↑ VN titer: NSD (Vx = 3/4; CON = 8/8). Yearlings: Incidence of pyrexia: NSD (Vx = 1/4; CON = 5/9). Incidence of nasal shedding: NSD (Vx = 3/4; CON = 6/9). Incidence of 4-fold ↑ VN titer: NSD (Vx = 0/4; CON = 5/9).	
	Inactivated RAC-H and levamisole		Foals: Incidence of pyrexia: NSD (Vx = 3/4; CON = 6/8). Incidence of clinical signs (nasal discharge): NSD (Vx = 2/4; CON = 4/8). Incidence of nasal shedding: NSD (Vx = 3/4; CON = 7/8). Incidence of 4-fold ↑ VN titer: NSD (Vx = 4/4; CON = 8/8).	
	Inactivated RAC-H and *C. parvum*		Yearlings: Incidence of pyrexia: NSD (Vx = 2/4; CON = 5/9). Incidence of nasal shedding: NSD (Vx = 1/4; CON = 6/9). Incidence of 4-fold ↑ VN titer: NSD (Vx = 0/4; CON = 5/9).	
	Inactivated RAC-H and Freund's complete adjuvant		Yearlings: Incidence of pyrexia: NSD (Vx = 1/3; CON = 5/9). Incidence of nasal shedding: NSD (Vx = 1/3; CON = 6/9). Incidence of 4-fold ↑ VN titer: NSD (Vx = 0/3; CON = 5/9).	
Traub-Dargatz et al., 2013	NR	Various vaccination protocols ((in year preceding exposure vs vaccinated 35d preceding event vs vaccinated 36-70d pre exposure vs vaccinated 71-100d pre exposure.	3.3x greater odds of EHM if EHV-1 vaccination in 5 weeks before infectious event, 1.9x greater odds with each increase in number of EHV-1 vaccinations in the year before the infectious event. Multivariable model: ↑ risk of EHM if vaccinated in 5 weeks prior to infectious event (OR: 7.12; 95% CI: 1.48, 34.17)	Natural infections. No information provided about vaccines used.
Tsujimura et al., 2009	(delta)gE EHV-1 strain	Lower dose vaccine (10^5 PFU)<br><br>Vaccine vs. revertant	Incidence of pyrexia @ 1 dpi: NSD (Vx = 3/3; CON = 3/3). Mean RT: ↓ @ 3-6 dpi (2 dpi: Vx = 38.6 ± 0.4°C; CON = 39.7 ± 0.2°C). Duration of pyrexia: NR (Vx = 1 d; CON = 2-3 d). Incidence of mucopurulent nasal discharge: NSD (Vx = 2/3; CON = 3/3). Mean nasal discharge score: ↓ (Vx = 4.67 ± 2.31; CON = 11.3 ± 4.16). Incidence of lymphadenopathy: NSD (Vx = 3/3; CON = 3/3). Mean lymph node score: NSD. Incidence of viremia @ 6 dpi: NSD (Vx = 2/3, CON = 3/3). Level of viremia @ 5, 7, 10, and 12 dpi: ↓ (e.g., @ 7 dpi Vx = 1.2 ± 1.0; CON = 2.6 ± 0.4 mean log10 DNA copies). Incidence of nasal shedding: NSD (Vx = 3/3, CON = 3/3). Mean level of nasal shedding @ 6-7 and 10-14 dpi: ↓ (e.g., @ 7 dpi Vx = 5.2 ± 0.4; CON = 6.9 ± 0.6 mean log10 DNA copies). SN titers: NSD.	Experimental challenge. Two different vaccine doses (10^5 or 10^6 PFU of ΔgE). Small group sizes (Rx n =8; CON = 4). Pyrexia = RT ≥ 39.0°C. $p < 0.05$. No adverse effects seen in foals given the ΔgE vaccine. Additional experimental groups without viral challenge were performed (data not included here). Post hoc analysis of incidence data performed ($p < 0.05$). Viremia and nasal shedding evaluated using qPCR.
		Lower dose vaccine (10^6 PFU)<br><br>Vaccine vs. revertant	Incidence of pyrexia @ 1 dpi:NSD (Vx = 2/3; CON = 3/3). Mean RT: ↓ @ 3-6 dpi (2 dpi: Vx = 38.3 ± 0.2°C; CON = 39.7 ± 0.2°C). Duration of pyrexia: NR (Vx = 1 d; CON = 2-3 d). Incidence of mucopurulent nasal discharge: NSD (Vx = 1/3; CON = 3/3). Mean nasal discharge score: ↓ (Vx = 1.67 ± 2.08; CON = 11.3 ± 4.16). Incidence of lymphadenopathy: NSD (Vx = 2/3; CON = 3/3). Mean lymph node score: NSD. Incidence of viremia @ 6 dpi: NSD (Vx = 3/3, CON = 3/3). Level of viremia @ 10 and 12 dpi: ↓ (e.g., @ 10 dpi Vx = 0.5 ± 0.8; CON = 2.1 ± 0.2 mean log10 DNA copies). Incidence of nasal shedding: NSD (Vx = 3/3, CON = 3/3). Mean level of nasal shedding @ 6-14 dpi: ↓. SN titers: NSD.	

Van de Walle et al., 2009	rNY03(delta)IR6/1gp2S	Vaccine vs. placebo	Incidence of pyrexia @ 1 dpi: ↓ (Vx = 0/8; CON = 4/4). Mean RT @ 1 dpi: ↓ (Vx = 37.9 ± 0.4°C; CON = 39.3 ± 0.6°C). Incidence of neurologic signs (ataxia): NSD (Vx = 0/8; CON = 1/4). Incidence of viremia: ↓ (Vx = 0/8; CON = 4/4). Mean level of viremia: ↓. Duration of viremia: ↓. Incidence of nasal shedding: NSD (Vx = 8/8; CON = 4/4). Mean nasal shedding: ↓ (@ 3dpi: Vx = 6.7 X 10^5; CON = 2.4 X 10^7). Duration of nasal shedding: ↓ Rx ~ 10 d; CON > 14 d). SN titer ↑.	Experimental challenge. Small group sizes (Rx n =8; CON = 4). Pyrexia = RT ≥ 39.0°C. Post hoc analysis of incidence data performed ($p < 0.05$). Viremia and nasal shedding evaluated using qPCR.
Wagner et al., 2017	Streptavidin-conjugated gC/IL-4 fusion protein (Sav-gC/IL-4)	Vaccine vs. unvaccinated	Incidence of pyrexia @ 1 dpi: NSD (Vx = 5/5; CON = 5/5). Pyrexia severity @ 1.5 dpi: NSD (Vx = 40.5 ± 0.1°C; CON = 40.4 ± 0.1°C). Duration of pyrexia: NSD (Vx = 2 d; CON = 2 d). Incidence of nasal discharge: NSD (Vx = 5/5; CON = 5/5). Incidence of lymphadenopathy: NSD (Vx = 5/5; CON = 5/5). Clinical score: NSD. Incidence of neurologic signs: NSD (Vx = 0/5; CON = 0/5). Incidence of viremia and nasal shedding: NSD (Vx = 5/5; CON = 5/5). Mean level of viremia and nasal shedding: NSD. Serum Ab against gC and gD @ 10 dpi (Rx + IgE-bio and Rx vs CON).	Experimental challenge. Small group sizes (n =5/group). One vaccinated group also received IgE-bio at birth. Clinical score considered nasal and ocular discharge, lymphadenopathy, and neurologic signs. Pyrexia = RT >38.6°C. Cytokine changes and additional immunologic endpoints reported (data not shown). Viremia and nasal shedding evaluated using qPCR.
	Streptavidin-conjugated gC/IL-4 fusion protein (Sav-gC/IL-4) and IgE-bio	Vaccine vs. unvaccinated	Incidence of pyrexia @ 1 dpi: NSD (Vx = 5/5; CON = 5/5). Pyrexia severity @ 1.5 dpi: ↓ (Vx = 39.8 ± 0.2°C; CON = 40.4 ± 0.1°C). Duration of pyrexia: NSD (Vx = 2 d; CON = 2 d). Incidence of nasal discharge: NSD (Vx = 5/5; CON = 5/5). Incidence of lymphadenopathy: NSD (Vx = 5/5; CON = 5/5). Clinical score: NSD. Incidence of neurologic signs: NSD (Vx = 0/5; CON = 0/5). Incidence of viremia and nasal shedding: NSD (Vx = 5/5; CON = 5/5). Mean level of viremia and nasal shedding: NSD. Serum Ab against gC and gD @ 10 dpi (Rx + IgE-bio and Rx vs CON).	

Abbreviations: @: at; ↓: decreased; ↑: increased; -: negative; +: positive or observed; 1yo: yearling; 2yo: two-year-old; Ab: antibody; ALVAC-EHV: recombinant canarypox virus (vCP132) expressing the gB, gC and gD glycoproteins of the Kentucky strain of EHV- 1; ALVAC-EIV: recombinant canarypox virus (vCP1502) expressing the haemagglutinin of influenza A/eq/Prague/56 (H7N7); AUC: area under the curve; CF: complement fixation; CTL: cytotoxic T lymphocytes; CON: control (placebo/unvaccinated); DMRIE/DOPE: [N-(1-(2,3-di- myristyloxypropyl)-N,N-dimethyl-(2-hydroxy-ethyl) ammoniumbromide/dioleoyl) phosphatidylethanola- mine; doi: day of infection; dpi: days post infection; dpv: days post-vaccination; ELISA: enzyme-linked immunosorbent assay; F: female; GM: geometric mean; IgE-bio: biotinylated IgE (IgE-bio); IM: intramuscular; IN: intranasal; M: male (stallion or NR); MEM: minimum essential medium; mo: month; MOG: months of gestation; NR: not reported; NSD: no significant difference; PBL: peripheral blood lymphocytes; PBMC: peripheral blood mononuclear cells; PBS: phosphate buffered saline; PFU: plaque-forming units; PM: pregnant mare; RT: rectal temperature; SE: standard error; SN: serum neutralizing; VN: virus neutralizing; vP1014: vaccinia-based construct (NYVAC) that codes for an immediate early gene (gene 64) of EHV-1; Vx: vaccinates; wk pi: week post-infection.

Supplemental Table 4. GRADE table for experimental studies evaluating the efficacy of live vaccines. Some studies listed here did not provide incidence data that could be used in the exploratory meta-analyses but did have other data relevant to the outcome of interest.

Studies	Type of evidence and initial score	Outcome: Reduction in:	Quality	Consistency	Directness	Precision	Final score and Rating
Bannai 2018; Breathnach 2001; Cornick 1990; Goehring 2010; Goodman 2006; Kydd 2020; Matsumura 1996; Patel 2003a; Patel 2003b; Patel 2004; Perkins 2019; Purdy 1978a; Purdy 1978b; Schnabel 2019; Tsujimura 2009; Van de Walle 2009	Randomized controlled animal studies (n = 5). Nonrandomized, controlled animal studies (n = 11) (Start = 4)	Pyrexia	0	-1[a]	0	-1[b]	2 (low)
Mitchell 1978; Purdy 1978a; Patel et al., 2003a	Nonrandomized, controlled animal studies (Start = 4)	Abortion	-1[c]	-1[a]	0	-1[b]	1 (very low)
Goehring 2010; Goodman 2006; Mitchell 1978; Perkins 2019; Schnabel 2019	Randomized, blinded, controlled animal studies (n = 4). Nonrandomized, controlled animal study (n =1) (Start = 4)	Neurologic signs	0	-1[a]	0	-1[b]	2 (low)
Bannai 2018; Breathnach 2001; Cornick 1990; Goehring 2010; Goodman 2006; Kydd 2020; Matsumura 1996; Patel 2003a; Patel 2003b; Patel 2004; Perkins 2019; Purdy 1978a; Purdy 1978b; Schnabel 2019; Tsujimura 2009; Van de Walle 2009	Randomized controlled animal studies (n = 5). Nonrandomized, controlled animal studies (n = 11) (Start = 4)	Viremia	0	-1[a]	0	-1[b]	2 (low)
Bannai 2018; Breathnach 2001; Cornick 1990; Goehring 2010; Goodman 2006; Kydd 2020; Matsumura 1996; Patel 2003a; Patel 2003b; Patel 2004; Perkins 2019; Purdy 1978a; Purdy 1978b; Schnabel 2019; Tsujimura 2009; Van de Walle 2009	Randomized controlled animal studies (n = 5). Nonrandomized, controlled animal studies (n = 11) (Start = 4)	Nasal shedding	0	-1[a]	0	-1[b]	2 (low)

[a] Downgrade due to considerable heterogeneity

[b] Downgrade due to small sample sizes resulting in underpowered studies and poor estimates of the effect size.

[c] Downgrade due to groups were dissimilar at baseline and other concerns (statistical analyses).

Supplemental Table 5. GRADE table for experimental studies evaluating the efficacy of inactivated vaccines. Some studies listed here did not provide incidence data that could be used in the exploratory meta-analyses but did have other data relevant to the outcome of interest.

Studies	Type of evidence and initial score	Outcome: Reduction in:	Quality	Consistency	Directness	Precision	Final score and Rating
Breathnach 2001; Burrows 1984; Dolby 1995; Goehring 2010; Goodman 2006; Heldens 2001; Kydd 2003; Mumford 1984; Thomson 1979	Randomized controlled animal studies (n = 3). Nonrandomized, controlled animal studies (n = 6) (Start = 4)	Pyrexia	0	-1[a]	0	-1[b]	2 (low)
Burrows 1984; Kydd 2003;	Randomized, controlled animal study (n = 1). Nonrandomized, controlled animal study (n = 1). (Start = 4)	Abortion	-1[c]	0	0	-1[b]	2 (low)
Goehring 2010; Goodman 2006	Randomized, blinded, controlled animal studies (Start = 4)	Neurologic signs	0	-1[a]	0	-1[b]	2 (low)
Breathnach 2001; Burrows 1984; Dolby 1995; Goehring 2010; Goodman 2006; Heldens 2001; Kydd 2003	Randomized controlled animal studies (n = 3). Nonrandomized, controlled animal studies (n = 4). (Start = 4)	Viremia	0	-1[a]	0	-1[b]	2 (low)
Breathnach 2001; Burrows 1984; Dolby 1995; Goehring 2010; Goodman 2006; Heldens 2001; Kydd 2003	Randomized controlled animal studies (n = 3). Nonrandomized, controlled animal studies (n = 4). (Start = 4)	Nasal shedding	0	-1[a]	0	-1[b]	2 (low)

[a] Downgrade due to considerable heterogeneity

[b] Downgrade due to small sample sizes resulting in underpowered studies and poor estimates of the effect size.

[c] Downgrade due to groups were dissimilar at baseline and other concerns (statistical analyses).

Supplemental Table 6. GRADE table for experimental studies evaluating the efficacy of other experimental vaccines including recombinant, DNA, among others. Some studies listed here did not provide incidence data that could be used in the exploratory meta-analyses but did have other data relevant to the outcome of interest.

Studies	Type of evidence and initial score	Outcome: Reduction in:	Quality	Consistency	Directness	Precision	Final score and Rating
Hannant 1993; Minke 2006; Mohd-Azmi 2002; Paillot 2006; Soboll 2006; Wagner 2017	Randomized controlled animal studies (n = 2). Nonrandomized, controlled animal studies (n = 4) (Start = 4)	Pyrexia	0	-1[a]	0	-1[b]	2 (low)
Soboll 2010; Wagner 2017	Nonrandomized, controlled animal studies (Start = 4)	Neurologic signs	-1[d]	0	0	-1[b]	2 (low)
Hannant 1993; Minke 2006; Mohd-Azmi 2002; Paillot 2006; Soboll 2006; Soboll 2010; Wagner 2017	Randomized controlled animal studies (n = 2). Nonrandomized, controlled animal studies (n = 5) (Start = 4)	Viremia	0	-1[a]	0	-1[b]	2 (low)
Hannant 1993; Minke 2006; Mohd-Azmi 2002; Paillot 2006; Soboll 2006; Soboll 2010; Wagner 2017	Randomized controlled animal studies (n = 2). Nonrandomized, controlled animal studies (n = 5) (Start = 4)	Nasal shedding	0	-1[a]	0	-1[b]	2 (low)

[a] Downgrade due to considerable heterogeneity

[b] Downgrade due to small sample sizes resulting in underpowered studies and poor estimates of the effect size.

[c] Downgrade due to groups were dissimilar at baseline and other concerns (statistical analyses).

[d] Downgrade due to uncertainty regarding blinding

SUPPLEMENTAL Figure 2. Forest plot analysis of the overall incidence of early phase pyrexia in EHV-1 infected horses. Individual experimental arms using different vaccines or other experimental conditions are included in this analysis. Missing studies in the forest plot had all events in both intervention and control groups (relative risk = 1). These studies provide no information about relative probability of the event and were automatically omitted from the meta-analysis.

	Study, vaccine, experimental arm	Intervention Events/Total	Controls Events/Total	Relative risk	95% CI	Weight (%)
Commercial MLV	Bannai 2018; Equine rhinopneumonitis vaccine; pooled IM and IN	1/10	3/5	0.167	0.0227 to 1.222	2.51
	Goehring 2010; Rhinomune	8/8	4/4	-		
	Purdy 1978a; Rhinoquin; challenge at 40-50 dpi	0/17	12/12	0.0289	0.00187 to 0.445	1.58
	Purdy 1978a; Rhinoquin; challenge at 1 yr	0/14	3/3	0.0381	0.00243 to 0.596	1.56
	Purdy 1978b; Rhinoquin; experiment 4	0/31	5/5	0.0170	0.00108 to 0.269	1.56
	Purdy 1978b; Rhinoquin; vaccinated at 1-12 days of age	12/12	4/4	-		
	Purdy 1978b; Rhinoquin; vaccinated at 122 days of age	0/7	4/4	0.0694	0.00467 to 1.033	1.61
Killed	Burrows 1984; Pneumabort-K; yearlings	6/6	4/4	-		
	Burrows 1984; Pneumabort-K; 2 yo	12/12	7/7	-		
	Burrows 1984; Pneumabort-K; pregnant mares	14/17	8/9	0.926	0.673 to 1.275	7.17
	Goehring 2010; Pneumabort-K	8/8	4/4	-		
	Heldens 2001; Duvaxyn EHV1,4	4/5	2/4	1.600	0.547 to 4.681	4.76
	Mumford and Bates, 1984; Pneumabort-K	10/30	10/13	0.433	0.241 to 0.780	6.42
Experimental MLV	Matsumura 1996; EHV1 KyA	3/4	2/2	0.840	0.391 to 1.805	5.82
	Patel 2003a; EHV1 C147; pregnant mares	3/5	6/6	0.628	0.310 to 1.274	6.02
	Patel 2003b; EHV1 C147; foals	0/8	7/8	0.0667	0.00444 to 1.002	1.60
	Patel 2004; EHV1 C147	2/8	6/6	0.299	0.102 to 0.875	4.76
	Perkins 2019; EHV-1 Ab4	0/5	5/5	0.0909	0.00633 to 1.306	1.64
	Schnabel 2019; EHV1 Ab4/8	0/8	4/4	0.0617	0.00411 to 0.927	1.60
Deletion mutant	Kydd 2020; RacHΔgM	4/6	6/6	0.692	0.384 to 1.248	6.41
	Perkins 2019; EHV-1 Ab4ΔORF1/71	0/5	5/5	0.0909	0.00633 to 1.306	1.64
	Schnabel 2019; Ab4ΔORF2	1/8	4/4	0.185	0.0417 to 0.822	3.55
	Tsujimura 2009; EHV-1 ΔgE	5/6	3/3	0.898	0.526 to 1.534	6.58
	Van de Walle 2009; rNY03ΔIR6/1gp2S	0/8	4/4	0.0617	0.00411 to 0.927	1.60
DNA	Minke 2006; pooled gB/gC/gD plasmids	20/20	5/5	-		
	Soboll 2006; pooled gB/gC/gD or IE/UL5 plasmid	9/10	5/5	0.942	0.673 to 1.319	7.13
	Dolby 1995; EHV-1 V592	0/5	0/3	-		
Inactivated	Hannant 1993; Immune stimulating complex	7/9	6/6	0.808	0.535 to 1.220	6.94
	Mohd-Azmi 2002; AB4p L or UV inactivated particles	3/3	2/2	-		
	Thomson 1979; pooled Inactivated RAC-H	12/27	11/17	0.687	0.397 to 1.189	6.54
	Wagner 2017; pooled Sav-gC/IL-4	10/10	5/5	-		
Recombinant	Minke 2006; pooled ALVAC-EHV	11/15	4/5	0.917	0.537 to 1.564	6.59
	Paillot 2006; pooled vP1014 and carbomer	2/4	1/1	0.667	0.203 to 2.184	4.40
	Total (random effects)	167/351	161/180	0.468	0.318 to 0.688	100.00

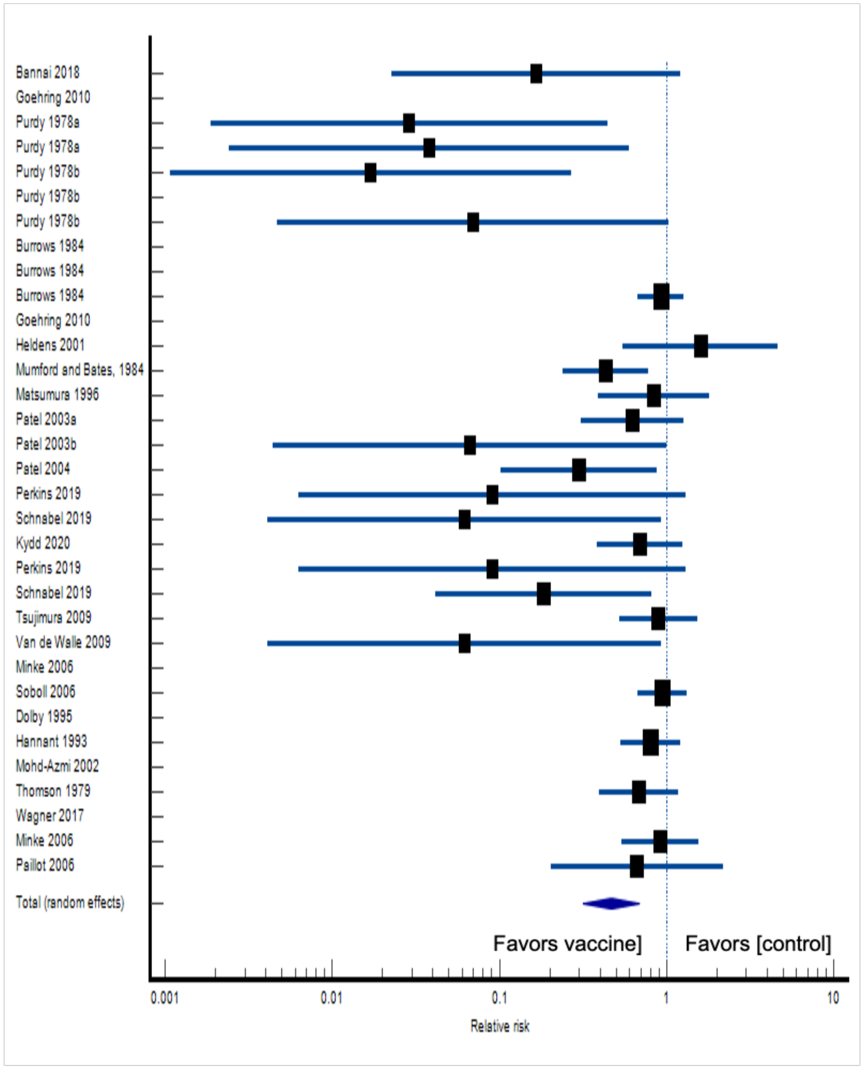

SUPPLEMENTAL Figure 3. Forest plot analysis of the overall incidence of cell-associated viremia in EHV-1 infected horses. Individual experimental arms using different vaccines or other experimental conditions are included in this analysis. Missing studies in the forest plot had all events in both intervention and control groups (relative risk = 1). These studies provide no information about relative probability of the event and were automatically omitted from the meta-analysis.

Group	Study; vaccine; experimental arm	Intervention Events/Total	Controls Events/Total	Relative risk	95% CI	Weight (%)
Commercial MLV	Bannai 2018; Equine rhinopneumonitis vaccine; pooled IM and In	8/10	5/5	0.843	0.564 to 1.259	6.57
	Breathnach 2001; Rhinomune	0/4	1/1	0.133	0.00854 to 2.083	0.93
	Goehring 2010; Rhinomune	4/8	3/4	0.667	0.273 to 1.631	4.31
	Goodman 2006; Rhinomune	5/5	2/2	-		
	Purdy 1978a; Rhinoquin; Challenge at 40-50 d	1/17	12/12	0.0867	0.0187 to 0.403	2.35
	Purdy 1978a; Rhinoquin; Challenge at 1 yr	14/14	3/3	-		
	Purdy 1978b; Rhinoquin; 122 day old foals	0/7	4/4	0.0694	0.00467 to 1.033	0.96
	Purdy 1978b; Rhinoquin; 7-42 day old foals	6/7	4/4	0.903	0.580 to 1.406	6.39
	Purdy 1978b; Rhinoquin; 1-12 day old foals	12/12	4/4	-		
Killed	Breathnach 2001; Pneumabort K	0/4	1/1	0.133	0.00854 to 2.083	0.93
	Burrows 1984; Pneumabort-K; yearlings	6/6	4/4	-		
	Burrows 1984; Pneumabort-K; 2 yo	12/12	7/7	-		
	Burrows 1984; Pneumabort-K; pregnant mares	15/17	5/7	1.235	0.750 to 2.036	6.13
	Goehring 2010; Pneumabort-K	2/8	3/4	0.333	0.0884 to 1.256	2.85
	Goodman 2006; Flu- vac Innovator 6	5/5	2/2	-		
Experimental MLV	Heldens 2001; Duvaxyn EHV1,4; Foals	3/10	4/5	0.375	0.132 to 1.064	3.74
	Heldens 2001; Duvaxyn EHV1,4; pregnant mares	5/5	4/4	-		
	Breathnach 2001; Rhinomune and Pneumabort K	0/4	1/1	0.133	0.00854 to 2.083	0.93
	Breathnach 2001; EHV1 Army 183	0/4	1/1	0.133	0.00854 to 2.083	0.93
	Matsumura 1996; EHV1 KyA	0/4	2/2	0.120	0.00825 to 1.746	0.98
	Patel 2003a; EHV1 C147	4/6	6/6	0.692	0.384 to 1.248	5.70
	Patel 2003b; EHV1 C147	6/8	8/8	0.765	0.495 to 1.182	6.42
	Patel 2004; EHV1 C147)	4/8	6/6	0.538	0.271 to 1.068	5.25
	Perkins 2019; EHV-1 Ab4	0/5	2/2	0.0972	0.00661 to 1.430	0.97
Deletion mutant	Schnabel 2019; EHV1 Ab4/8	3/8	4/4	0.432	0.181 to 1.031	4.42
	Kydd 2020; RacHΔgM	6/6	6/6	-		
	Perkins 2019; EHV-1 Ab4ΔORF1/71	2/5	2/2	0.486	0.172 to 1.374	3.75
	Schnabel 2019; Ab4ΔORF2	1/8	4/4	0.185	0.0417 to 0.822	2.45
	Tsujimura 2009; EHV-1 ΔgE	5/6	3/3	0.898	0.526 to 1.534	5.96
	Van de Walle 2009; rNY03ΔIR6/1gp2S	0/8	4/4	0.0617	0.00411 to 0.927	0.96
DNA	Minke 2006; gB/gC/gD plasmids	19/20	5/5	1.013	0.774 to 1.325	7.09
	Soboll 2006; gB/gC/gD or IE/UL5 plasmids	8/10	1/5	4.000	0.674 to 23.726	1.90
Inactivated	Dolby 1995; EHV-1 V592	0/5	3/3	0.0952	0.00653 to 1.388	0.97
	Mohd-Azmi et al., 2002 (AB4p L particles/UV inactivated)	2/2	2/2	-		
	Wagner 2017; pooled Sav-gC/IL-4	5/5	5/5	-		
Recombinant	Minke 2006; pooled ALVAC-EHV	15/15	4/5	1.292	0.807 to 2.067	6.26
	Paillot 2006; vP1014 and carbomer	3/4	1/1	0.933	0.349 to 2.498	3.95
	Soboll 2010; rMVA-IE; pooled all haplotypes	9/21	5/5	0.471	0.275 to 0.806	5.95
	Total (random effects)	190/313	143/153	0.590	0.444 to 0.783	100.00

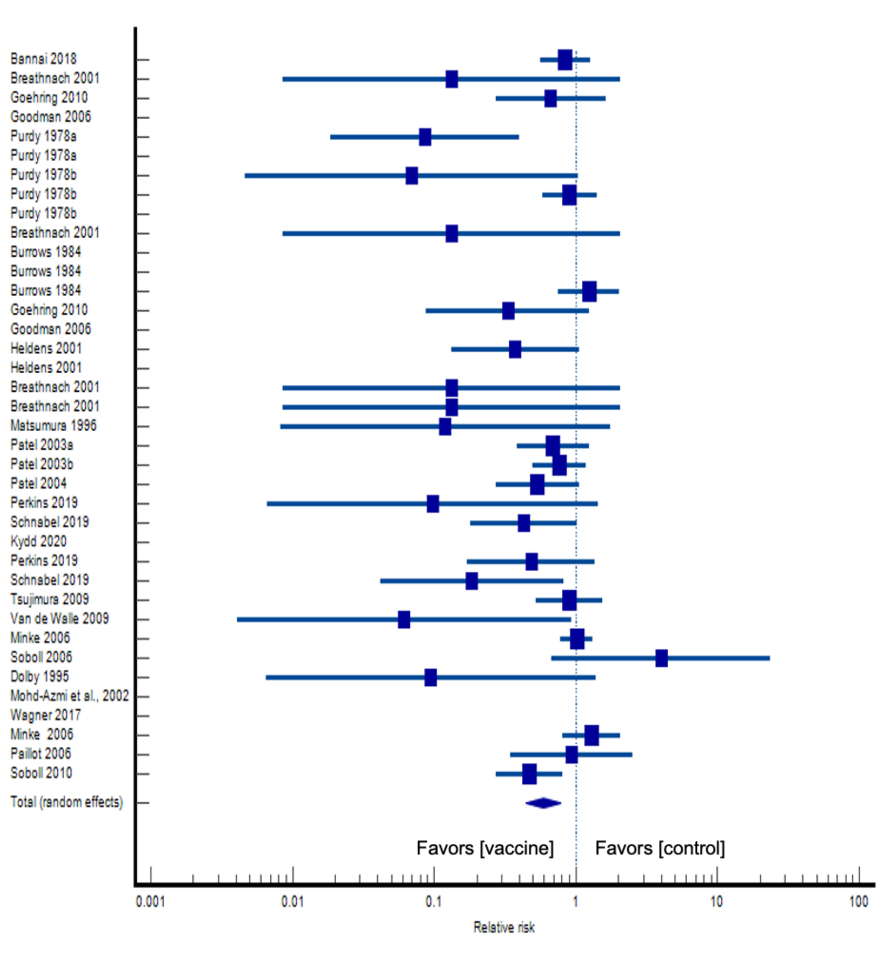

SUPPLEMENTAL Figure 4. Forest plot analysis of the overall incidence of nasal shedding in EHV-1 infected horses. Individual experimental arms using different vaccines or other experimental conditions are included in this analysis. Missing studies in the forest plot had all events in both intervention and control groups (relative risk = 1). These studies provide no information about relative probability of the event and were automatically omitted from the meta-analysis.

	Study; vaccine; experimental arm	Intervention Events/Total	Controls Events/Total	Relative risk	95% CI	Weight (%)
Commercial MLV	Bannai 2018; Equine rhinopneumonitis vaccine; pooled IM and IN	8/10	5/5	0.843	0.564 to 1.259	7.20
	Goodman 2006; Rhinomune	1/5	2/2	0.292	0.0684 to 1.244	2.30
	Purdy 1978a; Rhinoquin; Challenge at 1 yr	1/14	3/3	0.114	0.0239 to 0.545	2.06
	Purdy 1978a; Rhinoquin; Challenge at 40-50 d	0/17	12/12	0.0289	0.00187 to 0.445	0.80
	Purdy 1978b; Rhinoquin; 1-12 day old foals	12/12	4/4	-		
	Purdy 1978b; Rhinoquin; 122 day old foals	0/7	2/4	0.125	0.00743 to 2.104	0.75
	Purdy 1978b; Rhinoquin; 7-42 day old foals	6/7	4/4	0.903	0.580 to 1.406	6.93
Killed	Goodman 2006; Flu- vac Innovator 6	5/5	2/2	-		
	Heldens 2001; Duvaxyn EHV1,4; foals	10/10	5/5	-		
	Heldens 2001; Duvaxyn EHV1,4; pregnant mares	5/5	4/4	-		
	Mumford and Bates, 1984; Pneumabort-K; pooled 2 or 3 doses	23/30	10/13	0.997	0.697 to 1.425	7.47
Experimental MLV	Matsumura 1996; EHV1 KyA	1/4	2/2	0.360	0.0860 to 1.506	2.35
	Patel 2003a; EHV1 C147	4/6	9/11	0.815	0.434 to 1.531	5.72
	Patel 2003b; EHV1 C147	6/8	8/8	0.765	0.495 to 1.182	6.98
	Patel 2004; EHV1 C147)	7/8	6/6	0.897	0.628 to 1.283	7.47
	Perkins 2019; EHV-1 Ab4	0/5	2/2	0.0972	0.00661 to 1.430	0.83
	Schnabel 2019; EHV1 Ab4/8	3/8	4/4	0.432	0.181 to 1.031	4.36
Deletion mutant	Cornick 1990; TK-EHV-1 (H6b mutant)	8/8	4/4	-		
	Perkins 2019; EHV-1 Ab4ΔORF1/71	0/5	2/2	0.0972	0.00661 to 1.430	0.83
	Schnabel 2019; Ab4ΔORF2	1/8	4/4	0.185	0.0417 to 0.822	2.21
	Tsujimura 2009; EHV-1 ΔgE	6/6	3/3	-		
DNA	Van de Walle 2009; rNY03ΔIR6/1gp2S	8/8	4/4	-		
	Minke 2006; gB/gC/gD plasmids	17/20	5/5	0.909	0.668 to 1.237	7.76
	Soboll 2006; pooled gB/gC/gD and IE/UL5 plasmids	8/10	4/5	1.000	0.585 to 1.710	6.32
Inactivated	Dolby 1995; EHV-1 V592	0/5	3/3	0.0952	0.00653 to 1.388	0.83
	Hannant 1993; Immune stimulating complex	3/10	4/5	0.375	0.132 to 1.064	3.57
	Mohd-Azmi et al., 2002; pooled AB4p L particles/UV inactivated	2/2	2/2	-		
	Thomson 1979; inactivated RAC-H	5/6	3/3	0.898	0.526 to 1.534	6.33
	Wagner 2017; pooled Sav-gC/IL-4	5/5	5/5	-		
Recombinant	Minke 2006; pooled ALVAC-EHV	15/15	4/5	1.292	0.807 to 2.067	6.76
	Paillot 2006; vP1014 and carbomer	3/4	1/1	0.933	0.349 to 2.498	3.82
	Soboll 2010; rMVA-IE; pooled all haplotypes pooled	9/21	5/5	0.471	0.275 to 0.806	6.32
	Total (random effects)	182/294	134/142	0.661	0.512 to 0.855	100.00

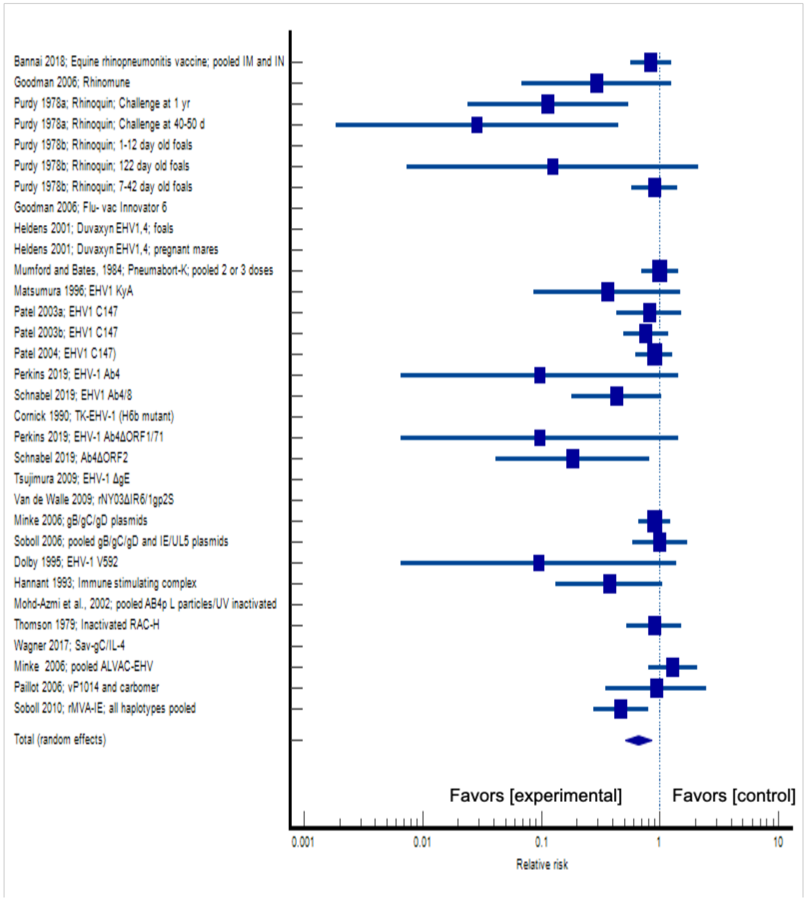

References

1. Hooijmans CR, Rovers MM, de Vries RB, et al. SYRCLE's risk of bias tool for animal studies. BMC Med Res Methodol 2014;14:43.

2. Guyatt GH, Oxman AD, Vist GE, et al. GRADE: an emerging consensus on rating quality of evidence and strength of recommendations. BMJ 2008;336:924-926.

3. Guyatt GH, Oxman AD, Schunemann HJ, et al. GRADE guidelines: a new series of articles in the Journal of Clinical Epidemiology. J Clin Epidemiol 2011;64:380-382.
